# Combined LC-MS-based metabolomics and GC-IMS analysis reveal changes in chemical components and aroma components of Jujube leaf tea during processing

**DOI:** 10.3389/fpls.2023.1179553

**Published:** 2023-05-17

**Authors:** Nan Jiang, Shujuan Hou, Yuye Liu, Peixing Ren, Nuoyu Xie, Ye Yuan, Qing Hao, Mengjun Liu, Zhihui Zhao

**Affiliations:** ^1^ College of Horticulture, Hebei Agricultural University, Baoding, Hebei, China; ^2^ Research Center of Chinese Jujube, Hebei Agricultural University, Baoding, Hebei, China; ^3^ Institute of Horticultural Crops, Xinjiang Academy of Agricultural Sciences, Urumqi, Xinjiang, China

**Keywords:** jujube leaf, jujube leaf tea, metabolite, aroma components, amino acids, lipids

## Abstract

Making tea from jujube leaves changed the chemical composition and aroma composition of jujube leaves. Here, Through LC-MS, GC-IMS, and GC-MS technology, we have revealed the effect of jujube leaf processing changes on metabolites. LC-MS identified 468 non-volatile metabolites, while GC-IMS and GC-MS detected 52 and 24 volatile metabolites, respectively. 109 non-volatile metabolites exhibiting more pronounced differences were screened. Most lipids and lipid-like molecules, organic acids, amino acids, and flavonoids increased significantly after processing. GC-IMS and GC-MS analysis revealed that the contents of aldehydes and ketones were significantly increased, while esters and partial alcohols were decreased after processing into jujube leaf tea. The main flavor substances of fresh jujube leaf and jujube leaf tea were eugenol and (E) - 2-Hexenal, respectively. Furthermore, amino acids and lipids were closely linked to the formation of volatile metabolites. Our study provided new insights into the changes in metabolites of jujube leaves processed into jujube leaf tea, and had great potential for industrial application. It laid a foundation for further research on fruit tree leaf tea.

## Highlights

• Analysis of metabolites by LC-MS, GC-IMS, and GC-MS.

• LC-MS identified 468 non-volatile metabolites, while GC-IMS and GC-MS detected 52 and 24 volatile metabolites, respectively.

• Glycerophospholipid, tryptophan, and linoleic acid metabolism pathways were significantly enriched.

• Significant changes in metabolites during the processing of jujube leaves.

• Amino acids and lipids are closely related to the formation of volatile metabolites.

## 1 Introduction

Jujube (*Ziziphus jujuba* Mill.) is China’s economically significant fruit tree. It has an enormous cultivation scale and the highest economic and ecological value among the species of the Rhamnaceae family worldwide. Jujube has been domesticated and cultivated in China for more than 7000 years. It is mainly distributed in the Northwest (Xinjiang, Gansu), the Yellow River Basin (Ningxia, Shaanxi, Shanxi), and the eastern region (Shandong, Hebei, Henan) (Liu et al., 2020). Research has shown that jujube leaves are rich in flavonoids, polysaccharides, amino acids, vitamins, and other nutrients (Zhao et al., 2008; Zhang et al., 2018). It has antioxidant, anti-aging, sedative, and hypnotic activities (Zemouri-Alioui et al., 2019; Hua et al., 2022). Jujube leaves have yet to be adequately commercially exploited in the production and health industry. They are usually used as livestock feed or for natural degradation, a massive waste of jujube leaf resources. One innovative technique for utilizing jujube leaves is to make tea. And some green teas have made from fruit tree leaves with important health-promoting properties such as sea buckthorn leaf tea, mulberry leaf tea, guava leaf tea, and persimmon leaf tea. (Sakanaka et al., 2005, Deguchi and Miyazaki, 2010, Lee et al., 2011; Wilson and Islam, 2015).

Tea is the second largest beverage consumed in the world after water, with much higher consumption than coffee, beer, and wine(Rietveld and Wiseman, 2003). Green tea is one of the most popular tea beverages in China. Due to its green leaves, clear soup, aroma, fresh taste, and other unique sensory qualities, green tea is increasingly favored by consumers. The functional properties of green tea are mainly determined by non-volatile metabolites, while volatile metabolites mainly determine the aroma. Regarding tea soup freshness, amino acids are the essential compounds underlying it. On the other hand, flavone glycosides and catechins give bitterness (Scharbert and Hofmann, 2005; Zhang, 2016), soluble sugars give sweetness (Das et al., 2019), organic acids contribute significantly to acidity and fruitiness, and lipids are conducive to shape of the tea. Jujube leaves do not contain caffeine, theophylline, and other stimulating compounds but are rich in flavonoids and other substances with sedative and hypnotic effects (Zhang et al., 2014). Therefore, jujube leaf tea soup consumption at night may improve sleep quality probably.

Extensive targeted metabolomic analysis can simultaneously quantify hundreds of known and 1000 known and unknown metabolites when combined techniques are combined (Sawada et al., 2009). It has applications in multiple fields due to its high throughput, high sensitivity, wide-coverage, and other advantages (Wang et al., 2018, Zhang et al., 2018). Currently, widely targeted metabolomics has been used to identify and analyze metabolite biosynthesis (Albinsky et al., 2010; Zou et al., 2020). Metabolite changes during tea making, such as white tea, green tea, black tea, and oolong tea, are also commonly used in metabonomics (Dai et al., 2017; Cheng et al., 2020; Wu et al., 2020; Xu et al., 2021). Our study aimed to detect the non-volatile and volatile metabolites in jujube leaves and jujube leaf tea through extensively targeted metabolomics, GC-IMS and GC-MS technology. Orthogonal partial least squares discriminant analysis (OPLS-DA), heat map, and other statistical analysis approaches were used to evaluate metabolite changes before and after jujube leaf processing and to explore the relationship between non-volatile metabolites and volatile metabolites. Our results provide essential leads for developing jujube leaf tea and improving the quality and efficiency of the jujube industry.

## 2 Materials and methods

### 2.1 Experimental materials

**Figure 1:**
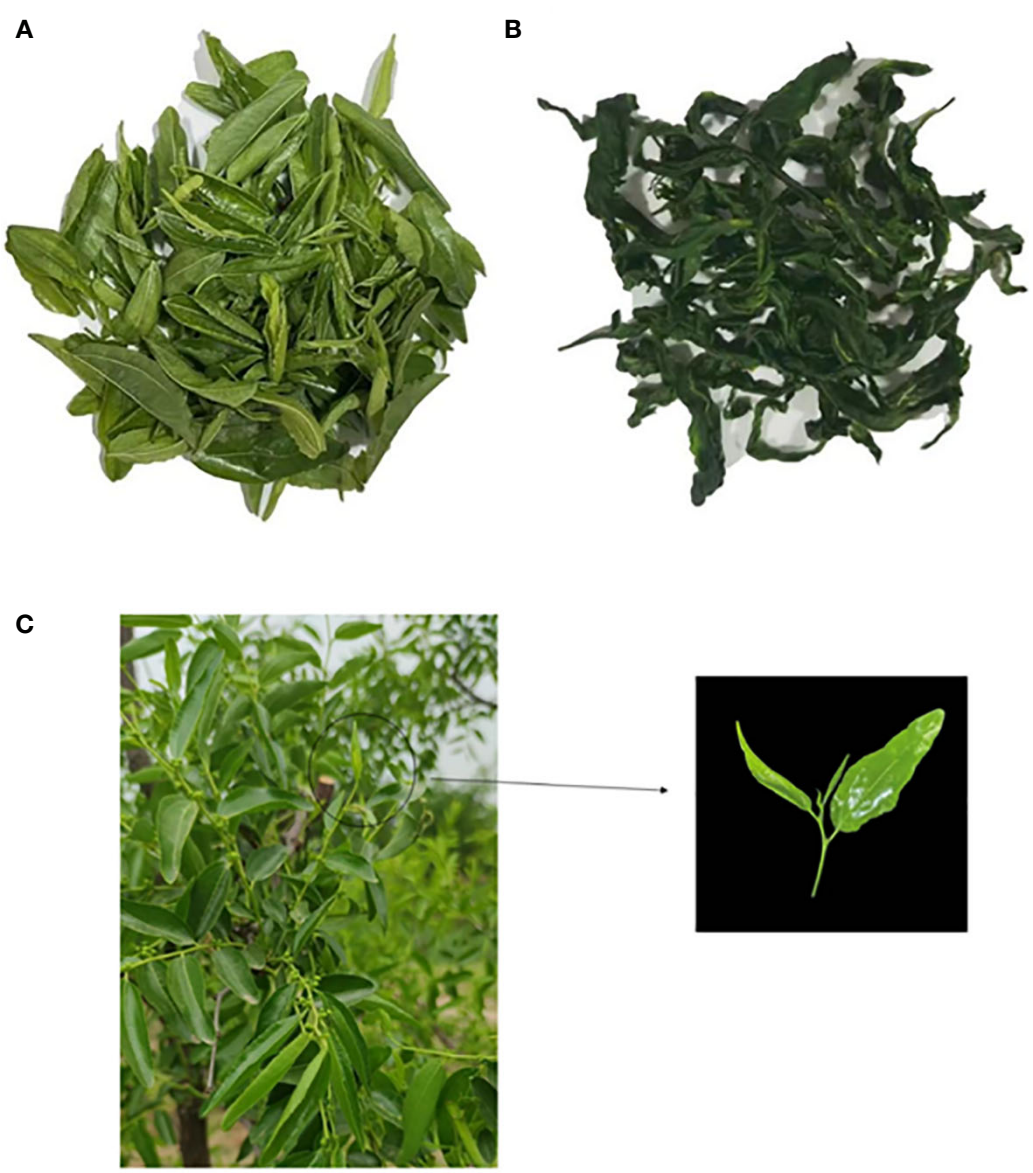
**(A)** D1, Fresh jujube leaves; **(B)** D2, jujube leaf tea; **(C)** Jujube leaves used in green tea processing (Left: the fruit bearing shoots, Right: the leaves used in green tea processing).

Leaves from the *Ziziphus jujuba* Mill. cv. Dongzao were used, and the picking standard is two leaves and one bud on the fruit-bearing shoot (**Figure 1C**). The leaves were collected in June 2022 in Baoding (115° 47’E; 38° 87’N) (Hebei Province, China). Liquid chromatography grade solvents methanol and acetonitrile were purchased from Fisher Chemical, 2-propanol from Merck, formal acid from CNW, and 2-Chloro-L-phenylalanine (≥98%) from Adamas-beta. Alanine, arginine, asparagine, aspartic acid, cystine, glutamine, glutamic acid, glycine, histidine, isoleucine, L-cysteine, leucine, L-hydroxyproline, L-tryptophan, lysine, methionine, phenylalanine, proline, serine, threonine, tyrosine, valine, and 1,3-dichlorobenzene were all analytically pure and purchased from Beijing Solarbio Science&Technology Co., Ltd.

### 2.2 Jujube leaf green tea processing

#### 2.2.1 Harvesting

Select intact leaves with fresh, bright color and no disease spots and pests on the fruit-bearing shoot (D1). Avoid picking weeds or petioles.

#### 2.2.2 Cleaning

Wash the jujube leaves with clean water to remove the dust from the leaf surface.

#### 2.2.3 Spreading

Spread the leaves at room temperature (26 °) for 10-12h until they lose luster and their water content drops to 70%. When the leaves are pinched, they feel soft, and their grass smell is weakened.

#### 2.2.4 Fixing

Fixing leaves should be done to expose aroma, reduce the grass smell, and change the color from fresh green to dark green. The leaves are pinched tightly into clusters and become slightly sticky. The pot fixation method was used in this study. The parameters were as follows: a pan was placed on an induction cooker, the temperature was adjusted to 120 °, and the pan was preheated for 3min. Then the leaves were placed in the pan and fixed for the 60s by grasping, throwing, and shaking.

#### 2.2.5 Rolling

Spread the sticky jujube leaves on the chopping board. After the leaf temperature is reduced, they are rolled into strips using both hands while they are kneaded clockwise. If too much strength is used, it is easy to crush the tea. After rolling for a few minutes, they are spread and continue kneaded into strips. Kneading is in the order of “light heavy light.” The total kneading time is about 0.5h. Kneading should be done until the leaves can loosen naturally, and their color becomes dark green and bright.

#### 2.2.6 Drying

Return the rolled jujube leaves to the pan at about 90 ° until they are dried. To ensure that the dry tea leaves remain in tight and curly strips, it is necessary to knead, break up, shake and stir continuously when drying. At the same time, the pan temperature is gradually reduced with the evaporation of water from the leaves. Finally, it is stabilized at about 40 °, and the tea aroma gradually increased. This is the jujube leaf tea product (D2). D1, D2, and fruit-bearing shoots are shown in **Figure 1**.

### 2.3 Analysis of non-volatile metabolites of jujube leaf tea before and after processing

#### 2.3.1 Sample preparation and extraction

Grinding jujube leaves with liquid nitrogen, 50mg dry samples of jujube leaves before and after processing were accurately weighed and placed into a 1.5ml centrifuge tube, adding a 6 mm diameter grinding bead. 400 µl of extract (methanol: water =4:1 (v:v)) was added, containing 0.02 mg/ml of internal standard (l-2-chlorophenyl alanine). The frozen tissue samples were ground for 6 minutes (-10 °, 50 Hz). A low-temperature ultrasonic extraction was performed for 30 minutes (5 °, 40KHz), and then the samples were let to stand at -20 ° for 30 minutes. They were then centrifuged for 15min (13000g, 4 °), and the supernatant was transferred t to the injection vial with internal intubation for the analysis. In addition, 20 µl supernatant was taken from each sample and was mixed to be used as a quality control sample. It was used to monitor deviations in the analysis results and identify potential errors caused by the analytical instrument.

#### 2.3.2 LC-MC conditions

The LC-MS instrument platform used was the HPLC-Q executive HF-X system (Thermo Scientific). The chromatographic conditions were as follows: the chromatographic column used was the ACQUITY UPLC HSS T3 (100 mm × 2.1 mm i.d., 1.8 µm; Waters, Milford, USA); mobile phase A was 95% water +5% acetonitrile (containing 0.1% formic acid); mobile phase B was 47.5% acetonitrile +47.5% isopropanol +5% water (containing 0.1% formic acid); the injection volume was 2 μL. The column temperature was 40 °. Elution procedure: 0min, 100% of phase A; 3.5min, 75.5% of phase A; 5min, phase A 35%; 5.5min, phase A 0%; 7.4min, phase A 0%; 7.6min, phase A 48.5%; 7.8min, 100% of phase A; 19min, phase A 100%.

Mass spectral analysis conditions: electrospray spray ion source (ESI) was used to collect mass spectrum signals through positive and negative ion scanning modes. The scan type was 70~1050 m/z, the sheath gas flow rate was 50 arb, the aux gas flow rate was 13 arb, the heater temperature was 425 °, and the capillary temperature was 325 °. The spray voltage was 3500 v and -3500 v in positive and negative ion modes, respectively.

#### 2.3.3 Quantitative analysis of amino acids

Mixed 50mg of dry sample with 750µL of acetonitrile, sonicated for 30 minutes, then centrifuged the extract at 13000 rpm for 5 minutes, repeat once, and took the supernatant for LC-MS/MS detection. The LC-MS/MS system consisted of ultra-high performance liquid chromatography(ExionLC AD system) and mass spectrometry(AB SCIEX QTRAP 6500+), using a liquid chromatography column maintained at 35 °(Waters BEH Amide,100*2.1 mm,1.7 μm). The mobile solutions were water with 0.4% formic acid (A) and acetonitrile containing 0.4% formic acid (B). The chromatographic gradient table was shown in **Table 1**, the mass spectrometry condition adopted positive mode detection, with Curtain Gas of 35 psi and Collision Gas was Medium, the ionspray voltage was set to 5500 V, the source temperature was set to 350°, the nebulizer and heater gases were maintained at 70 psi.

**Table 1:** Chromatographic gradient table.

Time(min)	Velocity of flow(mL/min)	A%	B%
0	1	100	0
1	1	90	10
2.6	1	85	15
3.5	1	70	30
4	1	70	30
4.1	1	100	0
6	1	100	0

### 2.4 GC-IMS analysis

GC-IMS analysis of volatile compounds in jujube leaves before and after processing was performed according to the method of Wang et al. (Wang et al., 2021). The FlavorSpec ^®^ Flavor analyzer was used to measure the volatile headspace components in the samples. The fresh jujube leaves were cut, and the jujube leaf tea was ground; 1 g was accurately weighed from each and placed into a 20ml headspace bottle and on the machine after sealing. The headspace incubation temperature was 80 °, the incubation time was 15min, and the incubation speed was 500 rpm. The injection volume was 200ul, and the injection needle temperature was set at 85 °. Gas phase ion migration conditions were: MXT-WAX column, 30 meters, 0.53 mm ID, 1.0 μm df (RESTEK company, USA), column temperature 80 °, IMS temperature 45 °, analysis time 30min; The carrier gas was high-purity nitrogen (purity ≥ 99.999%), rate of 150 mL/min, and used the following programmed flow: 2 mL/min for 2 min,10 mL/min for 8 min, and raised to 100 mL/min within 10 min, and then held for 10 min.

### 2.5 GC-MS analysis

Placed a dry sample (1g) and 1,3-dichlorobenzene (50mL, 0.5mg/mL) as the internal standard into an extraction bottle, equilibrated in a 100 ° water bath for 10 minutes, exposed the SPME fiber in the top space of the sample, performed volatile absorption for 45 minutes at 60 °, and then immediately inserted it into the GC(Agilent, USA) injection port (250 °) for desorption for 5 minutes. The heating procedure was as follows: maintained at 50 ° for 3 minutes, raised to 120 ° at 4 °/min, maintained for 8 minutes, then raised to 200 ° at 4 °/min, maintained for 3 minutes, and finally raised to 250 ° at 10 °/min, maintained for 3 minutes. Helium is used as the carrier gas, with a flow rate of 1.0mL/min and a split ratio of 1:60. The MS ion source temperature was maintained at 230 °. The GC-MS data detection results were retrieved and identified through the NIST14.0 spectral library. The concentration of each volatile substance was calculated according to equation (1) based on the peak area of the internal standard substance, and the OAV value of the volatile substance was calculated according to equation (2).


(1) $$C_{i}(\text{mg}/\text{L})=\frac{A0}{Ais}*C_{is}$$

$C_{i}\;$ was the concentration of the compound, $C_{is\;}was$ the final concentration of the internal standard in the sample, $A_{0}\;$ was the peak area of the compound, and $A_{is}$ was the peak area of the internal standard.


(2) $$\text{OAV}(µ\text{g}/\text{L})=\frac{Ci}{OTi}$$

$C_{i}\;$ was the concentration of the compound, $OT_{i}$ was the aroma threshold of the compound.

### 2.6 Statistical analysis

The experiments were repeated independently three times, and the results from each experiment were the average of three replicates. The raw data were imported into the metabonomics processing software ProgenesisQI (Waters Corporation, Milford, USA) for baseline filtering, peak recognition, integration, retention time correction, and peak alignment. Finally, a data matrix containing information such as retention time, mass charge ratio, peak intensity, etc., was obtained. The software identified the characteristic peak library, and the MS and MS/MS mass spectrometry information was matched with the respective metabolic database. The MS mass error was set to less than 10 ppm, and the metabolites were identified according to their secondary mass spectrometry matching score. Multivariate statistical analysis will be conducted for the planned metabolite data set. The hypergeometric distribution algorithm was used to obtain the pathway of metabolite significant enrichment in the metabolic data set, and the BH method was used to correct the P value. When the corrected P value was<0.05, the pathway was considered to have significant enrichment.

The Vocal volatile component analysis software was used to view spectra and data’s qualitative and quantitative analysis. The built-in NIST database and IMS database can carry out qualitative analyses of the compounds. The Reporter plug-in was used to directly compare the spectrum differences between samples (three-dimensional spectrum, two-dimensional top view, and difference spectrum). The Gallery plot plug-in was used for fingerprint comparison and quantitative comparison of volatile organic compounds between different samples.

## 3 Results and discussion

### 3.1 Non-volatile metabolite analysis of jujube leaf tea before and after processing

#### 3.1.1 LC-MS non-targeted metabolome analysis of jujube leaf tea before and after processing

**Figure 2:**
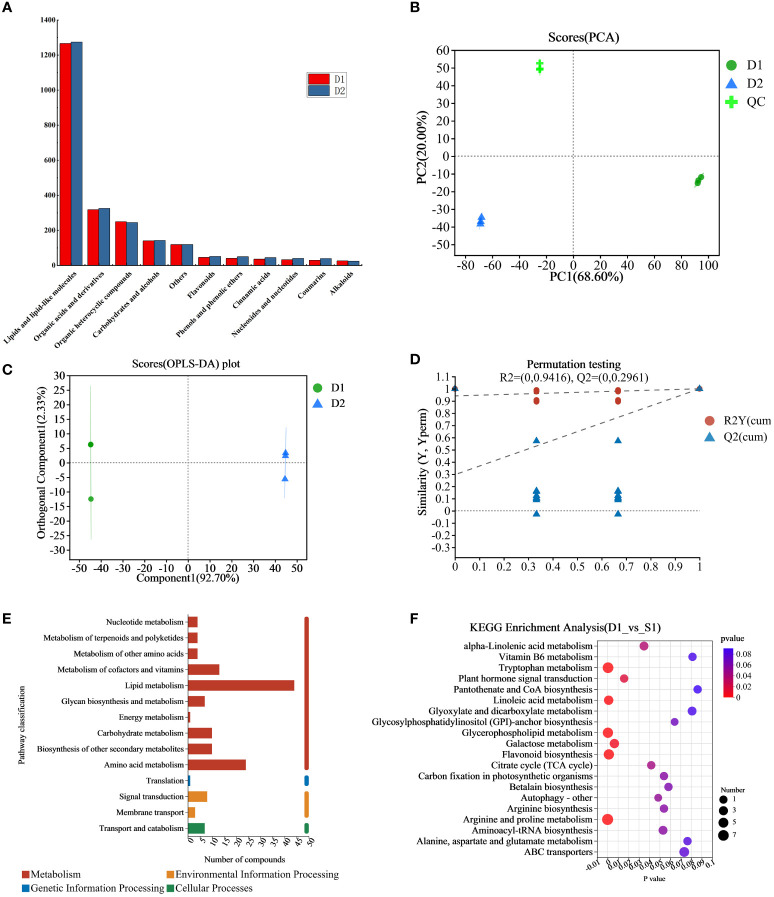
**(A)** Expression map of 11 metabolites in D1 and D2, **(B)** The PCA scores plot of the D1 group, the D2 group and the QC group, **(C)** OPLS-DA score plots of the D1 group versus the D2 group. **(D)** Displacement check diagram for D1 and D2. **(E)** D1 and D2 classification diagram of KEGG metabolic pathway, the ordinate is the second classification of KEGG metabolic pathway, and the abscissa is the number of metabolites annotated to this pathway. **(F)** D1 and D2 KEGG enrichment analysis bubble diagram, the abscissa is the enrichment significance p-value, and the ordinate is the KEGG pathway. The bubble size in the figure represents the number of metabolites enriched in the pathway. D1, Fresh jujube leaves; D2, Jujube leaf tea; QC, the quality control samples.

In this study, we analyzed and compared the metabolome of fresh jujube leaf and jujube leaf tea. We detected 761 peaks and identified 468 metabolites (**Supplementary Materials Table S1**), including 245 lipids and lipid-like molecules and 68 organic acids and their derivatives. Furthermore, 53 organic heterocyclic compounds were identified, such as furan, indole, and pyridine, as well as 28 carbohydrates and alcohols, 10 phenols and phenolic ethers, 9 flavonoids, 8 cinnamic acids, 8 coumarins, 7 nucleosides and nucleotides, 5 alkaloids, and their derivatives, and 27 other metabolites. The changes in classified non-volatile metabolites before and after processing were observed. As shown in **Figure 2A**, the total amount of non-volatile metabolites shows the following trend: D2>D1. The highest expression amounts are lipids and lipid-like molecules, organic acids and their derivatives, organic heterocyclic compounds, carbohydrates, and alcohols. After processing, the expression of 11 metabolites (except organic heterocyclic compounds and others) in jujube leaves increased to varying degrees, indicating that the nutritional value of jujube leaf tea was higher than that of jujube leaves.

The samples were comprehensively analyzed using unsupervised PCA statistical tools (including the quality control (QC) samples). As shown from the PCA score chart (**Figure 2B**), principal component 1 contributed 68.60% of the total variation, and principal component 2 contributed 20.00%, respectively, with all samples within the 95% confidence interval. QC samples, fresh jujube leaf, and jujube leaf tea samples were clustered into three groups. Therefore significant differences between the groups require further study. To quickly and accurately analyze the differences between groups, the OPLS-DA model with supervision function was used for analysis. The OPLS-DA scoring chart results (**Figure 2C**) showed that fresh jujube leaves and jujube leave tea samples were distributed on the left and right sides of the 95% confidence interval. The discrimination effect was obvious, indicating that the composition of the two samples was significantly different, attributed to differentially accumulated metabolites. The model parameters were two principal components, and its cumulative prediction rate was Q2 = 1, R2X=0.95, and R2Y=1, the regression lines of R2 and Q2 showed a downward trend as the replacement retention decreases(**Figure 2D**), indicating that the replacement test had passed and the model was reliable.

We brought the metabolites into KEGG database for classification and analysis of related pathways. The results showed that most metabolites were classified into “metabolism” which we expected (**Figure 2E**). The main metabolic pathways were lipid metabolism and amino acid metabolism. Subsequently, we conducted KEGG pathway enrichment analysis to identify differences in metabolic pathways between D1 and D2. As shown in **Figure 2F**, there were significant differences (p<0.05) in 11 metabolic pathways between D1 and D, and the top three were glycerophospholipid metabolism, tryptophan metabolism, and linoleic acid metabolism.

#### 3.1.2 Dynamic changes in non-volatile metabolites of Jujube leaf tea before and after processing

**Figure 3:**
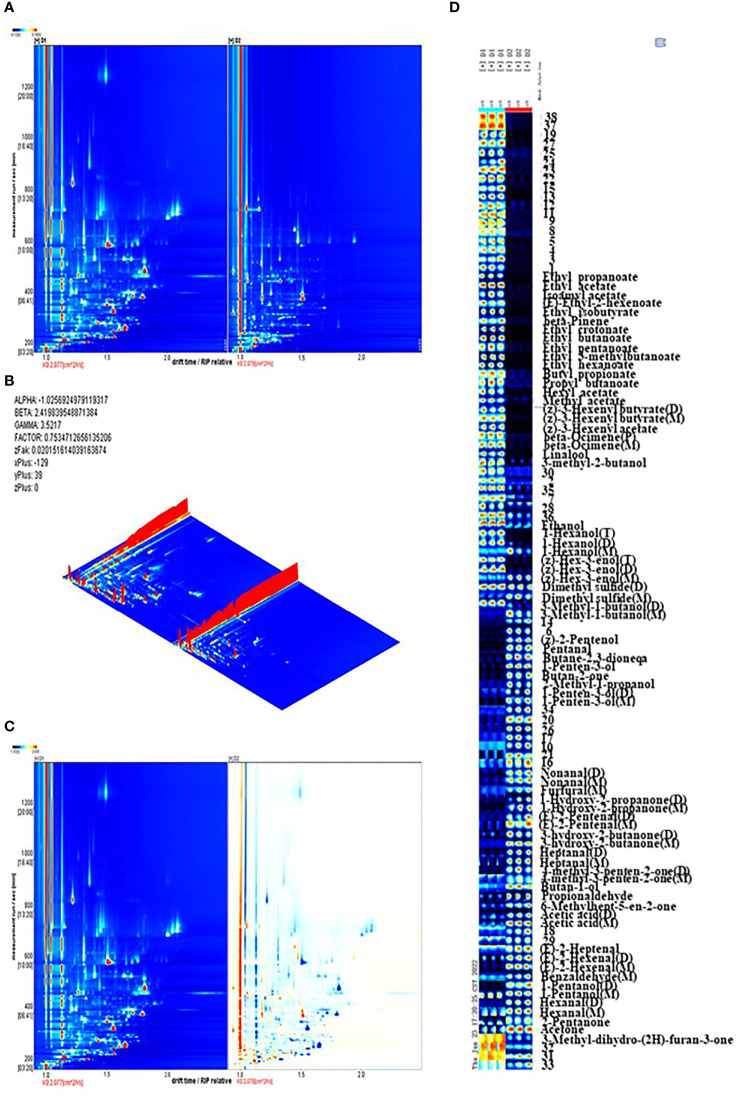
**(A)** Volcanic map of differential metabolites in D1 and D2, **(B)** Pie chart of the number of different types of 107 specific non-volatile metabolites in D1 vs D2, **(C)** and Heat maps of 107 different nonvolatile metabolites of jujube leaf tea before and after processing. D1, Fresh jujube leaves; D2, Jujube leaf tea. Each contains three repeats. **(D)** Thermogram of four types of non-volatile components in jujube leaf tea before and after processing. D1, Fresh jujube leaves; D2, Jujube leaf tea. Each contains three repeats.

Different metabolites were screened through a t-test, based on the parameters VIP > 1, p<0.05, fold change ≥ 1.5 or ≤ 0.5, to clarify the specific differences in chemical components of jujube leaves before and after processing. A total of 107 compounds with significant differences between groups were screened (**Supplementary Materials Table S2**), including 94 up-regulated and 13 down-regulated (**Figure 3A**). 107 differentially accumulated metabolites were identified, including 26 organic acids and derivatives, 18 amino acids and their derivatives, 17 lipids and lipid-like molecules, 11 furan, indole, pyridine, and other organic heterocyclic compounds, 8 carbohydrates and other organic oxygen compounds, 6 flavonoids, 5 phenol, and phenyl ether, 3 coumarins and derivatives, and 15 other metabolites (**Figure 3B**). To investigate the changes between the different metabolites more intuitively, a heat map of the 109 differentially accumulated metabolites was constructed (**Figure 3C**), the metabolites of D1 and D2 showed significant difference. Four important metabolites were selected for specific analysis, as shown in **Figure 3D**. Compared with D1, D2 has significantly increased the content of organic acids and their derivatives, lipids, and lipid-like molecules, and the difference becomes more significant, while the content of amino acids and their derivatives, flavonoids has little difference, almost no difference. The results are described below.

##### 3.1.2.1 Organic acids

Organic acids can inhibit bitter and sour tastes and give fruity tastes and flavors. They are critical intermediate products of carbohydrate catabolism (HE and SUN, 2015). After jujube leaves are processed into tea, the organic acid content generally increases. Among them, hydroxyoctanoic acid and chorismic acid content changed more prominently, increasing by about 50%. Chorismic acid is an intermediate of aromatic amino acids biosynthesis in plants. It is a compound produced at a key metabolic bifurcation point, significantly contributing to the formation of tryptophan(Gibson and Gibson, 1964). As a result, the tryptophan content in D2 increased significantly.

##### 3.1.2.2 Lipids and lipid-like molecules

Lipids are hydrophobic metabolites related to tea flavor quality (Li et al., 2021). Various lipid metabolites detected in our study include fatty acyl, glycerol lipids, glycerol phospholipids, and pregnenolone lipids. Lipid metabolism plays a significant role in D1 and D2, and the glycerophospholipid metabolic pathway is the most prominent. Except for methyl helianthenoate-a glucoside and neoacrimarine F, most lipid metabolites were higher in D2 than in D1 (D1 is fresh jujube leaf, D2 is jujube leaf tea). PC (16:0/18:3 (9Z, 12Z, 15Z)), PG(18:1(11Z)/18:3(9Z,12Z,15Z)), PG (20:4 (5Z,8Z,11Z,14Z)/22:6 (4Z,7Z,10Z,13Z,16Z,19Z)), and 1,2-Di-O-palmitoyl-3-O - (6-sulfoquinovopyranosyl) glycerol increased most significantly. This might be due to the high processing temperature promoting lipid transformation. Previous research identified that during green tea production, Phosphatidylcholine, Phosphatidylglycerol, and Phosphatidylethanolamine showed an increasing trend after a long high-temperature exposure (Li et al., 2021), which is consistent with our results.

##### 3.1.2.3 Amino acids and their derivatives

Amino acids play an essential role in tea soup and aroma (Yao et al., 2006) as shown in **Table 2**. In D2, L-(-)-threonine, L-phenylalanine, L-tryptophan, L-asparagine anhydrous, L-(-)-tyrosine, L-serine, L-asparticacid, L-(+)-arginine, L-alanine, L- glutamic acid increased significantly, potentially resulting from protein decomposition (Fraser et al., 2014; Liu et al., 2018) and oxidation of amino acids such as L-asparagine anhydrous, L-(+)-lysine, L-Phenylalanine, and L-asparticacid (Nathanael and Wille, 2019). The increase in tryptophan was probably due to the promotion of chorismic acid, a biosynthetic intermediate (Gibson and Gibson, 1964). This was related to the significant enrichment of the tryptophan metabolism pathway. Amino acids had flavor characteristics. The content of sweet amino acids (serine, threonine, alanine), fresh amino acids (aspartic acid, glutamic acid), and aromatic amino acids (arginine, tyrosine, phenylalanine) increased after processing into D2, while the content of bitter amino acids (isoleucine) decreased, bringing a fresh and sweet taste to jujube leaf tea (Scharbert and Hofmann, 2005; Yu et al., 2014; Dai et al., 2015; Yu and Yang, 2020).

**Table 2:** Changes in amino acid content of jujube leaves before and after processing.

Amino acid	Concentration (ng/mg)		Significance
	D1	D2	
L-Alanine	0.7595 ± 0.0468	8.4164 ± 0.6122	**
L-(+)-Arginine	10.8746 ± 0.3730	34.3396 ± 0.9493	**
L-Asparagine Anhydrous	1.5223 ± 0.0398	10.5983 ± 0.5759	**
L-AsparticAcid	3.4421 ± 0.0718	9.9193 ± 0.0355	**
L-Glutamine	0.7001 ± 0.0220	2.9210 ± 0.1266	*
L-GlutamicAcid	0.0193 ± 0.0046	8.3479 ± 0.2546	**
Glycine	0.4184 ± 0.0699	0.6455 ± 0.1350	
L-Histidine	2.8057 ± 0.1341	4.7529 ± 0.1839	*
L-Isoleucine	4.9850 ± 0.8250	3.8939 ± 0.2091	*
L-Leucine	1.2892 ± 0.0548	1.9906 ± 0.0423	
L-Hydroxyproline	0.3454 ± 0.0060	1.4673 ± 0.1389	*
L-Tryptophan	1.1112 ± 0.1065	3.2294 ± 0.0540	*
L-(+)-Lysine	1.1182 ± 0.0036	1.4048 ± 0.0163	
L-Methionine	0.0516 ± 0.0025	0.7330 ± 0.0226	
L-Phenylalanine	0.8322 ± 0.0278	3.0297 ± 0.1093	*
L-Proline	1.1221 ± 0.0162	2.9393 ± 0.0395	
L-Serine	1.3104 ± 0.0373	6.3473 ± 0.1883	**
L-(-)-Threonine	0.5849 ± 0.0494	3.8235 ± 0.4293	*
L-(-)-Tyrosine	1.7618 ± 0.0773	3.2459 ± 0.0734	*
L-Valine	0.6081 ± 0.0114	1.6812 ± 0.0311	

*P<0.05, **P<0.01.

##### 3.1.2.4 Flavonoids

Flavonoids are essential active ingredients in both fresh jujube leaves and processed tea. We found that the contents of proanthocyanidin A5’, dihydrobrinetin, 3,3’-digalloylprocyanidin B2, epiafzelechin- (4b->8) -epicatechin 3,3’-digallate and epigallocatechin 3-o- (4-hydroxybenzoate) increased in jujube leaves after tea processing. Notably, the concentration of 6-hydrohydraidzein 4’-glucoside increased fivefold after processing compared to the fresh jujube leaves due to the hydrothermal hydrolysis of flavonoid glycosides. Aglycones and glycosomes are also produced during processing. Glycoside flavonoids can significantly enhance pentobarbital-induced sleep (Wang et al., 2008). This may make jujube leaf tea have the function of helping sleep. Previous studies have shown that flavonoid aglycones are absorbed by the body relatively quickly, which enhances their functional activity.

### 3.2 Volatile metabolites analysis in jujube leaf tea before and after processing

#### 3.2.1 GC-IMS determination of volatile metabolites in jujube leaves before and after processing

**Figure 4:**
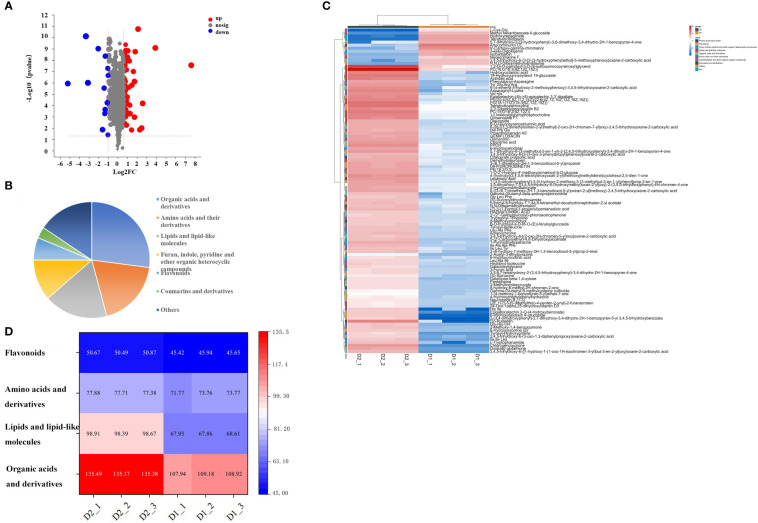
**(A)** The topographic plot of D1 and D2 based on GC-IMS, The ordinate represents the retention time of gas chromatography, and the abscissa represents the ion migration time. Each point on both sides of the rip peak(the horizontal red line) represents a volatile organic compound. The compound concentration is represented by different colors, with white corresponding to low concentration and red corresponding to high concentration. The darker the color, the greater the concentration. **(B)** The three-dimensional topographic map of D1 and D2, **(C)** Difference comparison plots of D1 and D2, **(D)** Dynamic fingerprints of jujube leaves before and after processing, generated by Gallery Plot. Each row represents the sample signal peak, while each column represents each volatile compound in the different samples. The color represents the volatile compound concentration. The brighter the color, the higher the concentration.

To identify the presence and changes in aromatic compounds in jujube leaf tea processing, we carried out GC-IMS to obtain global IMS information. The topographic map obtained from the GC-IMS analysis is shown in **Figure 4A**. The three-dimensional spectra of jujube leaf tea volatile components before and after processing are presented in **Figure 4B**. There was a significant difference between volatile organic compounds between D1 and D2. To evaluate these differences more in-depth, the difference comparison mode was adopted: the spectrum of D1 was selected as the reference, and the spectrum of D2 was deducted from the reference. Suppose two volatile organic compounds have the same concentration in D1 and D2. In that case, the background after deduction is white(**Figure 4C**). At the same time, red represents that the target compound concentration is higher than the reference, and blue is lower, respectively. As shown in **Figure 4C**, most signals appear in the retention time range of 200 to 600 seconds and the drift time range of 1.0 to 1.8 seconds. There are obvious red points representing a higher compound concentration and blue spots representing a lower concentration at this location in the D2 map. This indicates that compared with D1, the volatile compound composition of D2 has changed significantly. The dynamic changes of these volatile components form the unique flavor characteristics of jujube leaf tea.

A total of 112 peaks were observed through qualitative analysis of volatile compounds. Compared with the GC - IMS library, 74 peaks of 52 compounds were identified, including 17 esters, 11 alcohols, 10 aldehydes, 10 ketones, 2 olefins, 1 acid, and 1 sulfide (**Supplementary Materials Table S3**). Among them, 38 peaks could not be annotated. Certain volatile compounds may produce numerous signals due to high proton affinity or signals that allow ions to form dimers and trimers when moving in the drift tank, such as (z) -3-hexenyl butyrate, hexyl 2-methyl butanoate, 1-hexanol, nonanal, 1-hydro-2-propanone. The visualization of characteristic fingerprints was carried out to observe the signal difference of volatile compounds before and after processing (**Figure 4D**).

After jujube leaf processing, esters (ethyl acetate, propyl butyrate, ethyl isobutyrate, ethyl crotonate, ethyl 3-methyl butyrate, folic alcohol acetate, Etc.), partial alcohols (linalool, 3-methyl-2-butanol, ethanol, 1-hexanol, Z-3-hexen-1-alcohol, 3-methyl butanol), olefins(β- Pinene, β- Ocimene) and methyl sulfide were significantly reduced (**Figure 4D**, red box), which reduced grass and fruit flavors (Colonges et al., 2022; Li et al., 2022). Aldehyde content (glutaraldehyde, nonanal, furfural, (E)-2-hexenal, (E)-2-pentenoaldehyde, heptanaldehyde, etc.), ketones (2,3-butanedione, 2-butanone, 1-penten-3-one, hydroxyacetone, etc.), some alcohols (Z-2-pentenol, 1-penten-3-alcohol, isobutanol) and acetic acid increased significantly (**Figure 4D**, yellow box), providing the aromas of almond oil, cream, and fragrance (Kfoury et al., 2018; Huang et al., 2022). Ethyl 3-methylbutyrate and ethyl crotonate accounted for 19.32% of the volatile metabolites in D1, occupying a dominant position. (E)-2-hexenal (dimer) in D2 accounts for 11% of the volatile substances, accounting for the highest proportion, and emits a unique green leaf smell(Liu, Liu et al., 2022). It is also the main active aroma compound in jujube fruit, thus giving jujube leaf tea the aroma of the jujube fruit (Song et al., 2019).

#### 3.2.2 GC-MS determination of volatile metabolites in jujube leaves before and after processing

To study the changes in the content of volatile metabolites in jujube leaves before and after processing, and to supplement the determination of metabolites by GC-IMS, solid-phase microextraction technology was used to extract volatile substances, and the extracts were analyzed using gas chromatography-mass spectrometry. A total of 24 volatile compounds were identified by GC-MS, and it was found that there was a significant increase in Hexanal, 1-Penten-3-ol, (E) -2-Hexanal, 6-Methyl-5-hepten-2-one, and Acetic acid in D2, while there was a significant decrease in 2-ethyl-Furan, (Z) -3-Hexenyl acetate, (Z) -3-Hexen-1-ol, Oxime, methoxy-phenyl -, and Eugenol. Allyl ethyl ether, D-limonene, Benzene,4-ethyl-1,2-dimethyl-, (Z)-Hex-3-enyl2-methylbutyrate, 3,5-Octadien-2-one, Hexanoic acid, 1,3-cis,5-cis-Octatriene, these substances grew from scratch during the processing of jujube leaf tea, giving it a unique aroma.

OAV is used to evaluate the contribution of volatile compounds to the aroma of tea. It is generally believed that volatile metabolites with OAV≥1 contribute significantly to the overall aroma, while volatile metabolites with OAV≥10 are identified as important aroma components (Tan et al., 2022). From the **Supplementary Materials Table S4**, it can be concluded that there are 9 substances with OAV≥1 in D1 and 8 in D2. 2-pentyl-Furan, (E) -2-Hexanal, 1-Pentanol, 6-Methyl-5-hepten-2-one were common and important aroma components in D1 and D2. Eugenol was the volatile compound with the highest OAV value in D1, which can be considered to be the characteristic flavor substance in jujube leaves. Eugenol, as a natural compound, has various pharmacological effects, such as antioxidant, anticancer, hypoglycemic, antibacterial, etc (Pramod et al., 2010). Natural food flavor (E)-2-Hexanal is a potential antifungal compound with high content in D2 (Ma et al., 2019), which was the key flavor active substance in D2. By combining the identified volatile flavor compounds with their aroma thresholds, we further understood the specific contribution of each substance to the overall flavor formation.

### 3.3 Discussion

Extensively targeted metabonomics has been successfully applied to large-scale metabolite analysis and comparative metabonomics of many essential plant species (Mattivi et al., 2006; Wang et al., 2016; Cebulj et al., 2017; Price et al., 2020). In jujube, previous studies focused more on the metabolites of jujube fruits including the nutrient components, active components, the color in different maturity (Feng et al., 2020; Shen et al., 2022; Shi et al., 2022). Comprehensive research on LC-MS-based metabolomics, GC-IMS and GC-MS analysis has not yet to be carried out on the jujube leaves before and after tea processing. In this study, we found that there were 107 different metabolites in fresh jujube leaves and leaf tea. Therefore, this study provided insights into the changes of metabolites in jujube leaves before and after processing into tea.

The aroma and taste of tea are important attributes (Ho et al., 2015; Han et al., 2016; Chen et al., 2020). Based on the volatile metabolites in jujube leaf tea, we can predict that jujube leaf tea has the scent of soft leaves, fruit, cream and fat. Among all metabolites, (E)-2-Hexenal was the most volatile metabolite, providing a solid aroma of green leaves and fruits (Buttery et al., 1987). 2-pentyl-furan provided a roasted aroma, often presented in white tea (Lin et al., 2021). 6-Methyl-5-hepten-2-one had a fresh fruity aroma and citrus like aroma, making it an intermediate in the synthesis of linalool (Baldwin et al., 2000). In our laboratory we carried out the sensorial evaluation of the jujube tea leaf. It was concluded that the jujube leaf tea had a clear soup color, a delightful taste and frangrance of roses, oranges and tender leaves, which was consistent with our prediction.

Fresh leaves contain various ester compounds. After processing into jujube leaf tea, their contents decreased significantly, while aldehydes, ketones, and olefins increased significantly. This may be due to the high temperature during the fixation processing procedure, which leads to a large amount of volatilization of alcohols and aldehydes with low boiling points, isomerization and oxidation reaction, and the formation of high boiling point volatile compounds with special flavor (Coxon et al., 1970). The increase of ketones such as 6-methyl-5-hepten-2-one is caused by the thermal degradation of carotenoids. Volatile substances such as Hexanal, Pentanal, Nonanal, 1-Penten-3-ol, (E) - 2-Hexanal, (E) - 2-Hexen-1-ol are formed with the assistance of lipoxygenase. Lipids are oxidized by lipoxygenase to form lipid hydroperoxide, which is then cracked by hydroperoxide lyase to form six carbon aliphatic aromatic compounds, such as (Z) - 3-hexenal and n-hexanal. Subsequently, these aldehydes can be further reduced to alcohols directly through alcohol dehydrogenase, or isomerized to trans isomers, and then reduced to alcohols (Ho et al., 2015).The Strecker synthesis of amino acids, glucosides, and glycosides also generates alcohols, ketones, aldehydes, alkenes, and aromatic hydrocarbons at high temperatures (Ho et al., 2015; Feng et al., 2019, Li C. et al., 2019, Li P. et al., 2019). It has been previously shown that drying can promote the glycerol phospholipids conversion to unsaturated fatty acids (Wang et al., 2022). These eventually form the unique volatile compound profile of jujube leaf green tea. In other words, jujube leaf processing can modify the amino acids and lipids content affecting the flavor and intensity of jujube leaf tea. Therefore, temperature, rolling, and other vital parameters affect the degree of the formation/transformation of non-volatile compounds in jujube leaves. These can be adjusted and fine-tuned to obtain jujube leaf tea products with different flavor characteristics.

It is reported that flavonoids have the effect of sedation and hypnosis (Jiang et al., 2007; San et al., 2013; Shi et al., 2016). There are many kinds of flavonoids in jujube leaves. After processing into jujube leaf tea, the content of flavonoids increased. Flavanol is one of the most typical and plentiful metabolites of tea (Scharbert and Hofmann, 2005). After processing fresh leaves into leaf tea, the content of Epicatechin Ent - (4alpha ->6) - ent Epicatechin, (+/-) - Catechin, and Epigallocatechin augmented. In this study, no exciting chemical components, such as caffeine and ephedrine, were found in jujube leaf tea. As flavonoids have sedating and hypnotic effects, we predict that jujube leaf tea will have hypnotic effects. This necessitates further study.

## 4 Conclusion

Jujube leaf tea metabolomic analysis combined with GC-IMS and GC-MS showed that jujube leaves’ non-volatile and volatile metabolites changed significantly before and after processing. A total of 468 non-volatile were identified, and 109 non-volatile metabolites were differentially accumulated and further evaluated. We found that the rapid transformation of lipids, organic acids, amino acids, and flavonoids was the main factor regulating the jujube leaf tea sensory quality. The pathway enrichment analysis revealed that glycerophospholipid metabolism, tryptophan metabolism, and linoleic acid metabolism had the most significant impact. 52 and 24 volatile metabolites were identified by GC-IMS and GC-MS, respectively. Eugenol was the main flavor substances in fresh jujube leaves, and (E) - 2-Hexenal was the fundamental volatile substance of jujube leaf tea. Besides the changes in volatile metabolites, amino acids and lipids play an essential role in aromatic compound formation. This study provides novel insights into metabolic profile changes before and after jujube leaf processing and how they affect its sensory and health-promoting properties. It provides technical insights for improving the quality and efficiency of the jujube processing industry.

## Data availability statement

The original contributions presented in the study are included in the article/**Supplementary Material**. Further inquiries can be directed to the corresponding authors.

## Author contributions

All authors listed have made a substantial, direct, and intellectual contribution to the work and approved it for publication.
