# Supplementary material for: Combined LC-MS-based metabolomics and GC-IMS analysis reveal changes in chemical components and aroma components of Jujube leaf tea during processing
Source: Front Plant Sci. 2023 May 17;14:1179553. doi: 10.3389/fpls.2023.1179553 (PMC10231682; doi:10.3389/fpls.2023.1179553)
Supplement: Supplementary file 1 [file Table_1.docx]

**Combined LC-MS-based metabolomics and GC-IMS analysis reveal changes in chemical components and aroma components of Jujube leaf tea during processing**

**Tables**

**Table S1**. List of 468 nonvolatile metabolites before and after jujube leaf processing

| No. | Metabolite | Formula | Adducts | M/Z | Retention time | Library ID | D1_1 | D1_2 | D1_3 | D2_1 | D2_2 | D2_3 |
| --- | --- | --- | --- | --- | --- | --- | --- | --- | --- | --- | --- | --- |
| 1 | Harmalol | C12H12N2O | M+H | 201.1021 | 4.8488 | HMDB0029838 | 5.0829 | 5.1491 | 5.1326 | 4.42 | 4.5565 | 4.5848 |
| 2 | Pseudoecgonine | C9H15NO3 | M+H-H2O | 168.1018 | 6.2593 | HMDB0006348 | 5.2004 | 5.2439 | 5.2482 | 4.3358 | 4.4018 | 4.376 |
| 3 | Heliotrine | C16H27NO5 | M+H | 314.1956 | 4.9742 | HMDB0253072 | 5.6693 | 5.6642 | 5.6512 | 3.014 | 2.9976 | 3.0034 |
| 4 | Erysothiopine | C19H21NO7S | M+NH4 | 425.1413 | 3.7446 | HMDB0030257 | 4.8648 | 4.8675 | 4.9191 | 6.1938 | 6.2297 | 6.197 |
| 5 | NPC | C28H30N4O6 | M+FA-H | 563.2133 | 5.5911 | HMDB0060499 | 5.0365 | 5.0096 | 5.0671 | 5.6537 | 5.6378 | 5.7077 |
| 6 | Vanilloloside | C14H20O8 | M+NH4, M+Na | 334.1493 | 3.0432 | HMDB0032013 | 4.5046 | 4.5706 | 4.5354 | 5.3645 | 5.3441 | 5.3179 |
| 7 | Beta-D-Glucopyranosyl anthranilate | C13H17NO7 | M+CH3OH+H, 2M+K | 637.1737 | 3.5696 | HMDB0041517 | 6.1023 | 6.1359 | 6.0834 | 5.4824 | 5.4968 | 5.4866 |
| 8 | Sucrose | C12H22O11 | M+Na | 365.1051 | 2.2581 | HMDB0000258 | 5.5151 | 5.5036 | 5.5125 | 6.7508 | 6.7529 | 6.7627 |
| 9 | Benzoyl glucuronide (Benzoic acid) | C13H14O8 | M+CH3OH+H | 331.102 | 3.4779 | HMDB0010324 | 2.9692 | 2.996 | 2.9942 | 4.1199 | 4.0644 | 4.0819 |
| 10 | Cis-Ferulic acid [arabinosyl-(1->3)-[glucosyl-(1->6)]-glucosyl] ester | C27H38O18 | M+H-2H2O | 615.1916 | 3.5556 | HMDB0034682 | 5.991 | 5.9896 | 5.9565 | 4.9954 | 5.0213 | 4.9815 |
| 11 | 3-O-p-Coumaroylquinic acid | C16H18O8 | M+2Na-H | 383.0708 | 4.468 | HMDB0029681 | 4.8849 | 4.8391 | 4.7781 | 4.3081 | 4.2286 | 4.1315 |
| 12 | Vomifoliol 9-[glucosyl-(1->4)-xylosyl-(1->6)-glucoside] | C30H48O17 | M+H | 681.2938 | 4.9882 | HMDB0038449 | 5.8231 | 5.8639 | 5.8715 | 5.0167 | 5.0004 | 5.0061 |
| 13 | 1-(5-Methyl-3-pyridinyl)-1-decanone | C16H25NO | M+H-H2O | 230.1902 | 5.9508 | HMDB0031952 | 5.5728 | 5.6291 | 5.6237 | 4.9542 | 4.9444 | 4.9333 |
| 14 | 3-Ethyl-1,2-cyclopentanedione | C7H10O2 | M+ACN+H | 168.1018 | 6.1405 | HMDB0031222 | 5.0385 | 5.0492 | 5.0443 | 4.2763 | 4.2684 | 4.1784 |
| 15 | Alpha-Trisaccharide | C20H37NO14 | M+NH4 | 533.2588 | 6.2243 | HMDB0006595 | 3.9104 | 3.7952 | 3.7935 | 5.0947 | 5.1246 | 5.1203 |
| 16 | 4-Hydroxy-5-(phenyl)-valeric acid-O-glucuronide | C17H22O9 | M+NH4 | 388.1596 | 5.2058 | HMDB0059980 | 4.7359 | 4.8095 | 4.8598 | 5.7125 | 5.7154 | 5.7217 |
| 17 | Rishitin | C14H22O2 | M+NH4 | 240.1956 | 3.9833 | HMDB0302980 | 5.2063 | 5.2538 | 5.2337 | 3.0068 | 2.8895 | 3.3197 |
| 18 | Isokobusone | C14H22O2 | M+NH4 | 240.1956 | 3.8223 | HMDB0036791 | 5.3474 | 5.3287 | 5.3335 | 3.3171 | 3.6067 | 3.3065 |
| 19 | Galactose-beta-1,4-xylose | C11H20O10 | M+H-H2O | 295.1022 | 3.7376 | HMDB0011677 | 2.4403 | 2.467 | 2.7307 | 4.5914 | 4.5791 | 4.5967 |
| 20 | Dhelwangin | C12H16O4 | M+ACN+H | 266.1384 | 3.7235 | HMDB0030703 | 4.6155 | 4.6722 | 4.7293 | 3.6351 | 3.7379 | 3.7926 |
| 21 | 2-Acetyl-3-ethylpyrazine | C8H10N2O | M+H | 151.0866 | 3.1699 | HMDB0029410 | 2.3225 | 2.3492 | 2.3475 | 3.7748 | 3.7921 | 3.7585 |
| 22 | N-Acetyl-4-O-acetylneuraminic acid | C13H21NO10 | M+ACN+H | 393.1499 | 2.7128 | HMDB0000796 | 5.7059 | 5.7042 | 5.7 | 3.8839 | 3.8772 | 3.9904 |
| 23 | N-Acetylmannosamine | C8H15NO6 | M+H-2H2O | 186.0761 | 2.6218 | HMDB0001129 | 5.4827 | 5.4804 | 5.4882 | 4.791 | 4.8271 | 4.7968 |
| 24 | Chitin | C16H28N2O11.  [C8H13NO5]n | M+Na | 650.2439 | 3.0502 | HMDB0248228 | 5.5129 | 5.5229 | 5.5311 | 3.2702 | 3.7792 | 3.2596 |
| 25 | Trans-p-Coumaric acid 4-glucoside | C15H18O8 | M-H, M+FA-H, M+Cl | 325.0928 | 3.8318 | HMDB0039509 | 6.2336 | 6.23 | 6.2199 | 7.5099 | 7.4908 | 7.4639 |
| 26 | Caffeic acid 3-O-glucuronide | C15H16O10 | M-H, M+Na-2H | 355.067 | 3.175 | HMDB0041705 | 6.9722 | 6.8539 | 6.9815 | 5.9375 | 5.8529 | 5.936 |
| 27 | Chlorogenoquinone | C16H16O9 | M-H, M+Na-2H | 373.0538 | 3.3694 | HMDB0029383 | 1.3293 | 1.3288 | 1.3415 | 5.7338 | 5.7299 | 5.7433 |
| 28 | NNAL-N-glucuronide | C16H24N3O8+ | M-H2O-H, M-H | 385.1505 | 6.2235 | HMDB0060498 | 5.3179 | 5.2928 | 5.2962 | 4.1928 | 4.1446 | 4.1469 |
| 29 | 1-(beta-D-Ribofuranosyl)-1,4-dihydronicotinamide | C11H16N2O5 | M+Na-2H | 277.0828 | 3.0909 | HMDB0011643 | 6.0644 | 5.956 | 6.0768 | 3.7458 | 3.6491 | 3.8381 |
| 30 | D-Glucarate | C6H10O8 | M-H | 209.0298 | 3.559 | HMDB0000663 | 5.0627 | 5.1325 | 5.0505 | 4.2654 | 4.3108 | 4.2587 |
| 31 | 4-Hydroxy-2,6,6-trimethyl-3-oxo-1,4-cyclohexadiene-1-carboxaldehyde | C10H12O3 | M+FA-H | 225.0765 | 4.8398 | HMDB0037602 | 5.0539 | 5.0729 | 5.0289 | 2.6156 | 2.6114 | 2.6037 |
| 32 | 3-Ethyl-2-hydroxy-4-methyl-2-cyclopenten-1-one | C8H12O2 | M-H | 139.0754 | 4.8845 | HMDB0036176 | 4.1843 | 4.2212 | 4.1362 | 2.6819 | 3.2744 | 3.2222 |
| 33 | PHLORACETOPHENONE | C8H8O4 | M-H | 167.0341 | 5.3962 | HMDB0029644 | 6.7659 | 6.7521 | 6.7836 | 5.786 | 5.7924 | 5.8153 |
| 34 | 4-Ipomeanol | C9H12O3 | M-H | 167.0705 | 4.8815 | HMDB0030472 | 4.7388 | 4.7138 | 4.6942 | 2.3639 | 2.6284 | 2.7136 |
| 35 | Isopropyl beta-D-glucoside | C9H18O6 | M-H2O-H | 203.0918 | 4.8599 | HMDB0032705 | 3.3441 | 3.3029 | 3.5269 | 4.6283 | 4.653 | 4.7321 |
| 36 | 2,5-Dihydroxybenzaldehyde | C7H6O3 | M-H | 137.0234 | 2.9547 | HMDB0004062 | 4.8681 | 4.8732 | 4.8428 | 5.6606 | 5.5916 | 5.6871 |
| 37 | Gynocardin | C12H17NO8 | M-H2O-H | 284.0776 | 2.8509 | HMDB0029913 | 4.1904 | 4.2186 | 4.2348 | 5.7972 | 5.774 | 5.8064 |
| 38 | Dihydromaleimide beta-D-glucoside | C10H15NO7 | M+FA-H | 306.083 | 2.8441 | HMDB0030277 | 4.6421 | 4.581 | 4.5717 | 5.4246 | 5.3789 | 5.4388 |
| 39 | 2-Methoxy-1,4-benzoquinone | C7H6O3 | M+FA-H | 183.0292 | 2.7159 | HMDB0032576 | 2.4358 | 2.4785 | 2.4485 | 5.0361 | 5.056 | 5.0601 |
| 40 | 6-Hydroxymelatonin glucuronide | C19H24N2O9 | M+Na-2H | 445.1197 | 1.0031 | HMDB0060786 | 6.0047 | 5.9671 | 6.0395 | 7.5484 | 7.555 | 7.5546 |
| 41 | Fucosyllactose | C18H32O15 | M+FA-H | 533.1722 | 0.9894 | HMDB0006620 | 6.1386 | 6.1124 | 6.1361 | 7.2728 | 7.2778 | 7.2756 |
| 42 | Gluconic acid | C6H12O7 | M-H | 195.0504 | 0.9347 | HMDB0000625 | 6.5013 | 6.4781 | 6.5069 | 5.6655 | 5.6287 | 5.6511 |
| 43 | N-Acetylneuraminic acid | C11H19NO9 | M-H2O-H | 290.0882 | 1.7853 | HMDB0000773 | 3.9968 | 4.0951 | 4.2208 | 4.9587 | 4.9916 | 4.9622 |
| 44 | Hypoglycin | C7H11NO2 | M+H-H2O, M+H | 142.0863 | 2.9228 | HMDB0029427 | 6.2687 | 6.2813 | 6.2939 | 5.5606 | 5.6469 | 5.7036 |
| 45 | N-lactoyl-phenylalanine | C12H15NO4 | M+H, 2M+ACN+Na | 238.1072 | 3.1489 | HMDB0062175 | 3.9167 | 3.8092 | 3.8704 | 4.9623 | 4.9528 | 4.9445 |
| 46 | Indolylacryloylglycine | C13H12N2O3 | M+H-H2O, M+H | 245.0919 | 4.8558 | HMDB0006005 | 6.917 | 6.9685 | 6.9679 | 6.3523 | 6.3881 | 6.3747 |
| 47 | 3-Hydroxydodecanedioic acid | C12H22O5 | M+H-2H2O, M+Na | 211.1327 | 5.2337 | HMDB0000413 | 5.886 | 5.9388 | 5.9262 | 4.3819 | 4.4105 | 4.3534 |
| 48 | Tryptophyl-Threonine | C15H19N3O4 | M+NH4, M+ACN+Na | 323.1709 | 3.1769 | HMDB0029093 | 3.2061 | 3.2328 | 3.2311 | 4.9157 | 4.9633 | 4.9372 |
| 49 | Daucic acid | C7H8O7 | M+ACN+H | 246.0606 | 1.158 | HMDB0031665 | 7.4765 | 7.4861 | 7.4779 | 6.6914 | 6.7082 | 6.6967 |
| 50 | Hawkinsin | C11H17NO6S | M+H-2H2O | 256.0661 | 1.4688 | HMDB0002354 | 6.2178 | 6.2375 | 6.2348 | 4.9361 | 4.9583 | 4.9666 |
| 51 | Asymmetric dimethylarginine | C8H18N4O2 | M+H-H2O | 185.1397 | 2.5798 | HMDB0001539 | 6.5264 | 6.5728 | 6.5853 | 4.8797 | 4.8913 | 4.902 |
| 52 | Saccharopine | C11H20N2O6 | M+H | 277.1399 | 2.6706 | HMDB0000279 | 5.1997 | 5.1687 | 5.1718 | 4.5445 | 4.5769 | 4.5606 |
| 53 | Phenylalanyl-Asparagine | C13H17N3O4 | M+NH4 | 297.1554 | 2.9935 | HMDB0028990 | 2.3936 | 2.6164 | 2.6371 | 5.6851 | 5.6615 | 5.639 |
| 54 | 4-Amino-4-deoxychorismic acid | C10H11NO5 | M+H | 226.0708 | 3.0782 | HMDB0304170 | 5.1622 | 5.2182 | 5.2237 | 4.5331 | 4.5345 | 4.5237 |
| 55 | Neuromedin B (1-3) | C12H22N4O5 | M+H-2H2O | 267.1448 | 3.1629 | HMDB0013016 | 2.7895 | 2.8678 | 2.924 | 4.5501 | 4.5285 | 4.5465 |
| 56 | Gamma-Glutamyl-S-methylcysteine sulfoxide | C9H16N2O6S | M+H-2H2O | 245.0589 | 3.1699 | HMDB0031863 | 2.7278 | 2.7546 | 2.8513 | 4.2563 | 4.2282 | 4.1602 |
| 57 | Asparaginyl-Lysine | C10H20N4O4 | M+H-2H2O | 225.1345 | 3.8643 | HMDB0028736 | 1.8763 | 3.0824 | 2.7047 | 6.1407 | 6.1542 | 6.1715 |
| 58 | Captopril-cysteine disulfide | C12H20N2O5S2 | M+H-H2O | 319.0806 | 4.054 | HMDB0060562 | 4.0417 | 4.0685 | 4.0668 | 5.1837 | 5.2238 | 5.1997 |
| 59 | N(6)-(Octanoyl)lysine | C14H28N2O3 | M+Na | 295.2014 | 4.1666 | HMDB0011684 | 4.8753 | 4.9033 | 4.8912 | 3.5289 | 3.5125 | 3.5183 |
| 60 | Frangulanine | C28H44N4O4 | M+H | 501.343 | 6.1055 | HMDB0030199 | 6.5329 | 6.5511 | 6.5552 | 7.2053 | 7.1948 | 7.1799 |
| 61 | Phenylbutyrylglutamine | C15H20N2O4 | M+H-H2O | 275.1387 | 6.2383 | HMDB0011687 | 5.9226 | 5.9357 | 5.9368 | 5.2121 | 5.1591 | 5.2177 |
| 62 | Lysyl-Arginine | C12H26N6O3 | 2M+ACN+H | 646.4517 | 6.6153 | HMDB0028945 | 5.7969 | 5.7975 | 5.8075 | 3.1249 | 3.1085 | 3.1142 |
| 63 | N(6)-Methyllysine | C7H16N2O2 | M+H-H2O | 143.1178 | 8.1659 | HMDB0002038 | 4.3466 | 4.3918 | 4.2992 | 3.6969 | 3.6813 | 3.7109 |
| 64 | BICINE | C6H13NO4 | M+H | 164.0916 | 8.7319 | HMDB0011727 | 4.9673 | 4.9745 | 4.9396 | 4.3216 | 4.2881 | 4.2144 |
| 65 | 12-Hydroxydodecanoic acid | C12H24O3 | M+Na | 239.1612 | 7.2798 | HMDB0304547 | 5.2966 | 5.4098 | 5.3721 | 4.613 | 4.646 | 4.6462 |
| 66 | Oxidized glutathione | C20H32N6O12S2 | M+ACN+H | 654.1888 | 6.9014 | HMDB0011691 | 1.646 | 1.6722 | 1.6705 | 5.6644 | 5.472 | 5.4284 |
| 67 | Ethenyl acetate | C4H6O2 | M+K | 124.9999 | 6.6921 | HMDB0031209 | 2.7959 | 2.8226 | 2.8209 | 4.1437 | 4.2068 | 4.1729 |
| 68 | Mauritine A | C32H41N5O5 | M+H-H2O | 558.3069 | 5.9011 | HMDB0029338 | 4.9499 | 4.9702 | 4.9521 | 5.976 | 5.9396 | 5.931 |
| 69 | Tyrosyl-Isoleucine | C15H22N2O4 | M+H-2H2O | 259.1438 | 5.1709 | HMDB0029108 | 2.8695 | 3.2499 | 3.4062 | 5.0793 | 5.1199 | 5.0701 |
| 70 | 5-Hydroxyindoleacetylglycine | C12H12N2O4 | M+H-H2O | 231.0762 | 4.8412 | HMDB0004185 | 5.2632 | 5.31 | 5.3175 | 4.667 | 4.6835 | 4.5961 |
| 71 | S-Lactoylglutathione | C13H21N3O8S | M+H-H2O | 362.0997 | 4.2776 | HMDB0001066 | 5.6776 | 5.7126 | 5.7233 | 2.884 | 2.8909 | 2.8799 |
| 72 | Glutaminylarginine | C11H22N6O4 | M+H-2H2O | 267.1588 | 4.2354 | HMDB0028791 | 5.0733 | 5.0695 | 5.0905 | 4.0205 | 4.0387 | 4.0349 |
| 73 | (+/-)-Metalaxyl | C15H21NO4 | M+CH3OH+H | 312.1802 | 4.068 | HMDB0031802 | 5.0438 | 5.0866 | 5.1076 | 4.3479 | 4.3315 | 4.3373 |
| 74 | Tetrahydrodipicolinate | C7H9NO4 | M+H-H2O | 154.0498 | 3.7873 | HMDB0012289 | 3.8277 | 3.8611 | 3.8699 | 4.7809 | 4.7393 | 4.738 |
| 75 | (S)-5-Amino-3-oxohexanoate | C6H11NO3 | 2M+ACN+H | 332.1812 | 3.289 | HMDB0012131 | 4.0157 | 4.0425 | 4.0408 | 4.6882 | 4.7107 | 4.7863 |
| 76 | Deoxypyridinoline | C18H28N4O7 | M+H-2H2O | 377.1815 | 3.2125 | HMDB0000569 | 5.6127 | 5.6119 | 5.657 | 3.9674 | 3.7272 | 3.9434 |
| 77 | Glycyl-Tryptophan | C13H15N3O3 | M+2Na-H | 306.0844 | 3.0922 | HMDB0028852 | 5.2199 | 5.2583 | 5.2481 | 2.7809 | 2.7645 | 2.7703 |
| 78 | Histidinyl-Isoleucine | C12H20N4O3 | M+H | 269.1605 | 2.9228 | HMDB0028888 | 2.9604 | 2.9872 | 2.9855 | 4.8835 | 4.8588 | 4.8812 |
| 79 | Threoninyl-Proline | C9H16N2O4 | M+H-H2O | 199.1077 | 2.8458 | HMDB0029069 | 4.1535 | 4.2809 | 4.2677 | 5.0147 | 5.0571 | 5.0309 |
| 80 | Gamma-Glutamyl-beta-aminopropiononitrile | C8H13N3O3 | M+H-H2O | 182.0924 | 2.8458 | HMDB0060477 | 1.9262 | 3.3216 | 3.1566 | 5.1431 | 5.1747 | 5.169 |
| 81 | Dopaquinone | C9H9NO4 | M+ACN+H | 237.0868 | 2.6357 | HMDB0039119;  HMDB0001229 | 5.2507 | 5.1877 | 5.1942 | 3.8013 | 3.7759 | 3.7391 |
| 82 | 3-Dehydroquinate | C7H10O6 | M+H | 191.055 | 2.3418 | HMDB0012710 | 5.6114 | 5.6373 | 5.6458 | 4.482 | 4.5002 | 4.5506 |
| 83 | N2-Succinyl-L-ornithine | C9H16N2O5 | M+H | 233.113 | 1.9228 | HMDB0001199 | 6.6087 | 6.5602 | 6.5474 | 5.6618 | 5.6738 | 5.6585 |
| 84 | Ascorbalamic acid | C9H13NO8 | M+H-2H2O | 228.0501 | 1.158 | HMDB0029944 | 6.2191 | 6.2137 | 6.1742 | 4.9576 | 5.0056 | 4.8787 |
| 85 | Gamma-delta-Dioxovaleric acid | C5H6O4 | M+FA-H, 2M+FA-H | 175.0241 | 2.8441 | HMDB0013233 | 5.1848 | 5.1211 | 5.1225 | 5.8278 | 5.8168 | 5.8305 |
| 86 | 4-(Glutamylamino) butanoate | C9H16N2O5 | M-H, M+Na-2H | 231.0983 | 1.885 | HMDB0012161 | 6.5038 | 6.4996 | 6.5008 | 5.5143 | 5.5243 | 5.5273 |
| 87 | N-Lauroylglycine | C14H27NO3 | M-H, M+Cl | 256.1917 | 6.6269 | HMDB0013272 | 5.1971 | 5.2448 | 5.208 | 6.3173 | 6.2425 | 6.227 |
| 88 | Myristoylglycine | C16H31NO3 | M-H, M+Cl | 284.223 | 6.7432 | HMDB0013250 | 4.8258 | 4.7739 | 4.761 | 5.7031 | 5.6487 | 5.6313 |
| 89 | L-Aspartic Acid | C4H7NO4 | M-H | 132.0292 | 0.9278 | HMDB0000191 | 6.44 | 6.4442 | 6.4583 | 5.3014 | 5.3069 | 5.2964 |
| 90 | Serylisoleucine | C9H18N2O4 | M-H | 217.1189 | 2.9888 | HMDB0029042 | 4.9885 | 4.9779 | 4.9633 | 4.2368 | 4.3353 | 4.3273 |
| 91 | Chorismic acid | C10H10O6 | M-H | 225.0401 | 3.0025 | HMDB0012199 | 3.4122 | 3.2709 | 3.2843 | 5.7264 | 5.7438 | 5.7487 |
| 92 | DL-o-Tyrosine | C9H11NO3 | M-H | 180.0659 | 3.084 | HMDB0006050 | 5.0824 | 4.9754 | 5.0958 | 4.4423 | 4.352 | 4.4533 |
| 93 | Acetyl-L-tyrosine | C11H13NO4 | M-H | 222.0768 | 3.3763 | HMDB0000866 | 5.0568 | 4.9548 | 4.9884 | 4.408 | 4.2621 | 4.3079 |
| 94 | Aspartyl-Tyrosine | C13H16N2O6 | M-H2O-H | 277.0828 | 3.8382 | HMDB0028765 | 5.1882 | 5.1811 | 5.1391 | 3.6472 | 3.7629 | 3.4651 |
| 95 | 4-Hydroxy-2-oxoglutaric acid | C5H6O6 | M-H2O-H | 142.9977 | 5.2239 | HMDB0002070 | 3.794 | 3.7379 | 3.8838 | 2.7592 | 2.755 | 3.1874 |
| 96 | (S)-Spinacine | C7H9N3O2 | M-H2O-H | 148.052 | 6.0366 | HMDB0029873 | 3.0443 | 2.4545 | 2.4678 | 4.4738 | 4.5198 | 4.5117 |
| 97 | N-Gluconyl ethanolamine phosphate | C8H18NO10P | M+Cl | 354.0386 | 6.1334 | HMDB0032294 | 4.0865 | 4.0859 | 4.0993 | 5.199 | 5.3239 | 5.3459 |
| 98 | Aspergillomarasmine A | C10H17N3O8 | 2M+Hac-H | 673.2159 | 6.5456 | HMDB0029450 | 4.3201 | 4.5157 | 4.2311 | 5.0295 | 4.988 | 5.0323 |
| 99 | Aspartyl-Tryptophan | C15H17N3O5 | 2M-H | 637.2306 | 6.7363 | HMDB0028764 | 3.8692 | 3.8687 | 3.882 | 5.5085 | 5.4628 | 5.4298 |
| 100 | Oleoyl glycine | C20H37NO3 | M+FA-H | 384.2754 | 6.8076 | HMDB0013631 | 3.1226 | 3.122 | 3.1354 | 4.8606 | 4.568 | 4.587 |
| 101 | N-Decanoylglycine | C12H23NO3 | M-H | 228.1602 | 6.5037 | HMDB0013267 | 3.8468 | 3.8824 | 3.9953 | 5.0764 | 5.0948 | 5.0789 |
| 102 | Lysyl-Hydroxyproline | C11H21N3O4 | 2M-H | 517.301 | 6.4204 | HMDB0028952 | 5.2527 | 5.3269 | 5.2616 | 4.682 | 4.6386 | 4.5794 |
| 103 | Capryloylglycine | C10H19NO3 | M-H | 200.1286 | 6.3275 | HMDB0000832 | 3.3813 | 3.3808 | 3.3942 | 4.5348 | 4.4901 | 4.4737 |
| 104 | Rigin | C18H32N8O6 | M+Cl | 491.2131 | 6.2097 | HMDB0005771 | 5.2704 | 5.2538 | 5.2994 | 5.9039 | 5.9409 | 5.9063 |
| 105 | Pantetheine | C11H22N2O4S | M-H | 277.1226 | 4.0625 | HMDB0003426 | 2.74 | 2.7395 | 2.7528 | 4.6785 | 4.6653 | 4.7173 |
| 106 | Prolyl-Threonine | C9H16N2O4 | 2M-H | 431.2144 | 3.2081 | HMDB0029027 | 3.9929 | 3.4585 | 3.8452 | 4.8059 | 4.7555 | 4.7663 |
| 107 | Tyrosyl-Hydroxyproline | C14H18N2O5 | M-H2O-H | 275.1037 | 3.1825 | HMDB0029106 | 1.5206 | 1.5201 | 1.5724 | 4.7235 | 4.6594 | 4.7688 |
| 108 | L-Cys-Gly | C5H10N2O3S | M-H | 177.0333 | 2.8441 | HMDB0000078 | 4.6596 | 4.6166 | 4.5964 | 0.0011 | 0.3063 | 0.0691 |
| 109 | Gamma-Glutamylproline | C10H16N2O5 | M-H | 243.0984 | 2.8372 | HMDB0029157 | 5.2387 | 5.2021 | 5.2239 | 4.6157 | 4.5951 | 4.614 |
| 110 | L-alpha-Aspartyl-L-hydroxyproline | C9H14N2O6 | M-H | 245.0776 | 1.871 | HMDB0011160 | 6.0841 | 6.0916 | 6.0896 | 4.9835 | 4.9805 | 5.0026 |
| 111 | Isocitrate | C6H8O7 | M-H | 191.0191 | 1.627 | HMDB0001874 | 7.1776 | 7.1926 | 7.1861 | 6.3819 | 6.4376 | 6.4207 |
| 112 | Zymonic acid | C6H6O5 | M+NH4, M+CH3OH+H | 191.055 | 2.0766 | HMDB0031210 | 5.5115 | 5.5043 | 5.5092 | 4.662 | 4.684 | 4.6695 |
| 113 | Ethyl maltol | C7H8O3 | M+H-H2O, M+H | 123.0443 | 3.9063 | HMDB0031735 | 4.4554 | 4.5532 | 4.5096 | 5.3726 | 5.3768 | 5.3832 |
| 114 | (3b,6b,8a,12a)-8,12-Epoxy-7(11)-eremophilene-6,8,12-trimethoxy-3-ol | C18H30O5 | M+NH4, M+Na | 344.2428 | 6.3435 | HMDB0031963 | 6.5617 | 6.5691 | 6.6 | 4.7596 | 4.7359 | 4.8103 |
| 115 | Xi-2,3-Dihydro-3,5-dihydroxy-6-methyl-4H-pyran-4-one | C7H10O4 | M+H-2H2O, M+Na | 181.0495 | 3.3653 | HMDB0036380 | 4.0625 | 4.1135 | 4.089 | 5.1796 | 5.096 | 5.1631 |
| 116 | Arenaine | C11H17N3O | M+NH4, M+2Na-H | 252.1076 | 3.0362 | HMDB0030354 | 5.6735 | 5.6953 | 5.694 | 4.8013 | 4.7816 | 4.7765 |
| 117 | Phenmetrazine | C11H15NO | M+H | 178.1226 | 2.9305 | HMDB0014968 | 6.5391 | 6.5338 | 6.5484 | 3.8035 | 3.7706 | 3.6216 |
| 118 | Tetrahydrobiopterin | C9H15N5O3 | 2M+NH4 | 500.2698 | 4.2987 | HMDB0000027 | 5.5961 | 5.6122 | 5.627 | 0.5658 | 0.5539 | 0.5581 |
| 119 | N-Acetylserotonin | C12H14N2O2 | M+H-H2O | 201.1021 | 4.9952 | HMDB0001238 | 3.9845 | 3.901 | 4.0744 | 5.097 | 5.0833 | 5.0954 |
| 120 | Biliverdin | C33H34N4O6 | M+H-H2O | 565.2441 | 6.8945 | HMDB0001008 | 3.6007 | 3.8379 | 3.855 | 5.2099 | 5.0591 | 5.0072 |
| 121 | 5-Acetoxydihydrotheaespirane | C15H26O3 | M+CH3OH+H | 287.2212 | 8.2889 | HMDB0040276 | 4.8867 | 4.7654 | 4.8961 | 4.2415 | 4.0948 | 4.209 |
| 122 | 2-Hydroxypyridine | C5H5NO | M+NH4 | 113.0712 | 8.7179 | HMDB0013751 | 5.8467 | 5.8677 | 5.886 | 5.0966 | 5.0867 | 5.0884 |
| 123 | Phenobarbital | C12H12N2O3 | M+H-H2O | 215.0814 | 8.7389 | HMDB0015305 | 5.0438 | 5.028 | 5.0809 | 4.1655 | 4.2032 | 4.2546 |
| 124 | 3-keto-Digoxigenin | C23H32O5 | M+CH3OH+H | 421.2577 | 6.1753 | HMDB0060746 | 5.487 | 5.5325 | 5.4618 | 2.6939 | 2.662 | 2.6316 |
| 125 | (2S,3R)-2-[(1R)-1-[3,5-bis(trifluoromethyl)phenyl]ethoxy]-3-(4-fluorophenyl)morpholine | C20H18F7NO2 | M+2Na-H | 482.0908 | 6.0845 | HMDB0061012 | 3.884 | 3.9108 | 4.25 | 5.6946 | 5.7113 | 5.7022 |
| 126 | Austdiol | C12H12O5 | 2M+Na | 495.1258 | 5.7675 | HMDB0030858 | 3.8833 | 4.0557 | 4.0856 | 5.0929 | 5.1361 | 5.1536 |
| 127 | Hydroxyrepaglinide | C27H36N2O5 | M+2Na-H | 513.2341 | 5.7528 | HMDB0060979 | 5.6294 | 5.6452 | 5.6398 | 0.5552 | 0.8537 | 0.5475 |
| 128 | 5-Heptyltetrahydro-2-oxo-3-furancarboxylic acid | C12H20O4 | M+ACN+Na | 292.154 | 5.1779 | HMDB0030994 | 4.9208 | 4.9564 | 4.9119 | 4.1953 | 4.1789 | 4.1846 |
| 129 | 3-Methylene-indolenine | C9H7N | M+H | 130.0652 | 4.8488 | HMDB0011664 | 5.9182 | 5.9775 | 5.9697 | 5.3584 | 5.3935 | 5.3919 |
| 130 | Isoeugenitin | C12H12O4 | M+NH4 | 238.1073 | 4.6017 | HMDB0029473 | 5.1977 | 5.2326 | 5.2536 | 4.0107 | 3.9869 | 3.9336 |
| 131 | 7-Hydroxytrichodermol | C15H22O4 | M+NH4 | 284.1853 | 4.468 | HMDB0038476 | 4.9443 | 4.8937 | 4.8679 | 4.256 | 4.2396 | 4.2454 |
| 132 | Ginkgotoxin | C9H13NO3 | M+H | 184.0968 | 4.0043 | HMDB0029455 | 4.0531 | 4.0295 | 4.0807 | 4.6912 | 4.7279 | 4.7277 |
| 133 | Trp-P-1 | C13H13N3 | M+H | 212.1181 | 3.9693 | HMDB0029746 | 2.7673 | 3.1128 | 2.7923 | 3.6442 | 3.6673 | 3.782 |
| 134 | GEMIFLOXACIN | C18H20FN5O4 | M+H | 390.1544 | 3.5206 | HMDB0015286 | 3.4163 | 3.1613 | 3.1595 | 5.5539 | 5.5135 | 5.5442 |
| 135 | 1-Pyrroline | C4H7N | M+H | 70.06577 | 3.4072 | HMDB0012497 | 3.7811 | 3.7709 | 3.6935 | 2.9129 | 3.0018 | 3.0567 |
| 136 | L-Tryptophanamide | C11H13N3O | M+H | 204.1131 | 3.2195 | HMDB0013318 | 2.7086 | 0.3362 | 2.601 | 5.0628 | 5.0623 | 5.0485 |
| 137 | 5-(2-Furanyl)-1,2,3,4,5,6-hexahydro-7H-cyclopenta[b]pyridin-7-one | C12H13NO2 | M+H | 204.1021 | 3.1419 | HMDB0039656 | 4.6609 | 4.708 | 4.6977 | 3.4185 | 3.4336 | 3.2048 |
| 138 | Bk-MDEA | C12H15NO3 | M+H | 222.1125 | 3.0782 | HMDB0252083 | 4.8108 | 4.8832 | 4.8896 | 3.3986 | 3.1379 | 3.7131 |
| 139 | Alpha-[3-(Nitrosoamino)propyl]-3-pyridinemethanol | C9H13N3O2 | M+NH4 | 213.1345 | 2.6636 | HMDB0062443 | 5.0669 | 5.0763 | 5.076 | 5.7082 | 5.7322 | 5.7195 |
| 140 | 1-Pyrimidinylpiperazine | C8H12N4 | M+CH3OH+H | 197.1396 | 2.6566 | HMDB0061017 | 2.6905 | 3.1174 | 2.8702 | 5.3779 | 5.3943 | 5.4071 |
| 141 | 4-Carboxypyrazole | C4H4N2O2 | 2M+Na | 247.0423 | 2.4048 | HMDB0060760 | 5.224 | 5.193 | 5.2691 | 4.4716 | 4.391 | 4.5067 |
| 142 | Niacinamide | C6H6N2O | M+H | 123.0555 | 1.5943 | HMDB0001406 | 5.6381 | 5.6421 | 5.6413 | 6.4729 | 6.4777 | 6.4712 |
| 143 | Phaeophorbide b | C35H34N4O6 | M-H, M+Cl, M+Na-2H | 605.2407 | 6.6846 | HMDB0031149 | 5.1161 | 4.933 | 4.7684 | 7.1959 | 7.132 | 7.1098 |
| 144 | Molybdopterin precursor Z | C10H12N5O7P | M-H, 2M-H | 344.0399 | 2.5274 | HMDB0011683 | 4.7808 | 4.6674 | 4.592 | 6.6247 | 6.6452 | 6.6535 |
| 145 | 6,7-Dimethyl-8-(1-D-ribityl)lumazine | C13H18N4O6 | M+FA-H | 371.1196 | 0.9621 | HMDB0003826 | 6.6164 | 6.5881 | 6.6399 | 5.9311 | 5.9528 | 5.9423 |
| 146 | Adenine | C5H5N5 | M-H | 134.0462 | 2.6641 | HMDB0000034 | 5.2886 | 5.3088 | 5.3609 | 6.3269 | 6.3955 | 6.3714 |
| 147 | 3-Pyridinebutanoic acid | C9H11NO2 | M-H | 164.0709 | 3.254 | HMDB0001007 | 3.2348 | 2.8463 | 3.1076 | 3.7522 | 3.7859 | 3.8311 |
| 148 | Alpha-Carboxy-delta-nonalactone | C10H16O4 | M+FA-H | 245.1029 | 3.2752 | HMDB0030990 | 5.3004 | 5.2949 | 5.238 | 4.677 | 4.6443 | 4.6495 |
| 149 | Citreoviridinol A1 | C22H28O8 | M-H2O-H | 401.1602 | 5.5911 | HMDB0035369 | 4.1238 | 4.326 | 4.3192 | 4.9419 | 4.9191 | 4.9903 |
| 150 | 5,10-Methenyltetrahydrofolic acid | C20H21N7O6 | M+FA-H | 500.1559 | 5.6563 | HMDB0001354 | 5.2277 | 5.1775 | 5.2465 | 4.331 | 4.3268 | 4.3191 |
| 151 | 5-(2-Methylpropyl)tetrahydro-2-oxo-3-furancarboxylic acid | C9H14O4 | M-H | 185.0813 | 6.2442 | HMDB0030988 | 5.1881 | 5.1857 | 5.1722 | 3.7897 | 3.7286 | 4.0394 |
| 152 | Colupdox a | C25H36O6 | 2M-H | 863.4927 | 7.4338 | HMDB0039597 | 6.1427 | 6.1405 | 6.193 | 4.1951 | 3.8317 | 3.6336 |
| 153 | (3b,6b,8b,12a)-8,12-Epoxy-7(11)-eremophilene-6-angeloyloxy-8,12-dimethoxy-3-ol | C22H34O6 | 2M+Hac-H | 847.4883 | 7.3768 | HMDB0031964 | 6.0118 | 5.9632 | 6.0577 | 4.641 | 4.5455 | 4.326 |
| 154 | L-Urobilinogen | C33H48N4O6 | M+FA-H | 641.3545 | 6.4348 | HMDB0004157 | 5.1018 | 5.0642 | 5.0652 | 4.399 | 4.1646 | 4.0618 |
| 155 | Hydroxygaleon | C20H22O5 | M+FA-H | 387.1449 | 5.8683 | HMDB0031535 | 5.0279 | 5.0395 | 5.0478 | 4.0058 | 3.9351 | 3.5915 |
| 156 | 3-Methylbutyrolactone | C5H8O2 | 2M+Hac-H | 259.1186 | 5.8614 | HMDB0002167 | 5.5677 | 5.6087 | 5.6581 | 4.8158 | 4.8723 | 4.8534 |
| 157 | 6-Hydroxyetodolac | C17H21NO4 | 2M+Hac-H | 665.3031 | 5.6785 | HMDB0060543 | 3.8772 | 3.2837 | 3.2971 | 5.6242 | 5.6575 | 5.5788 |
| 158 | 5-Methoxyindoleacetate | C11H11NO3 | M+FA-H | 250.0719 | 4.7873 | HMDB0004096 | 5.3262 | 5.3293 | 5.2777 | 4.0149 | 3.9853 | 4.0665 |
| 159 | Citalopram propionic acid | C18H14FNO3 | 2M+FA-H | 667.1884 | 4.447 | HMDB0060463 | 3.4799 | 3.4794 | 3.497 | 5.3087 | 5.4274 | 5.4115 |
| 160 | 6-Hydroxynicotinic acid | C6H5NO3 | 2M-H | 277.0465 | 3.3483 | HMDB0002658 | 2.7623 | 3.3686 | 2.9809 | 4.6937 | 4.6734 | 4.7208 |
| 161 | 3-Furoic acid | C5H4O3 | 2M+FA-H | 269.0303 | 3.254 | HMDB0000444 | 2.9372 | 2.9366 | 2.95 | 4.7091 | 4.7359 | 4.7419 |
| 162 | Adrenochrome | C9H9NO3 | M+FA-H | 224.0561 | 2.8963 | HMDB0012884 | 2.9854 | 2.9849 | 2.9982 | 5.0739 | 5.2169 | 5.2474 |
| 163 | 5-Hydroxy-L-tryptophan | C11H12N2O3 | M-H | 219.0771 | 2.6855 | HMDB0000472 | 5.27 | 5.2213 | 5.1959 | 4.3866 | 4.429 | 4.4736 |
| 164 | 6-Amino-9H-purine-9-propanoic acid | C8H9N5O2 | 2M+Hac-H | 473.162 | 0.9758 | HMDB0029939 | 4.2226 | 4.2221 | 4.2354 | 5.4245 | 5.5757 | 5.5399 |
| 165 | P-Coumaroyl-D-glucose | C15H18O8 | M+Na, 2M+Na, M+H | 349.089 | 3.8293 | HMDB0302089 | 6.0106 | 6.026 | 6.0513 | 6.8779 | 6.9022 | 6.8918 |
| 166 | Aspalathin | C21H24O11 | M+H-H2O, M+H | 453.1385 | 3.6537 | LMPK12120529 | 6.7767 | 6.7908 | 6.7943 | 5.147 | 5.2326 | 5.0322 |
| 167 | Ent-Epicatechin-(4alpha->6)-ent-epicatechin | C30H26O12 | M+H | 579.1492 | 3.2545 | HMDB0041239 | 5.9232 | 6.0179 | 6.0616 | 6.7542 | 6.7204 | 6.7431 |
| 168 | 7-Hydroxy-5-methoxy-6,8-dimethylflavanone | C18H18O4 | M+ACN+Na | 362.1358 | 3.7165 | HMDB0030695 | 3.6526 | 4.0851 | 3.9002 | 4.6756 | 4.587 | 4.5748 |
| 169 | Feruloyl C1-glucuronide | C16H18O10 | M+Na | 393.0789 | 3.7662 | HMDB0041734 | 5.6642 | 5.6891 | 5.6993 | 4.8481 | 4.867 | 4.907 |
| 170 | Cinnamic acid | C9H8O2 | M+H-H2O | 131.0492 | 5.1569 | HMDB0000930 | 3.7347 | 3.7011 | 3.725 | 4.4813 | 4.4936 | 4.4995 |
| 171 | Nodakenin | C20H24O9 | M+H | 409.1464 | 5.1849 | HMDB0258151 | 4.4817 | 4.5896 | 4.515 | 5.2793 | 5.3219 | 5.3077 |
| 172 | Cis-1,2-Diphenylcyclobutane | C16H16 | M+H | 209.1323 | 6.769 | HMDB0031821 | 3.0067 | 3.2789 | 3.0172 | 4.7905 | 4.5544 | 4.4475 |
| 173 | 1,26-Dicaffeoylhexacosanediol | C44H66O8 | M+ACN+Na | 786.4915 | 6.8597 | HMDB0030750 | 3.8841 | 4.4821 | 3.9092 | 5.978 | 5.8136 | 5.8225 |
| 174 | Rubraflavone A | C25H26O5 | M+H-2H2O | 371.1628 | 5.7675 | LMPK12110052 | 4.8599 | 4.8461 | 4.855 | 3.365 | 3.4056 | 3.5678 |
| 175 | Afzelechin 7-apioside | C20H22O9 | M+H | 407.1308 | 4.8342 | HMDB0030824 | 3.3462 | 3.5976 | 3.5092 | 4.4446 | 4.4902 | 4.48 |
| 176 | Cis-Resveratrol | C14H12O3 | M+H | 229.0858 | 4.6437 | HMDB0034118 | 3.9255 | 3.9933 | 3.8051 | 4.8037 | 4.753 | 4.7863 |
| 177 | Zearalenone 4-sulfate | C18H22O8S | M+NH4 | 416.1335 | 4.6227 | HMDB0033623 | 3.5597 | 3.1482 | 3.2541 | 4.507 | 4.5549 | 4.5624 |
| 178 | M-Coumaric acid | C9H8O3 | M+H | 165.0546 | 3.6815 | HMDB0001713 | 5.9063 | 5.9322 | 5.9467 | 6.7187 | 6.7205 | 6.7155 |
| 179 | Liquiritin | C21H22O9 | M+NH4 | 436.1599 | 2.8108 | LMPK12140021 | 4.0088 | 4.013 | 3.7831 | 4.6766 | 4.7076 | 4.7372 |
| 180 | Caffeic Acid | C9H8O4 | M+H-H2O, M+H | 163.0388 | 3.2614 | HMDB0001964 | 4.8743 | 4.9492 | 4.9263 | 5.5492 | 5.5214 | 5.5734 |
| 181 | 6-Methylcoumarin | C10H8O2 | M-H, M+FA-H | 205.05 | 3.7054 | HMDB0032394 | 3.2655 | 3.5214 | 3.2958 | 4.7216 | 4.809 | 4.6909 |
| 182 | (S)-Rutaretin | C14H14O5 | M-H2O-H, M-H | 261.0769 | 2.9615 | HMDB0030882 | 3.2945 | 0.0091 | 0.0094 | 6.149 | 6.0621 | 6.1599 |
| 183 | Caffeic acid 3-sulfate | C9H8O7S | M-H2O-H | 240.9838 | 2.3645 | HMDB0041706 | 3.2064 | 3.2059 | 3.2775 | 4.4185 | 4.4467 | 4.5463 |
| 184 | Isoneotheaflavin | C29H24O12 | M+FA-H | 609.1251 | 2.9478 | HMDB0029256 | 6.3173 | 6.2329 | 6.2598 | 7.371 | 7.2319 | 7.386 |
| 185 | 3-HYDROXYCOUMARIN | C9H6O3 | M-H | 161.0236 | 3.2683 | HMDB0002149 | 3.9746 | 3.7741 | 3.8638 | 4.9421 | 4.968 | 4.9411 |
| 186 | 3 Hydroxycoumarin | C9H6O3 | M+FA-H | 207.0294 | 3.7329 | HMDB0002149 | 2.8849 | 2.8844 | 2.8977 | 4.2171 | 4.1586 | 4.246 |
| 187 | B-D-fructosyl-a-D-(6-O-(E))-feruloylglucoside | C21H28O12 | M+FA-H | 517.1559 | 3.9705 | HMDB0029281 | 3.2873 | 3.2867 | 3.3001 | 5.0123 | 4.9199 | 5.0537 |
| 188 | 15-Hexadecanolide | C16H30O2 | M+Na-2H | 275.2016 | 6.585 | HMDB0031711 | 5.2814 | 5.2725 | 5.3418 | 4.5278 | 4.5149 | 4.5712 |
| 189 | Trans-Caffeic acid [apiosyl-(1->6)-glucosyl] ester | C20H26O13 | 2M-H | 947.2608 | 5.7369 | HMDB0033694 | 4.6318 | 4.4048 | 4.2727 | 5.1278 | 5.183 | 5.1175 |
| 190 | ESCULETIN | C9H6O4 | M-H | 177.0186 | 3.8839 | HMDB0030819 | 5.8018 | 5.7915 | 5.8184 | 4.5863 | 4.6291 | 4.6067 |
| 191 | AESCULIN | C15H16O9 | M-H | 339.072 | 3.3161 | HMDB0030820 | 4.1275 | 4.0515 | 4.0013 | 4.7083 | 4.6885 | 4.7122 |
| 192 | (+/-)-Catechin | C15H14O6 | M-H, M+Cl | 289.0717 | 3.9498 | HMDB0002780 | 6.0298 | 5.9891 | 5.9792 | 6.7865 | 6.7108 | 6.7115 |
| 193 | EPIGALLOCATECHIN | C15H14O7 | M-H, 2M-H, M+Cl | 305.0666 | 3.3626 | LMPK12020004 | 5.618 | 5.4718 | 5.5979 | 7.7532 | 7.7713 | 7.7585 |
| 194 | MARMESIN | C14H14O4 | M-H | 245.0817 | 3.9498 | HMDB0302268 | 3.7323 | 3.7111 | 3.6075 | 4.757 | 4.7007 | 4.7167 |
| 195 | P-Hydroxymandelic acid | C8H8O4 | M+H-2H2O, M+H-H2O, M+H | 169.0495 | 2.9515 | HMDB0000822 | 4.1003 | 4.1946 | 4.142 | 6.001 | 5.9802 | 5.9755 |
| 196 | 2-Isopropylphenyl methylcarbamate | C11H15NO2 | M+H-2H2O, M+ACN+Na | 257.1281 | 8.7389 | HMDB0031797 | 5.3278 | 5.3424 | 5.33 | 4.4559 | 4.4437 | 4.5258 |
| 197 | (R)-Pterosin B | C14H18O2 | M+H-H2O, M+H | 201.1273 | 4.8768 | HMDB0030759 | 3.3761 | 3.6077 | 3.7928 | 5.0242 | 5.113 | 5.063 |
| 198 | N-Acetylprocainamide | C15H23N3O2 | M+2Na-H | 322.1493 | 2.2931 | HMDB0041944 | 5.6149 | 5.6412 | 5.6293 | 4.9718 | 4.9663 | 4.983 |
| 199 | Galactosylhydroxylysine | C12H24N2O8 | M+H-H2O | 307.1502 | 2.8108 | HMDB0246151 | 4.7517 | 4.7725 | 4.7569 | 3.6206 | 3.9259 | 3.8699 |
| 200 | DIPHENYLUREA | C13H12N2O | M+H | 213.1023 | 4.2495 | HMDB0032066 | 3.8895 | 3.7607 | 3.7771 | 4.586 | 4.6149 | 4.6032 |
| 201 | Norfluoxetine | C16H16F3NO | M+H | 296.1276 | 6.706 | HMDB0060551 | 3.4933 | 3.5201 | 3.5184 | 5.2045 | 4.9851 | 4.9161 |
| 202 | 3-Methyl-1-phenyl-3-pentanol | C12H18O | M+H | 179.1429 | 6.6362 | HMDB0031731 | 4.9442 | 5.0156 | 5.0935 | 4.2213 | 3.911 | 4.0892 |
| 203 | Metanephrine | C10H15NO3 | M+H | 198.1124 | 4.7565 | HMDB0004063 | 3.8455 | 3.7165 | 3.8201 | 4.8843 | 4.8251 | 4.8475 |
| 204 | (-)-Epinephrine | C9H13NO3 | M+H | 184.0968 | 3.8223 | HMDB0000068 | 3.9781 | 3.9743 | 4.0039 | 4.6703 | 4.6482 | 4.6596 |
| 205 | 4-Hydroxy-3,5-dimethoxybenzaldehyde | C9H10O4 | M+H | 183.0652 | 3.7586 | HMDB0258653 | 4.2114 | 4.2369 | 4.2417 | 4.8619 | 4.8494 | 4.8561 |
| 206 | 4-Hydroxy-3-methoxycinnamaldehyde | C10H10O3 | M+H | 179.0702 | 3.3373 | HMDB0141782 | 4.8205 | 4.9082 | 4.9191 | 5.5843 | 5.573 | 5.5993 |
| 207 | Phenylmethyl 2-methylpropanoate | C11H14O2 | M+ACN+H | 220.1331 | 3.1846 | HMDB0031250 | 5.247 | 5.2725 | 5.2854 | 2.6646 | 2.971 | 2.654 |
| 208 | M-Salicylic acid | C7H6O3 | M+H | 139.039 | 2.9585 | HMDB0002466 | 5.0301 | 5.0259 | 5.0197 | 6.359 | 6.3404 | 6.3482 |
| 209 | N,N-Didesmethyltramadol | C14H21NO2 | M+ACN+Na | 299.171 | 2.7758 | HMDB0060849 | 3.1715 | 3.6064 | 3.3746 | 5.2052 | 5.2057 | 5.221 |
| 210 | Normetanephrine | C9H13NO3 | M-H2O-H | 164.0709 | 2.8578 | HMDB0000819 | 4.1319 | 4.1079 | 4.2169 | 5.007 | 5.0228 | 4.9932 |
| 211 | 4-Methylcatechol | C7H8O2 | M-H | 123.0441 | 2.8854 | HMDB0000873 | 3.87 | 3.8533 | 3.8391 | 4.5546 | 4.4582 | 4.5103 |
| 212 | N-Desmethyl-O-hydroxy rosiglitazone | C17H17N3O4S | M+Cl | 394.0625 | 2.9028 | HMDB0060836 | 3.3301 | 3.4566 | 3.1041 | 4.8901 | 4.8864 | 4.8517 |
| 213 | 3,17-Androstanediol glucuronide | C25H40O8 | M+Cl | 503.242 | 6.6662 | LMST05010037 | 3.627 | 3.6265 | 3.6398 | 5.4119 | 5.4487 | 5.4454 |
| 214 | Uridine 5'-monophosphate | C9H13N2O9P | M-H2O-H | 305.018 | 1.5247 | 58-97-9 | 5.212 | 5.135 | 5.2199 | 6.6096 | 6.6611 | 6.6574 |
| 215 | 5-Methyldeoxycytidine | C10H15N3O4 | M+H-H2O, M+ACN+Na, M+ACN+H | 283.1397 | 2.8388 | HMDB0002224 | 4.4727 | 4.4607 | 4.5694 | 5.1353 | 5.1464 | 5.1485 |
| 216 | 1-Methyladenosine | C11H15N5O4 | M+H | 282.1194 | 2.7758 | HMDB0003331 | 4.1872 | 4.1936 | 4.1521 | 4.7692 | 4.8151 | 4.8149 |
| 217 | Isopentenyl adenosine | C15H21N5O4 | M+H | 336.1661 | 5.2058 | HMDB0304396 | 3.5587 | 3.8077 | 3.4801 | 5.1158 | 5.1573 | 5.1666 |
| 218 | CGMP | C10H12N5O7P | M+H | 346.0544 | 2.5098 | HMDB0001314 | 4.9181 | 4.719 | 4.8074 | 6.3659 | 6.361 | 6.3612 |
| 219 | Adenosine | C10H13N5O4 | M+Cl, M+FA-H, M-H | 312.0949 | 2.6596 | HMDB0247583 | 4.8966 | 4.9475 | 4.9571 | 7.0213 | 7.0766 | 7.0448 |
| 220 | Lamivudine sulfoxide | C8H11N3O4S | M+Na-2H | 266.0201 | 3.33 | HMDB0060599 | 5.2755 | 5.2278 | 5.2962 | 4.5483 | 4.5204 | 4.5561 |
| 221 | Blumenol C O-[apiosyl-(1->6)-glucoside] | C24H40O11 | M+H-H2O, M+Na, M+NH4, M+H-2H2O | 527.2459 | 6.1613 | HMDB0031936 | 4.819 | 5.0139 | 4.8351 | 6.0415 | 5.9973 | 6.0298 |
| 222 | Propyl 2,4-decadienoate | C13H22O2 | M+H, M+NH4, M+H-H2O, M+H-2H2O | 228.1956 | 6.3783 | LMFA07011001 | 6.931 | 6.93 | 6.9464 | 6.3549 | 6.3404 | 6.3686 |
| 223 | Kojibiose | C12H22O11 | M+H-2H2O, M+NH4, M+H-H2O | 360.1496 | 2.2441 | HMDB0011742 | 5.7172 | 5.7111 | 5.6572 | 6.7097 | 6.7043 | 6.7047 |
| 224 | Gamma-Tocotrienol | C28H42O2 | M+H-H2O, M+H, M+H-2H2O | 393.3147 | 6.2033 | LMPR02020057 | 5.9276 | 5.9532 | 5.9358 | 5.0094 | 4.9897 | 4.9981 |
| 225 | LysoPE(18:2(9Z,12Z)/0:0) | C23H44NO7P | M+H, M+Na, M+H-H2O | 478.2922 | 6.713 | HMDB0011507 | 5.685 | 5.7308 | 5.7633 | 7.0618 | 6.9691 | 6.9862 |
| 226 | Dehydrocyanaropicrin | C19H20O6 | M+H-H2O, M+Na, M+H-2H2O | 367.1172 | 4.1176 | HMDB0035029 | 6.1651 | 6.2322 | 6.2289 | 4.5214 | 4.498 | 4.5696 |
| 227 | Sorbitan palmitate | C22H42O6 | M+2H, M+H | 202.1569 | 5.3107 | LMFA07011019 | 4.8565 | 4.9726 | 5.0106 | 2.7817 | 2.7654 | 2.7711 |
| 228 | 3,11,12-Trihydroxy-1(10)-spirovetiven-2-one | C15H24O4 | M+H-H2O, M+NH4 | 251.1639 | 6.1265 | HMDB0038154 | 5.6266 | 5.6247 | 5.6329 | 4.915 | 4.9198 | 4.9663 |
| 229 | 16(R)-HETE | C20H32O3 | M+NH4, M+Na | 343.2264 | 6.5384 | HMDB0004680 | 6.3759 | 6.3792 | 6.3982 | 5.6159 | 5.6137 | 5.5936 |
| 230 | PE(20:4(8Z,11Z,14Z,17Z)/P-18:0) | C43H78NO7P | M+K, M+2Na-H | 796.5476 | 7.0589 | HMDB0009445 | 5.1509 | 5.5518 | 5.306 | 6.1213 | 6.1099 | 6.0601 |
| 231 | PC(18:2(9Z,12Z)/18:4(6Z,9Z,12Z,15Z)) | C44H76NO8P | M+H, M+Na | 778.5376 | 7.4336 | LMGP01011627 | 6.0468 | 6.0391 | 5.9699 | 7.8218 | 7.8322 | 7.8094 |
| 232 | PC(18:2(9Z,12Z)/18:3(9Z,12Z,15Z)) | C44H78NO8P | M+H, M+Na | 780.5527 | 7.5943 | LMGP01011626 | 6.9011 | 6.9925 | 6.9572 | 8.7058 | 8.6781 | 8.7306 |
| 233 | PE(16:1(9Z)/20:1(11Z)) | C41H78NO8P | M+H, M+Na | 744.5531 | 7.7327 | LMGP02010530 | 5.9001 | 6.0827 | 6.1453 | 6.6942 | 6.6524 | 6.7269 |
| 234 | TG(18:3/18:3/18:3) | C57H92O6 | M+NH4, M+Na | 890.7222 | 8.1909 | HMDB0053167 | 4.9339 | 4.6323 | 3.8498 | 6.2305 | 6.15 | 6.1661 |
| 235 | DG(18:3(6Z,9Z,12Z)/18:3(9Z,12Z,15Z)/0:0) | C39H64O5 | M+H-H2O, M+H | 613.482 | 8.8509 | HMDB0007279 | 5.4887 | 5.2362 | 5.5223 | 6.2421 | 6.2014 | 6.3899 |
| 236 | 5,7-Megastigmadien-9-ol glucoside | C19H32O6 | M+H, M+CH3OH+H | 357.2242 | 7.1784 | HMDB0041044 | 5.5436 | 5.6007 | 5.59 | 4.9167 | 4.8735 | 4.9231 |
| 237 | Beta-Doradecin | C40H52O3 | M+H-H2O, M+H | 581.3983 | 6.6572 | HMDB0039143 | 6.3233 | 6.312 | 6.325 | 7.2812 | 7.1212 | 7.1218 |
| 238 | 7-O-Acetylaustroinulin | C22H36O4 | M+H-H2O, M+H | 365.268 | 6.6292 | HMDB0036804 | 5.8726 | 5.8955 | 5.9232 | 5.071 | 4.8831 | 4.7844 |
| 239 | 7,8-Dehydroastaxanthianthin | C40H50O4 | M+H-2H2O, M+NH4 | 559.3627 | 6.5035 | HMDB0036872 | 6.3763 | 6.4205 | 6.4378 | 5.5584 | 5.5419 | 5.5081 |
| 240 | Erinacine D | C27H42O7 | M+H-H2O, M+Na | 461.2889 | 6.441 | HMDB0031896 | 6.4191 | 6.4554 | 6.4697 | 5.2493 | 5.2217 | 5.2161 |
| 241 | Lucidone C | C24H36O5 | M+H-H2O, M+H | 405.2628 | 6.3992 | HMDB0038152 | 6.0584 | 6.0804 | 6.0992 | 3.4419 | 3.8295 | 3.8731 |
| 242 | 16-Hydroxy-10-oxohexadecanoic acid | C16H30O4 | M+H, M+Na | 309.2056 | 6.3365 | HMDB0041287 | 6.4408 | 6.4591 | 6.4629 | 5.2871 | 5.2824 | 5.2845 |
| 243 | Annuolide E | C15H20O3 | M+H, M+CH3OH+H | 281.1743 | 6.2103 | HMDB0038326 | 5.8964 | 5.9189 | 5.9142 | 4.5592 | 4.4888 | 4.5876 |
| 244 | Leukotriene E3 | C23H39NO5S | M+Na, M+ACN+H | 483.2807 | 5.647 | LMFA03020074 | 6.0202 | 6.0594 | 6.0855 | 4.75 | 4.6967 | 4.795 |
| 245 | Beta-Ionone | C13H20O | M+H-H2O, M+H | 193.1587 | 3.9973 | HMDB0036565 | 4.7238 | 4.765 | 4.7642 | 5.5114 | 5.5416 | 5.5435 |
| 246 | Malonylcarnitine | C10H17NO6 | M+CH3OH+H | 280.139 | 2.6988 | LMFA07070080 | 4.7644 | 4.8163 | 4.787 | 4.1751 | 4.0427 | 3.9873 |
| 247 | 3-Hydroxyisovalerylcarnitine | C12H24NO5+ | M+Na | 285.1554 | 2.9158 | LMFA07070041 | 4.2717 | 4.1537 | 4.0897 | 4.8202 | 4.7671 | 4.851 |
| 248 | (2E,11Z)-5-[5-(Methylthio)-4-penten-2-ynyl]-2-furanacrolein | C13H12O2S | M+H-H2O | 215.0526 | 3.0005 | HMDB0031070 | 2.7695 | 2.866 | 2.7945 | 4.4279 | 4.3743 | 4.449 |
| 249 | Sarmentosin | C11H17NO7 | M+H-2H2O | 240.0865 | 3.0362 | HMDB0030697 | 4.0785 | 4.0548 | 4.0351 | 4.6442 | 4.637 | 4.6066 |
| 250 | Heterocodeine | C18H21NO3 | M+H | 300.1591 | 3.5415 | HMDB0029382 | 3.6248 | 3.4212 | 3.5612 | 4.4136 | 4.4065 | 4.4105 |
| 251 | 5-Hydroxy-p-mentha-6,8-dien-2-one | C10H14O2 | M+NH4 | 184.1332 | 3.7095 | HMDB0037011 | 5.0194 | 5.0503 | 5.0354 | 2.5374 | 2.9791 | 2.5268 |
| 252 | (Z)-3-(1-Formyl-1-propenyl)pentanedioic acid | C9H12O5 | M+ACN+H | 242.1021 | 4.1106 | HMDB0033091 | 3.0293 | 3.3983 | 3.1286 | 5.2506 | 5.2453 | 5.2429 |
| 253 | (2S,4R)-p-Mentha-1(7),5-dien-2-ol acetate | C12H18O2 | M+NH4 | 212.1644 | 4.7355 | HMDB0035801 | 4.4551 | 4.4862 | 4.431 | 5.2414 | 5.2601 | 5.2652 |
| 254 | 12a-Hydroxy-3-oxocholadienic acid | C24H34O4 | M+Na | 409.2327 | 4.8272 | HMDB0000385 | 5.2298 | 5.2463 | 5.2823 | 3.236 | 3.0145 | 3.0203 |
| 255 | Procurcumadiol | C15H22O3 | M+ACN+H | 292.1904 | 5.0869 | HMDB0034721 | 5.1509 | 5.1288 | 5.1658 | 3.7821 | 3.7012 | 3.8577 |
| 256 | Neoacrimarine F | C29H25NO9 | M+NH4 | 549.1903 | 5.2058 | HMDB0031112 | 4.8728 | 4.909 | 4.7942 | 2.416 | 2.3997 | 2.4054 |
| 257 | Cinnamoside | C24H38O12 | M+H | 519.2407 | 5.3177 | HMDB0038923 | 6.3435 | 6.3515 | 6.381 | 3.6085 | 3.1615 | 3.1672 |
| 258 | (4S,6R)-p-Mentha-1,8-diene-6,7-diol 7-glucoside | C16H26O7 | M+NH4 | 348.2023 | 5.3247 | HMDB0039056 | 5.3231 | 5.3523 | 5.3348 | 4.0745 | 4.5675 | 4.56 |
| 259 | Pregnanetriolone | C21H34O4 | M+K | 389.2066 | 5.4084 | HMDB0041997 | 5.6503 | 5.6591 | 5.6628 | 3.2194 | 3.203 | 3.2088 |
| 260 | Spirotaccagenin | C27H42O5 | M+H-H2O | 429.2994 | 6.2103 | HMDB0034424 | 5.7593 | 5.7597 | 5.725 | 4.7001 | 4.6194 | 4.5256 |
| 261 | 11-Hydroxy-9-tridecenoic acid | C13H24O3 | M+H | 229.1798 | 6.3853 | LMFA01050437 | 5.0868 | 5.0813 | 5.1421 | 4.4775 | 4.4359 | 4.4715 |
| 262 | 9(S)-HPODE | C18H32O4 | M+NH4 | 330.2635 | 6.4756 | HMDB0006940 | 6.0033 | 6.03 | 6.0475 | 4.94 | 4.8191 | 4.8108 |
| 263 | 5,6-epoxy,18R-HEPE | C20H28O4 | 2M+H | 665.4045 | 6.4895 | HMDB0062410 | 4.8808 | 5.026 | 5.0274 | 6.4877 | 6.5112 | 6.5212 |
| 264 | 10-Epijunenol | C15H26O | M+NH4 | 240.2319 | 6.4965 | HMDB0034982 | 5.295 | 5.3263 | 5.3633 | 5.9621 | 5.9513 | 5.967 |
| 265 | (1beta,2alpha,3alpha)-1,2,3,24-Tetrahydroxy-12-oleanen-28-oic acid | C30H48O6 | M+H | 505.3514 | 6.5245 | HMDB0035487 | 6.3529 | 6.3914 | 6.4326 | 5.5265 | 5.5297 | 5.4709 |
| 266 | Kanokoside D | C27H44O16 | M+H | 625.2649 | 6.5454 | HMDB0036105 | 5.2861 | 5.3553 | 5.377 | 6.3052 | 6.2766 | 6.2715 |
| 267 | Hebevinoside XIV | C47H74O15 | M+H-H2O | 861.4957 | 6.5594 | HMDB0034604 | 6.3911 | 6.4345 | 6.4596 | 5.8806 | 5.821 | 5.7513 |
| 268 | 16-Oxoandrostenediol | C19H28O3 | 2M+Na | 631.399 | 6.6083 | HMDB0000322 | 5.3843 | 5.3234 | 5.3043 | 6.3828 | 6.3049 | 6.2992 |
| 269 | 3-(2-Heptenyloxy)-2-hydroxypropyl undecanoate | C21H40O4 | M+Na | 379.2836 | 6.6362 | HMDB0034031 | 5.2819 | 5.3496 | 5.3673 | 4.597 | 4.286 | 4.4974 |
| 270 | Nutriacholic acid | C24H38O4 | M+CH3OH+H | 423.3091 | 6.6362 | HMDB0000467 | 5.8884 | 5.9325 | 5.9445 | 4.8935 | 4.6577 | 4.6472 |
| 271 | Rubinic acid | C30H46O4 | M+Na | 493.3305 | 6.748 | HMDB0036655 | 5.589 | 5.6149 | 5.6218 | 6.3432 | 6.13 | 6.1382 |
| 272 | Beta-Citraurin epoxide | C30H40O3 | M+H-H2O | 431.2943 | 6.755 | HMDB0039079 | 4.8446 | 4.8519 | 4.8569 | 5.6729 | 5.398 | 5.4187 |
| 273 | Alpha-Solamarine | C45H73NO16 | M+ACN+H | 925.5316 | 6.755 | HMDB0258364 | 6.5039 | 6.5985 | 6.5957 | 5.4143 | 4.6318 | 4.6037 |
| 274 | Persicachrome | C25H36O3 | M+H | 385.2727 | 6.776 | HMDB0036425 | 3.9774 | 4.2544 | 4.3229 | 5.205 | 4.7837 | 4.7271 |
| 275 | Desglucocheirotoxin | C29H42O10 | M+K | 589.2446 | 6.8249 | HMDB0034362 | 6.3829 | 6.5236 | 6.527 | 5.7755 | 5.7373 | 5.7347 |
| 276 | PA(14:0/22:6(4Z,7Z,10Z,13Z,16Z,19Z)) | C39H65O8P | M+H | 693.4481 | 6.8457 | HMDB0114792 | 6.0044 | 6.2746 | 6.2888 | 4.0949 | 4.0785 | 4.0842 |
| 277 | 12-Ketodeoxycholic acid | C24H38O4 | M+CH3OH+H | 423.3099 | 6.8597 | HMDB0000328 | 5.6212 | 5.8533 | 5.9022 | 4.6195 | 4.3594 | 4.4569 |
| 278 | Deoxycholic acid 3-glucuronide | C30H48O10 | M+K | 607.291 | 6.8945 | HMDB0002596 | 8.0269 | 8.2133 | 8.2375 | 7.1016 | 6.8539 | 6.8266 |
| 279 | PE(DiMe(9,3)/DiMe(9,5)) | C43H74NO10P | M+CH3OH+H | 828.5384 | 6.9154 | HMDB0061502 | 4.2248 | 4.4483 | 4.4381 | 6.2679 | 6.3358 | 6.31 |
| 280 | Absindiol | C15H22O4 | M+H | 267.1581 | 6.9435 | HMDB0033229 | 4.917 | 5.1096 | 5.0826 | 4.417 | 4.2286 | 4.0996 |
| 281 | PC(18:0/0:0) | C26H54NO7P | M+H | 524.3705 | 7.069 | HMDB0010384 | 5.84 | 5.9717 | 5.9468 | 6.8704 | 6.8453 | 6.8048 |
| 282 | MG(0:0/22:4(7Z,10Z,13Z,16Z)/0:0) | C25H42O4 | M+CH3OH+H | 439.3412 | 7.1574 | HMDB0011554 | 6.7032 | 6.7437 | 6.7616 | 5.8653 | 5.8208 | 5.8475 |
| 283 | GPEtn(18:3/18:3) | C41H70NO8P | M+H | 736.4902 | 7.1574 | HMDB0009127 | 4.3494 | 4.3221 | 4.206 | 6.161 | 6.0339 | 6.1624 |
| 284 | GPEtn(15:0/20:3) | C40H74NO8P | M+H | 728.5215 | 7.4826 | HMDB0008903 | 4.6113 | 4.4296 | 4.3243 | 5.7151 | 5.6402 | 5.7235 |
| 285 | DG(16:0/18:2(9Z,12Z)/0:0) | C37H68O5 | M+H-H2O | 575.5025 | 7.5322 | HMDB0007103 | 5.5588 | 5.6063 | 5.5569 | 6.5052 | 6.4471 | 6.4993 |
| 286 | PC(16:0/18:3(9Z,12Z,15Z)) | C42H78NO8P | M+H | 756.5531 | 7.9471 | LMGP01010601 | 4.8732 | 4.6025 | 4.5953 | 8.0993 | 7.9947 | 8.1365 |
| 287 | Ganoderic acid F | C32H42O9 | M+Na | 593.2749 | 8.0938 | HMDB0035988 | 4.8154 | 4.7866 | 4.8258 | 5.3864 | 5.4351 | 5.3573 |
| 288 | PC(14:1(9Z)/22:2(13Z,16Z)) | C44H82NO8P | M+H | 784.5825 | 8.2101 | HMDB0007921 | 5.6573 | 5.619 | 5.6269 | 6.6106 | 6.6179 | 6.4636 |
| 289 | DG(22:0/16:1(9Z)/0:0) | C41H78O5 | M+NH4 | 668.6178 | 8.2267 | HMDB0007592 | 5.9915 | 6.0112 | 6.0094 | 5.2129 | 5.0558 | 5.2173 |
| 290 | PE(16:1(9Z)/P-18:1(11Z)) | C39H74NO7P | M+CH3OH+H | 732.5526 | 8.2856 | HMDB0008984 | 5.1794 | 5.1327 | 4.9091 | 5.9894 | 5.9986 | 5.9767 |
| 291 | PI(16:0/18:2(9Z,12Z)) | C43H79O13P | M+Na | 857.5143 | 8.3639 | LMGP06010959 | 6.1389 | 6.1921 | 6.2829 | 3.3479 | 3.2356 | 3.2414 |
| 292 | PS(DiMe(11,3)/MonoMe(13,5)) | C49H84NO12P | M+H-2H2O | 874.5535 | 8.7529 | HMDB0061559 | 4.9562 | 4.5428 | 4.3037 | 6.3768 | 6.4156 | 6.349 |
| 293 | GPCho(14:0/22:5) | C44H78NO8P | M+Na | 802.5348 | 8.7949 | HMDB0007890 | 4.9034 | 4.4986 | 4.2573 | 6.0069 | 5.9584 | 5.9852 |
| 294 | GPEtn(18:1/18:1) | C41H78NO8P | M+H | 744.5524 | 8.8089 | HMDB0009025 | 4.8071 | 3.9795 | 3.9777 | 5.725 | 5.7529 | 5.7081 |
| 295 | GPEtn(14:0/20:3) | C39H72NO8P | M+H | 714.5063 | 8.8509 | HMDB0008837 | 4.7637 | 4.9472 | 4.9855 | 5.9181 | 5.9093 | 5.8955 |
| 296 | 1-Linoleoylglycerophosphocholine | C26H50NO7P | M+H | 520.3392 | 8.8369 | HMDB0010386 | 3.587 | 3.6138 | 3.7603 | 6.0829 | 6.047 | 6.0984 |
| 297 | PC(18:0/18:3(9Z,12Z,15Z)) | C44H82NO8P | M+H | 784.584 | 8.7809 | LMGP01011589 | 6.7034 | 6.2472 | 6.1671 | 8.0456 | 8.009 | 8.0282 |
| 298 | Artemoin A | C35H66O4 | M+Na | 573.4869 | 7.9471 | HMDB0033604 | 5.2295 | 5.3321 | 5.1751 | 6.3441 | 6.293 | 6.3019 |
| 299 | PE(22:4(7Z,10Z,13Z,16Z)/22:6(4Z,7Z,10Z,13Z,16Z,19Z)) | C49H78NO8P | M+ACN+Na | 903.5622 | 7.7141 | LMGP02011272 | 5.9883 | 6.1754 | 6.2199 | 4.1985 | 4.1821 | 4.1879 |
| 300 | Cryptoxanthin epoxide | C40H56O2 | M+K | 607.3903 | 7.5377 | HMDB0030538 | 5.9048 | 6.0256 | 6.0726 | 5.22 | 5.0357 | 5.1538 |
| 301 | Artemoin C | C35H66O4 | M+Na | 573.4868 | 7.3943 | HMDB0033606 | 5.2213 | 5.2321 | 5.2374 | 6.1628 | 6.1403 | 6.1371 |
| 302 | PE(16:1(9Z)/22:6(4Z,7Z,10Z,13Z,16Z,19Z)) | C43H72NO8P | M+H | 762.5034 | 7.3761 | HMDB0008979 | 4.8612 | 4.9424 | 4.801 | 5.599 | 5.5576 | 5.5562 |
| 303 | PS(22:2(13Z,16Z)/22:6(4Z,7Z,10Z,13Z,16Z,19Z)) | C50H82NO10P | M+ACN+Na | 951.5839 | 7.3278 | HMDB0112785 | 5.4062 | 5.4049 | 5.3778 | 6.0708 | 6.0075 | 6.0616 |
| 304 | Isozeaxanthin | C40H56O2 | M+H | 569.4346 | 7.2582 | HMDB0036922 | 4.2971 | 4.6292 | 4.6717 | 6.0817 | 6.0031 | 6.0618 |
| 305 | 13-HDoHE | C22H34O | 2M+ACN+H | 670.5605 | 7.1861 | HMDB0060043 | 6.0323 | 6.1776 | 6.122 | 4.2988 | 3.957 | 4.23 |
| 306 | 6-Deoxohomodolichosterone | C29H50O4 | M+H | 463.3775 | 7.076 | HMDB0034430 | 4.0947 | 4.5373 | 4.5125 | 5.1415 | 5.0635 | 5.046 |
| 307 | Cucurbitaxanthin A | C40H56O3 | M+H-H2O | 567.4191 | 6.9644 | LMPR01070064 | 4.9996 | 5.2751 | 5.3317 | 6.279 | 6.1113 | 6.0229 |
| 308 | Spirolide E | C43H67NO8 | M+Na | 748.4753 | 6.8666 | HMDB0030494 | 3.8054 | 3.8322 | 3.8895 | 5.4837 | 5.3827 | 5.382 |
| 309 | 2-Palmitoylglycerophosphocholine | C24H51NO7P+ | M+H-H2O | 479.3338 | 6.8387 | HMDB0240262 | 5.6472 | 5.9513 | 5.9381 | 4.7675 | 4.3595 | 4.4034 |
| 310 | Cer(d18:0/16:0) | C34H69NO3 | M+H | 540.5343 | 6.8109 | LMSP02020001 | 6.7446 | 6.7894 | 6.7543 | 6.0934 | 5.9173 | 5.9159 |
| 311 | PA(14:1(9Z)/22:6(4Z,7Z,10Z,13Z,16Z,19Z)) | C39H63O8P | M+H | 691.4339 | 6.7969 | LMGP10010136 | 5.8919 | 6.0671 | 6.0563 | 4.5437 | 4.5923 | 4.3001 |
| 312 | 13-Nor-6-eremophilene-8,11-dione | C14H20O2 | M+H | 221.1534 | 6.755 | HMDB0037606 | 3.9139 | 4.093 | 4.0419 | 5.3085 | 5.0645 | 5.0734 |
| 313 | Methyl 3b,24-dihydroxy-11,13(18)-oleanadien-30-oate | C31H48O4 | M+Na | 507.3466 | 6.748 | HMDB0035257 | 5.2904 | 5.2912 | 5.3107 | 6.3997 | 6.1513 | 6.1644 |
| 314 | Beta-Micropteroxanthin | C27H40O2 | M+Na | 419.2939 | 6.748 | HMDB0038505 | 4.4319 | 4.1593 | 4.0381 | 5.5242 | 5.3011 | 5.281 |
| 315 | Alkhanol | C15H22O4 | M+H | 267.1585 | 6.699 | HMDB0036152 | 5.6853 | 5.7016 | 5.7004 | 5.0932 | 4.9329 | 4.99 |
| 316 | Physagulin G | C36H50O15 | M+H-2H2O | 687.3016 | 6.6782 | HMDB0039694 | 6.5604 | 6.5754 | 6.6002 | 5.2459 | 4.807 | 4.8008 |
| 317 | PC(14:0/0:0) | C22H46NO7P | M+H | 468.3078 | 6.6642 | HMDB0010379 | 3.5566 | 3.9971 | 3.1934 | 5.3776 | 5.3126 | 5.3131 |
| 318 | Actinidic acid | C30H46O5 | M+Na | 509.3257 | 6.6222 | HMDB0037963 | 2.9855 | 3.3565 | 3.0105 | 6.1928 | 6.0963 | 6.071 |
| 319 | Lucidenic acid D2 | C29H38O8 | M+H | 515.2648 | 6.5944 | HMDB0036435 | 5.1346 | 5.1613 | 5.1596 | 6.435 | 6.3348 | 6.3677 |
| 320 | MG(0:0/18:4(6Z,9Z,12Z,15Z)/0:0) | C21H34O4 | M+H | 351.2525 | 6.5663 | HMDB0011541 | 6.2779 | 6.2978 | 6.3184 | 5.1374 | 5.0782 | 5.063 |
| 321 | 11-Dodecenoic acid | C12H22O2 | 2M+NH4 | 414.3572 | 6.5594 | LMFA01030043 | 5.4466 | 5.4739 | 5.497 | 4.8894 | 4.8101 | 4.8507 |
| 322 | 11-alpha-O-beta-D-Glucopyranosyl-16alpha-O-methylneoquassin | C29H44O11 | M+2Na-H | 613.2651 | 6.5245 | HMDB0039773 | 3.9764 | 4.0032 | 4.0015 | 5.8561 | 5.8589 | 5.8774 |
| 323 | Ginsenoside F1 | C36H62O9 | M+2Na-H | 683.4147 | 6.4895 | HMDB0039555; | 3.7285 | 3.7553 | 3.7536 | 6.0385 | 5.9916 | 6.0253 |
| 324 | 2,3-Dinor-6-keto-prostaglandin F1 a | C18H30O6 | M+H-H2O | 325.2005 | 6.441 | HMDB0002277 | 5.7055 | 5.7391 | 5.7337 | 4.451 | 4.3225 | 4.4587 |
| 325 | 4-hydroxyestradiol | C18H24O3 | M+H | 289.1794 | 6.3992 | LMST02010028 | 5.5966 | 5.5855 | 5.6247 | 4.2398 | 4.2126 | 4.2151 |
| 326 | 10,20-Dihydroxyeicosanoic acid | C20H40O4 | M+NH4 | 362.3259 | 6.3645 | HMDB0031923 | 6.7108 | 6.7086 | 6.7211 | 6.0568 | 6.0635 | 6.0414 |
| 327 | (S)-3-Octanol glucoside | C14H28O6 | 2M+K | 623.3421 | 6.3365 | HMDB0032958 | 6.2074 | 6.25 | 6.2515 | 4.2007 | 3.8227 | 4.4023 |
| 328 | (12S,15S)-15-O-Demethyl-10,29-dideoxy-11,12-dihydro-striatin C | C25H38O6 | M+NH4 | 452.3003 | 6.3365 | HMDB0033030 | 5.5564 | 5.5407 | 5.4696 | 4.7505 | 4.8577 | 4.7845 |
| 329 | (9Z,11R,12S,13S,15Z)-12,13-Epoxy-11-hydroxy-9,15-octadecadienoic acid | C18H30O4 | M+NH4 | 328.2476 | 6.3155 | HMDB0033505 | 5.4656 | 5.4678 | 5.5157 | 4.8582 | 4.7848 | 4.8542 |
| 330 | 3-(4-Isopropylphenyl)propanal | C12H16O | M+H | 177.1273 | 6.2945 | HMDB0036171 | 5.7717 | 5.7576 | 5.7995 | 4.6461 | 4.6672 | 4.6151 |
| 331 | Ichangic acid 17-beta-D-glucopyranoside | C32H44O15 | M+Na | 691.2607 | 6.2875 | HMDB0037590 | 6.4754 | 6.4516 | 6.4787 | 3.7281 | 3.7117 | 3.7174 |
| 332 | Lucidenic acid C | C27H40O7 | M+H | 477.2842 | 6.2666 | HMDB0037609 | 6.2982 | 6.2991 | 6.3304 | 3.0863 | 3.5787 | 3.6528 |
| 333 | Bisacurone epoxide | C15H24O4 | M+H | 269.1744 | 6.1545 | HMDB0038503 | 6.0487 | 6.0557 | 6.0629 | 5.2381 | 5.2069 | 5.202 |
| 334 | Glyceryl 5-hydroxydecanoate | C13H26O5 | M+Na | 285.1693 | 6.1335 | HMDB0032297 | 5.7311 | 5.729 | 5.7582 | 6.4606 | 6.4415 | 6.433 |
| 335 | Cuminaldehyde | C10H12O | M+H | 149.0961 | 6.1265 | HMDB0002214 | 4.8586 | 4.9207 | 4.9292 | 4.2675 | 4.2734 | 4.294 |
| 336 | L-Menthyl (R,S)-3-hydroxybutyrate | C14H26O3 | M+ACN+Na | 306.2059 | 6.0495 | HMDB0032370 | 5.0491 | 5.0229 | 5.0393 | 4.128 | 4.1116 | 4.1174 |
| 337 | L-Citronellol glucoside | C16H30O6 | M+CH3OH+H | 351.2386 | 5.9928 | HMDB0029850 | 4.9056 | 4.9674 | 4.9225 | 4.0393 | 4.023 | 4.0287 |
| 338 | 5-Megastigmen-7-yne-3,9-diol 9-glucoside | C19H30O7 | M+Na | 393.1879 | 5.7815 | HMDB0032841 | 3.817 | 4.0176 | 3.8272 | 5.2385 | 5.2092 | 5.233 |
| 339 | Physagulin C | C30H38O9 | M+ACN+H | 584.2841 | 5.6897 | HMDB0038535 | 5.7592 | 5.8015 | 5.8196 | 4.8781 | 4.8591 | 4.9222 |
| 340 | Lucidenic acid D1 | C27H34O7 | M+Na | 493.2214 | 5.4504 | HMDB0038199 | 3.9395 | 3.92 | 3.586 | 4.8659 | 4.8084 | 4.6722 |
| 341 | (3S,5R,6S,7E,9x)-7-Megastigmene-3,6,9-triol 9-glucoside | C19H34O8 | M+NH4 | 408.2587 | 5.1849 | LMFA13010057 | 3.4387 | 4.1172 | 3.5769 | 4.6767 | 4.7722 | 4.7998 |
| 342 | Arlatin | C15H22O4 | M+H | 267.1586 | 4.9602 | HMDB0035740 | 4.775 | 4.8452 | 4.8348 | 3.5649 | 3.604 | 3.2461 |
| 343 | (+)-Abscisic Acid | C15H20O4 | M+H | 265.1433 | 4.8768 | HMDB0035140 | 4.1884 | 4.4249 | 4.2986 | 5.0889 | 5.1212 | 5.0771 |
| 344 | Canesceol | C29H44O11 | M+H-H2O | 551.2794 | 4.8558 | HMDB0034084 | 5.7213 | 5.7607 | 5.7564 | 5.0207 | 5.0293 | 5.0308 |
| 345 | LysoPC(18:4(6Z,9Z,12Z,15Z)) | C26H46NO7P | M+NH4 | 533.3332 | 4.8412 | HMDB0010389 | 4.4201 | 4.4335 | 4.2521 | 5.5852 | 5.6046 | 5.587 |
| 346 | 2-Ethoxy-1-methyl-4-(1-methylethyl)benzene | C12H18O | M+ACN+H | 220.1695 | 4.8062 | HMDB0037272 | 6.6102 | 6.6029 | 6.6362 | 4.7358 | 4.6574 | 4.6848 |
| 347 | 4-Hydroxyproline galactoside | C11H19NO8 | M+H-2H2O | 258.097 | 4.5457 | HMDB0029354 | 3.777 | 3.8038 | 3.8021 | 4.7362 | 4.72 | 4.7481 |
| 348 | Dihydrocumambrin A | C17H24O5 | M+NH4 | 326.1958 | 4.5176 | HMDB0036489 | 4.8298 | 4.9129 | 4.8567 | 2.7972 | 2.7808 | 2.7866 |
| 349 | Gibberellin A95 | C19H22O5 | M+H-2H2O | 295.1326 | 4.4043 | HMDB0041350 | 3.4675 | 3.351 | 3.3493 | 4.2242 | 4.328 | 4.2347 |
| 350 | Gibberellin A39 | C20H26O8 | M+ACN+Na | 458.1805 | 4.2565 | HMDB0035045 | 5.1297 | 5.1558 | 5.1737 | 3.7841 | 3.9672 | 4.1751 |
| 351 | 3,8-Dihydroxy-6-methoxy-7(11)-eremophilen-12,8-olide | C16H24O5 | M+NH4 | 314.1959 | 4.1666 | HMDB0041551 | 4.9578 | 4.9603 | 4.9376 | 4.1347 | 4.1183 | 4.1241 |
| 352 | 12-Oxo-2,3-dinor-10,15-phytodienoic acid | C16H24O3 | M+NH4 | 282.2061 | 4.0043 | HMDB0032090 | 5.2649 | 5.348 | 5.3153 | 4.6859 | 4.6695 | 4.6753 |
| 353 | Deoxynivalenol | C15H20O6 | M+NH4 | 314.1594 | 3.7662 | HMDB0036156 | 4.7922 | 4.8226 | 4.7803 | 3.8452 | 4.052 | 3.944 |
| 354 | Neriantogenin | C23H32O4 | M+2Na-H | 417.1976 | 3.4499 | HMDB0030044 | 6.0126 | 6.0523 | 6.0627 | 4.3522 | 4.4212 | 4.3507 |
| 355 | (S)-Succinyldihydrolipoamide | C12H21NO4S2 | M+ACN+Na | 371.1084 | 2.9515 | HMDB0001177 | 3.1769 | 3.2037 | 3.4023 | 5.1492 | 5.153 | 5.1851 |
| 356 | Hydroxyoctanoic acid | C8H16O3 | 2M+K | 359.1809 | 2.8738 | HMDB0000711 | 2.9973 | 3.1578 | 2.1322 | 6.077 | 6.0477 | 6.0432 |
| 357 | Osmaronin | C11H17NO6 | M+CH3OH+H | 292.1388 | 1.9088 | HMDB0032769 | 3.2 | 3.2267 | 3.225 | 5.6368 | 5.6343 | 5.6486 |
| 358 | Dodecanedioic acid | C12H22O4 | 2M+NH4 | 478.3366 | 6.462 | LMFA01170009 | 4.3123 | 4.3391 | 4.7462 | 5.9263 | 5.8715 | 5.9111 |
| 359 | LysoPC(18:0) | C26H54NO7P | M+H | 524.3705 | 8.8159 | HMDB0011149 | 4.6451 | 4.4757 | 4.6125 | 5.6708 | 5.6504 | 5.6628 |
| 360 | Prostaglandin J2 | C20H30O4 | M+H-H2O | 317.2106 | 6.4895 | HMDB0247117 | 5.8105 | 5.8476 | 5.8382 | 4.8057 | 4.6799 | 4.7325 |
| 361 | 1,2-Di-(9Z,12Z,15Z-octadecatrienoyl)-3-(Galactosyl-alpha-1-6-Galactosyl-beta-1)-glycerol | C51H84O15 | M+Cl, M+FA-H, M-H | 981.5794 | 8.8496 | HMDB0011127 | 4.7275 | 4.7915 | 4.3697 | 6.0882 | 6.0743 | 6.1917 |
| 362 | Corchorifatty acid A | C18H28O4 | M-H, M+Cl, M-H2O-H | 307.1914 | 6.4348 | HMDB0032664 | 6.5727 | 6.6161 | 6.57 | 4.9609 | 4.9254 | 4.8782 |
| 363 | Citrusin D | C16H22O8 | M-H, M+Cl | 341.124 | 3.9154 | HMDB0039334 | 4.8191 | 4.759 | 4.7543 | 6.011 | 5.9717 | 5.9528 |
| 364 | Hydroxyisonobilin | C20H26O6 | M-H, M+K-2H | 361.1654 | 4.4046 | HMDB0034475 | 3.8964 | 4.0553 | 4.3276 | 5.0445 | 5.0522 | 5.1544 |
| 365 | Corchorifatty acid F | C18H32O5 | M-H, M+Cl | 327.2175 | 6.3206 | HMDB0035919 | 7.4083 | 7.4042 | 7.393 | 6.309 | 6.259 | 6.2213 |
| 366 | 13(S)-HpODE | C18H32O4 | M-H, M+Cl | 311.2227 | 6.5456 | HMDB0003871 | 7.1557 | 7.1284 | 7.1542 | 5.8976 | 5.873 | 5.8811 |
| 367 | LysoPC(18:1(9Z)) | C26H52NO7P | M+Cl, M+FA-H | 566.3464 | 6.8007 | HMDB0061701 | 4.2746 | 4.5686 | 4.3715 | 5.2033 | 5.1317 | 5.1202 |
| 368 | 7,8-Dihydrovomifoliol 9-[rhamnosyl-(1->6)-glucoside] | C25H42O12 | M-H2O-H, M-H | 515.2449 | 6.8315 | HMDB0029770 | 4.4876 | 4.8804 | 4.8847 | 6.2766 | 6.2031 | 6.2105 |
| 369 | LysoPC(18:2(9Z,12Z)) | C26H50NO7P | M+Cl, M+FA-H | 564.3305 | 6.7122 | HMDB0010386 | 5.4885 | 5.5846 | 5.4819 | 6.9955 | 6.9576 | 6.922 |
| 370 | PC(18:3(6Z,9Z,12Z)/18:3(6Z,9Z,12Z)) | C44H76NO8P | M+Cl, M+FA-H | 822.5297 | 7.4338 | LMGP01011654 | 4.8489 | 4.331 | 4.6336 | 6.4692 | 6.3678 | 6.3537 |
| 371 | PC(18:0/18:3(6Z,9Z,12Z)) | C44H82NO8P | M+Cl, M+FA-H | 828.5762 | 8.7874 | LMGP01011588 | 5.9787 | 4.9834 | 4.7165 | 7.0198 | 7.0591 | 7.0459 |
| 372 | Avenoleic acid | C18H32O3 | M-H2O-H, M+Cl | 277.2171 | 6.6466 | LMFA02000054 | 5.4258 | 5.4624 | 5.4933 | 6.1683 | 6.0966 | 6.0945 |
| 373 | Ginsenoside Rh6 | C36H62O11 | M+Cl, M+FA-H | 715.4256 | 6.2655 | HMDB0039436 | 3.2953 | 3.679 | 3.9884 | 4.6859 | 4.9027 | 4.7973 |
| 374 | 2-Hydroxyestrone sulfate | C18H22O6S | 2M+FA-H | 777.2311 | 2.7876 | HMDB0012622 | 6.7615 | 6.7503 | 6.6853 | 6.1567 | 6.0339 | 6.1447 |
| 375 | 13-Oxo-9,11-tridecadienoic acid | C13H20O3 | M+K-2H | 261.0881 | 2.8509 | LMFA01060202 | 6.3472 | 6.3394 | 6.3176 | 5.3642 | 5.2819 | 5.3669 |
| 376 | Portuloside A | C16H26O7 | M+Cl | 365.1351 | 3.1825 | HMDB0031382 | 3.8088 | 3.437 | 3.6437 | 5.4492 | 5.4313 | 5.4527 |
| 377 | Gibberellin A88 | C19H22O5 | M+FA-H | 375.1447 | 3.6002 | HMDB0039240 | 5.4081 | 5.4179 | 5.4036 | 4.1216 | 4.1339 | 3.8806 |
| 378 | Bakkenolide D | C21H28O6S | M-H2O-H | 389.1453 | 3.8967 | HMDB0034998 | 6.0168 | 5.9989 | 5.9668 | 5.3593 | 5.3601 | 5.3701 |
| 379 | Furfuryl pentanoate | C10H14O3 | M+FA-H | 227.0921 | 4.4416 | LMFA07010892 | 5.5192 | 5.4856 | 5.5072 | 4.7569 | 4.8154 | 4.773 |
| 380 | 11,13-Dihydrotaraxinic acid glucosyl ester | C21H30O9 | M+FA-H | 471.1869 | 4.8398 | HMDB0035867 | 3.3053 | 3.9625 | 3.3181 | 5.0997 | 5.2082 | 5.2126 |
| 381 | Tauro-b-muricholic acid | C26H45NO7S | M-H | 514.2883 | 5.3023 | LMST05040012 | 3.8767 | 3.741 | 4.2906 | 5.2591 | 5.2795 | 5.2448 |
| 382 | Musabalbisiane A | C23H28O12 | M-H2O-H | 477.14 | 5.8308 | HMDB0038680 | 5.8063 | 5.8115 | 5.8682 | 6.4987 | 6.531 | 6.5123 |
| 383 | Undecanoic acid | C11H22O2 | M+FA-H | 231.1598 | 5.9669 | LMFA01010011; | 4.0911 | 4.1665 | 4.1015 | 4.8385 | 4.8625 | 4.837 |
| 384 | Melledonol | C23H30O8 | M+Na-2H | 455.1689 | 6.0778 | HMDB0035884 | 4.0728 | 4.0105 | 4.0965 | 5.1854 | 5.2795 | 5.2505 |
| 385 | (x)-2-Heptanol glucoside | C13H26O6 | M-H2O-H | 259.155 | 6.1334 | HMDB0035028 | 4.8208 | 4.8394 | 4.8832 | 3.193 | 3.2049 | 3.1811 |
| 386 | Oleuroside | C25H32O13 | M-H2O-H | 521.1662 | 6.2303 | HMDB0035352 | 3.0874 | 3.0869 | 3.1002 | 5.4555 | 5.466 | 5.4267 |
| 387 | 1-(3-Methyl-2-butenoyl)-6-apiosylglucose | C16H26O11 | 2M+FA-H | 833.2896 | 6.258 | HMDB0039952 | 4.2387 | 4.2381 | 4.2515 | 5.0302 | 5.1466 | 5.0194 |
| 388 | (R)-2-Hydroxycaprylic acid | C8H16O3 | M-H | 159.1018 | 6.2793 | HMDB0002264 | 2.9392 | 2.9205 | 2.6139 | 4.2246 | 4.2253 | 4.1723 |
| 389 | (-)-11-Hydroxy-9,15,16-trioxooctadecanoic acid | C18H30O6 | M-H2O-H | 323.1863 | 6.3557 | HMDB0038940 | 5.9837 | 5.9834 | 5.9671 | 4.5499 | 4.3749 | 4.4138 |
| 390 | 10,11-dihydro-20-dihydroxy-LTB4 | C20H34O6 | M-H2O-H | 351.2176 | 6.4693 | HMDB0012502 | 5.38 | 5.3931 | 5.3942 | 4.7071 | 4.7047 | 4.7442 |
| 391 | 24-Oxo-1alpha,25-dihydroxyvitamin D3 | C27H42O4 | M+Na-2H | 451.2854 | 6.5037 | HMDB0060128 | 2.7554 | 2.7548 | 2.8801 | 4.448 | 4.3795 | 4.4101 |
| 392 | Xi-7-Hydroxyhexadecanedioic acid | C16H30O5 | 2M+FA-H | 649.4113 | 6.6014 | HMDB0037830 | 3.4871 | 3.6133 | 3.4999 | 5.3163 | 5.2634 | 5.2655 |
| 393 | Barringtogenol C | C30H50O5 | M+FA-H | 535.364 | 6.6078 | HMDB0034525 | 5.7994 | 5.7565 | 5.7167 | 4.5841 | 4.6199 | 4.7489 |
| 394 | DG(13:0/15:0/0:0) | C31H60O5 | M+FA-H | 557.442 | 6.6078 | HMDB0093193 | 3.374 | 3.3734 | 3.3868 | 5.1041 | 5.0059 | 5.0103 |
| 395 | Calamendiol | C15H26O2 | M+FA-H | 283.1914 | 6.6466 | HMDB0034673 | 5.2559 | 5.2989 | 5.3675 | 3.9253 | 3.9062 | 4.0145 |
| 396 | PA(20:4(5Z,8Z,11Z,14Z)/20:5(5Z,8Z,11Z,14Z,17Z)) | C43H67O8P | M+Cl | 777.4211 | 6.6466 | HMDB0115161 | 5.6181 | 5.7051 | 5.7308 | 4.4269 | 4.3793 | 4.2073 |
| 397 | Desglucodesrhamnoparillin | C39H64O13 | M+Cl | 775.4063 | 6.6662 | HMDB0033493 | 5.5622 | 5.6349 | 5.6272 | 4.1272 | 4.2823 | 3.6606 |
| 398 | Erinacine E | C25H36O6 | M+Na-2H | 453.2258 | 6.6959 | HMDB0029656 | 4.4415 | 4.3489 | 4.3637 | 5.3384 | 5.3779 | 5.331 |
| 399 | 1-(9Z,12Z,15Z-octadecatrienoyl)-glycero-3-phosphate | C21H37O7P | M-H | 431.2202 | 6.7181 | HMDB0062320 | 5.8823 | 5.9284 | 5.9497 | 5.2703 | 5.2167 | 5.1234 |
| 400 | LysoPE(0:0/18:3(9Z,12Z,15Z)) | C23H42NO7P | M+FA-H | 520.2673 | 6.7295 | HMDB0011479 | 4.2033 | 4.0456 | 4.0631 | 5.473 | 5.4503 | 5.3862 |
| 401 | 8-Hydroxy-6-heneicosanone | C21H42O2 | M+FA-H | 371.3164 | 6.7894 | HMDB0035669 | 5.559 | 5.59 | 5.5986 | 5.0158 | 4.837 | 4.8882 |
| 402 | PE(16:0/0:0) | C21H44NO7P | M-H | 452.2782 | 6.8007 | HMDB0011503 | 5.5738 | 5.6312 | 5.5714 | 6.5111 | 6.4449 | 6.4445 |
| 403 | LysoPA(0:0/18:2(9Z,12Z)) | C21H39O7P | M-H | 433.2358 | 6.8076 | HMDB0007852 | 6.4172 | 6.543 | 6.503 | 5.5938 | 5.4918 | 5.4739 |
| 404 | 3-hydroxypentadecanoic acid | C15H30O3 | M-H | 257.2121 | 6.814 | HMDB0061657 | 4.9438 | 4.9717 | 4.9496 | 4.38 | 4.3162 | 4.2611 |
| 405 | 14-oxo-DoHE(1-) | C22H30O3 | 2M+Hac-H | 743.4508 | 6.827 | HMDB0062685 | 6.4224 | 6.6655 | 6.6353 | 4.7627 | 3.9331 | 3.1704 |
| 406 | 1-(9Z-hexadecenoyl)-glycero-3-phosphate | C19H37O7P | M-H | 407.2202 | 6.8518 | HMDB0062323 | 5.6056 | 5.7416 | 5.7463 | 3.7681 | 2.8217 | 2.9902 |
| 407 | Avocadene 1-acetate | C19H36O4 | M+FA-H | 373.2593 | 6.906 | HMDB0031043 | 3.992 | 4.1044 | 4.0242 | 5.1375 | 4.9862 | 4.8991 |
| 408 | 1-(11Z,14Z-eicosadienoyl)-glycero-3-phosphate | C23H43O7P | M+FA-H | 507.2725 | 6.9129 | LMGP10050027 | 5.4726 | 5.3689 | 5.3058 | 6.8288 | 6.8766 | 6.8492 |
| 409 | Tussilagonone | C21H30O3 | 2M+Hac-H | 719.452 | 6.9557 | HMDB0041585 | 5.7587 | 5.9661 | 5.9636 | 4.347 | 3.7263 | 3.4827 |
| 410 | LysoPA(0:0/18:0) | C21H43O7P | M-H | 437.2672 | 7.0851 | HMDB0007850 | 5.287 | 5.3765 | 5.3875 | 4.6622 | 4.7333 | 4.5948 |
| 411 | PC(15:0/18:2(9Z,12Z)) | C41H78NO8P | M-H | 742.5394 | 8.6089 | HMDB0007940 | 3.7483 | 3.7478 | 3.7611 | 6.2815 | 6.3105 | 6.2445 |
| 412 | PC(18:1(11Z)/18:3(6Z,9Z,12Z)) | C44H80NO8P | M+FA-H | 826.5608 | 8.8565 | HMDB0008073 | 5.3856 | 5.222 | 4.9842 | 6.4149 | 6.4343 | 6.4063 |
| 413 | PE(16:0/18:3(6Z,9Z,12Z)) | C39H72NO8P | M-H | 712.493 | 8.8634 | LMGP02011224 | 4.2478 | 4.2261 | 4.2199 | 5.9017 | 5.9061 | 5.8685 |
| 414 | GPEtn(18:2/18:2) | C41H74NO8P | M-H | 738.5083 | 8.8634 | HMDB0011442 | 5.2964 | 5.2347 | 5.0276 | 6.4415 | 6.4677 | 6.4118 |
| 415 | 1-(11Z-eicosenoyl)-glycero-3-phosphate | C23H45O7P | M+FA-H | 509.2885 | 8.8368 | HMDB0062305 | 4.1284 | 3.9583 | 3.3009 | 5.5384 | 5.5405 | 5.4823 |
| 416 | PI(18:0/18:2(9Z,12Z)) | C45H83O13P | M-H | 861.5496 | 8.8092 | LMGP06010956 | 6.4575 | 6.2425 | 6.2328 | 7.0876 | 7.1314 | 7.093 |
| 417 | PC(16:0/18:2(9Z,12Z)) | C42H80NO8P | M+FA-H | 802.5604 | 8.8092 | LMGP01010594 | 5.706 | 5.9805 | 5.2952 | 7.4544 | 7.4772 | 7.4235 |
| 418 | PA(18:0/18:2(9Z,12Z)) | C39H73O8P | M-H | 699.4973 | 8.027 | LMGP10010036 | 6.4904 | 6.5281 | 6.5346 | 5.74 | 5.7146 | 5.758 |
| 419 | PA(16:0/18:1(11Z)) | C37H71O8P | M-H | 673.4809 | 8.0159 | LMGP10010007 | 5.9205 | 5.9405 | 5.9331 | 4.6824 | 4.4496 | 4.6171 |
| 420 | PG(16:1(9Z)/18:2(9Z,12Z)) | C40H73O10P | M-H | 743.4868 | 7.838 | LMGP04010901; | 6.5991 | 6.5639 | 6.604 | 7.347 | 7.28 | 7.3051 |
| 421 | PG(18:1(11Z)/18:3(9Z,12Z,15Z)) | C42H75O10P | M-H | 769.5025 | 7.8235 | HMDB0010622 | 3.6279 | 4.0025 | 4.1329 | 6.4712 | 6.4039 | 6.4196 |
| 422 | PA(18:0/18:3(6Z,9Z,12Z)) | C39H71O8P | M-H | 697.4815 | 7.729 | LMGP10010313 | 6.5952 | 6.6521 | 6.6245 | 5.5504 | 5.4332 | 5.4331 |
| 423 | PG(22:6(4Z,7Z,10Z,13Z,16Z,19Z)/22:6(4Z,7Z,10Z,13Z,16Z,19Z)) | C50H75O10P | M-H | 865.509 | 7.5116 | LMGP04010977 | 6.4688 | 6.4636 | 6.4809 | 5.2346 | 5.0337 | 5.0064 |
| 424 | PG(20:4(5Z,8Z,11Z,14Z)/22:6(4Z,7Z,10Z,13Z,16Z,19Z)) | C48H75O10P | M-H | 841.5105 | 7.4975 | HMDB0116613 | 3.1705 | 3.4639 | 3.6033 | 6.0668 | 6.0599 | 6.053 |
| 425 | 1,2-Di-O-palmitoyl-3-O-(6-sulfoquinovopyranosyl)glycerol | C41H78O12S | M+Na-2H | 815.5003 | 7.4975 | HMDB0041202 | 4.9439 | 4.1363 | 4.3678 | 7.3032 | 7.3032 | 7.3165 |
| 426 | PE(18:1(11Z)/18:3(6Z,9Z,12Z)) | C41H74NO8P | M-H | 738.5083 | 7.3733 | HMDB0009028 | 6.4271 | 6.4251 | 6.4053 | 7.17 | 7.1015 | 7.087 |
| 427 | 1-Stearoylglycerophosphoglycerol | C24H49O9P | M-H | 511.3038 | 7.3334 | HMDB0061697 | 5.1614 | 4.9527 | 4.9966 | 5.8224 | 5.8076 | 5.7797 |
| 428 | 3-Oxo-12,18-ursadien-28-oic acid | C30H44O3 | M+K-2H | 489.2774 | 7.2759 | HMDB0037065 | 5.6325 | 5.6318 | 5.6686 | 4.9006 | 4.8347 | 4.9423 |
| 429 | PS(20:0/22:6(4Z,7Z,10Z,13Z,16Z,19Z)) | C48H82NO10P | M+Cl | 898.5455 | 7.1697 | LMGP03010861 | 4.0249 | 3.9285 | 3.9311 | 5.971 | 5.8985 | 5.9037 |
| 430 | Goyaglycoside c | C38H62O9 | M+Cl | 697.4076 | 7.0104 | HMDB0038349 | 5.6445 | 5.7939 | 5.7213 | 4.2127 | 3.8541 | 3.618 |
| 431 | PA(20:4(5Z,8Z,11Z,14Z)/22:2(13Z,16Z)) | C45H77O8P | M+Na-2H | 797.5061 | 6.9375 | HMDB0115164 | 6.0031 | 6.1957 | 6.1074 | 4.875 | 4.4938 | 4.1425 |
| 432 | PG(a-13:0/a-13:0) | C32H63O10P | M+Na-2H | 659.3932 | 6.934 | HMDB0116636 | 4.8088 | 5.066 | 4.7931 | 2.9738 | 2.9696 | 2.9619 |
| 433 | Stearic acid | C18H36O2 | M+K-2H | 321.2196 | 6.8873 | LMFA01010018 | 4.9212 | 4.9736 | 5.0626 | 4.2531 | 4.3989 | 4.3711 |
| 434 | Hydratopyrrhoxanthinol | C37H48O6 | M-H | 587.3356 | 6.8833 | HMDB0036842 | 5.1731 | 5.4419 | 5.4349 | 3.6696 | 3.5298 | 3.5221 |
| 435 | Physangulide | C28H42O9 | M+K-2H | 559.235 | 6.8315 | HMDB0037381 | 3.7995 | 4.1481 | 3.8434 | 5.7306 | 5.6692 | 5.6793 |
| 436 | PG(a-13:0/18:2(9Z,11Z)) | C37H69O10P | M+Na-2H | 725.4419 | 6.8007 | HMDB0116637 | 6.2101 | 6.353 | 6.2922 | 5.1905 | 5.0126 | 4.9314 |
| 437 | 1-(8Z,11Z,14Z-eicosatrienoyl)-glycero-3-phosphate | C23H41O7P | M+FA-H | 505.2573 | 6.7432 | HMDB0062313 | 4.7146 | 4.614 | 4.5142 | 6.8894 | 6.9535 | 6.9105 |
| 438 | Methyl octynecarboxylate | C10H16O2 | 2M+Hac-H | 395.2436 | 6.7295 | LMFA07010964 | 4.689 | 4.612 | 4.4226 | 5.6952 | 5.5558 | 5.5173 |
| 439 | 3,4-Dimethyl-5-pentyl-2-furanundecanoic acid | C22H38O3 | M+FA-H | 395.28 | 6.6466 | HMDB0031126 | 5.1393 | 5.1435 | 5.17 | 4.3559 | 4.236 | 4.2711 |
| 440 | Galactosylglycerol | C9H18O8 | M-H | 253.0927 | 6.6466 | HMDB0006790 | 2.9258 | 2.9253 | 2.9387 | 4.7927 | 4.7127 | 4.6877 |
| 441 | PGP(18:3(6Z,9Z,12Z)/22:5(4Z,7Z,10Z,13Z,16Z)) | C46H76O13P2 | M+Na-2H | 919.4463 | 6.6338 | HMDB0013574 | 3.8588 | 3.8582 | 3.8716 | 5.579 | 5.2155 | 5.219 |
| 442 | (+/-)-(E)-13-Hydroxy-10-oxo-11-octadecenoic acid | C18H32O4 | M+Cl | 347.1992 | 6.6338 | HMDB0040900 | 5.2593 | 5.3151 | 5.3581 | 3.8735 | 3.8693 | 3.8615 |
| 443 | (1(10)E,4a,5E)-1(10),5-Germacradiene-12-acetoxy-4,11-diol | C17H28O4 | M-H | 295.1914 | 6.6269 | HMDB0038798 | 5.053 | 5.081 | 5.0365 | 3.8697 | 3.679 | 3.7845 |
| 444 | 19-Hydroxycinnzeylanol 19-glucoside | C26H42O13 | M+FA-H | 607.2565 | 6.5955 | HMDB0036856 | 3.0493 | 3.0488 | 3.0622 | 6.2992 | 6.2578 | 6.2815 |
| 445 | 7-Ketodeoxycholic acid | C24H38O5 | M-H | 405.2644 | 6.5741 | HMDB0000391 | 5.3351 | 5.3617 | 5.3661 | 4.4936 | 4.3924 | 4.4206 |
| 446 | Withaperuvin H | C30H42O9S | M+FA-H | 623.2513 | 6.5579 | HMDB0034061 | 4.036 | 4.0354 | 4.0488 | 5.7538 | 5.752 | 5.7309 |
| 447 | Lucyoside N | C36H58O10 | M-H | 649.3975 | 6.5111 | HMDB0041040 | 4.3654 | 4.517 | 4.4643 | 5.2418 | 5.166 | 5.1283 |
| 448 | Corchorifatty acid D | C18H28O4 | M-H | 307.1914 | 6.5037 | HMDB0033243 | 6.5437 | 6.5115 | 6.5522 | 5.3925 | 5.3626 | 5.3939 |
| 449 | LysoPE(0:0/20:3(11Z,14Z,17Z)) | C25H46NO7P | M+FA-H | 548.302 | 6.483 | HMDB0011484 | 4.6196 | 4.619 | 4.6324 | 5.2985 | 5.3264 | 5.2654 |
| 450 | Glycosides | C29H44O12 | M+K-2H | 621.2358 | 6.4555 | HMDB0031448 | 4.0185 | 4.018 | 4.0314 | 5.4084 | 5.4539 | 5.4317 |
| 451 | Hemsloside Ma 2 | C52H82O22 | M-H2O-H | 1039.512 | 6.4136 | HMDB0041022 | 4.2196 | 4.219 | 4.4913 | 6.0492 | 5.9944 | 5.9982 |
| 452 | 1-Acetoxy-2-hydroxy-16-heptadecyn-4-one | C19H32O4 | M+FA-H | 369.2281 | 6.3967 | LMFA05000595 | 5.551 | 5.5984 | 5.6572 | 4.6993 | 4.7016 | 4.6184 |
| 453 | 5,8,12-Trihydroxy-9-octadecenoic acid | C18H34O5 | M+Cl | 365.2096 | 6.3694 | LMFA01050543 | 5.5578 | 5.5518 | 5.5197 | 3.8691 | 3.8816 | 4.0508 |
| 454 | Gingerglycolipid A | C33H56O14 | M-H2O-H | 657.3497 | 6.2862 | HMDB0041093 | 5.9222 | 5.9216 | 5.9377 | 3.6856 | 2.8276 | 3.1727 |
| 455 | Asiaticoside B | C48H78O20 | M+FA-H | 1019.508 | 6.2097 | HMDB0029892 | 6.6501 | 6.6545 | 6.6711 | 5.7823 | 5.8546 | 5.8072 |
| 456 | Dihydrojasmonic acid | C12H20O3 | M-H | 211.1335 | 6.1746 | HMDB0033601 | 5.3232 | 5.3403 | 5.3568 | 2.7964 | 3.3606 | 3.0123 |
| 457 | Undecylenic acid | C11H20O2 | M+FA-H | 229.1442 | 6.1608 | HMDB0033724 | 5.0459 | 5.0789 | 5.1029 | 4.4003 | 4.4066 | 4.3709 |
| 458 | Cis-5-Octenoic acid | C8H14O2 | 2M+Hac-H | 343.2124 | 6.1334 | HMDB0032207 | 6.5307 | 6.5092 | 6.5402 | 5.2476 | 5.2828 | 5.2293 |
| 459 | N-[(3a,5b,7b)-7-hydroxy-24-oxo-3-(sulfooxy)cholan-24-yl]-Glycine | C26H43NO8S | M-H | 528.2661 | 5.9062 | HMDB0002409 | 4.5316 | 4.6161 | 4.7449 | 5.247 | 5.2782 | 5.2553 |
| 460 | Isobutyl 2-furanpropionate | C11H16O3 | M+FA-H | 241.1079 | 5.8875 | LMFA07010915 | 5.4824 | 5.4991 | 5.5405 | 4.1304 | 4.2296 | 4.3242 |
| 461 | (1alpha,2alpha,4betaH,6alpha,8R)-p-Menthane-2,6,8,9-tetrol | C10H20O4 | M-H | 203.1283 | 5.3023 | HMDB0039053 | 3.6642 | 3.7739 | 3.7976 | 4.3507 | 4.4202 | 4.4042 |
| 462 | Methyl helianthenoate A glucoside | C19H24O8 | M+Na-2H | 401.124 | 4.7977 | HMDB0040899 | 5.3567 | 5.3107 | 5.3474 | 1.3029 | 1.299 | 1.2916 |
| 463 | Demethyloleuropein | C24H30O13 | M-H2O-H | 507.1506 | 4.7158 | HMDB0036121 | 3.5865 | 3.3072 | 3.6044 | 5.3936 | 5.5244 | 5.5048 |
| 464 | 2-Octenedioic acid | C8H12O4 | 2M+Hac-H | 403.1609 | 3.9842 | HMDB0000341 | 4.2869 | 4.1723 | 4.0026 | 4.9558 | 4.9641 | 4.9553 |
| 465 | 1-(sn-Glycero-3-phospho)-1D-myo-inositol | C9H19O11P | M-H | 333.0592 | 0.921 | HMDB0011649 | 5.8832 | 5.8952 | 5.8839 | 4.694 | 4.5777 | 4.641 |
| 466 | Trolamine | C6H15NO3 | M+H | 150.1124 | 8.7179 | HMDB0032538 | 6.6065 | 6.6152 | 6.6573 | 5.982 | 5.979 | 6.0002 |
| 467 | Phosphocholine | C5H14NO4P | M+H | 184.0731 | 6.6921 | HMDB0001565 | 5.0339 | 4.6609 | 4.5382 | 5.8744 | 5.8662 | 5.8895 |
| 468 | Eicosapentaenoyl Ethanolamide | C22H35NO2 | M+H-2H2O | 310.2523 | 6.3645 | HMDB0013649 | 5.3449 | 5.405 | 5.4028 | 4.4593 | 4.3132 | 4.3017 |

**Table S2**. List of 107 different nonvolatile metabolites before and after jujube leaf processing

| Metabolite | class | VIP_pred_OPLS-DA | VIP_PLS-DA | FC(D2/D1) | P_value | FDR | D2_1 | D2_2 | D2_3 | D1_1 | D1_2 | D1_3 |
| --- | --- | --- | --- | --- | --- | --- | --- | --- | --- | --- | --- | --- |
| 8-Methoxyeriodictyol | Phenol and phenol ether | 1.752594 | 1.752541 | 1.540615 | 2.94E-08 | 1.64E-06 | 4.9943 | 4.9956 | 5.0306 | 3.2326 | 3.2594 | 3.2577 |
| Artocommunol CC |  | 2.405507 | 2.404947 | 0.465393 | 5.81E-05 | 0.000202 | 3.2385 | 2.5954 | 2.8605 | 6.2194 | 6.2321 | 6.229 |
| Lecanoric acid |  | 1.960414 | 1.960406 | 1.623513 | 1.41E-06 | 1.56E-05 | 5.7247 | 5.7426 | 5.7243 | 3.4695 | 3.4963 | 3.6252 |
| Oleuroside |  | 1.961605 | 1.961402 | 1.76229 | 4.78E-09 | 6.49E-07 | 5.4555 | 5.466 | 5.4267 | 3.0874 | 3.0869 | 3.1002 |
| N,N-Didesmethyltramadol |  | 1.778548 | 1.77865 | 1.539894 | 0.000131 | 0.000364 | 5.2052 | 5.2057 | 5.221 | 3.1715 | 3.6064 | 3.3746 |
| 6-Hydroxydaidzein 4'-glucoside | Flavonoids | 2.767318 | 2.767001 | 14.92285 | 8.27E-10 | 2.70E-07 | 5.0218 | 5.0346 | 5.0314 | 0.3181 | 0.368 | 0.3248 |
| Proanthocyanidin A5' |  | 1.964255 | 1.964052 | 1.748658 | 1.38E-05 | 7.80E-05 | 5.5138 | 5.5426 | 5.5582 | 3.3501 | 3.0687 | 3.0821 |
| DIHYDROROBINETIN |  | 1.959079 | 1.959378 | 1.663685 | 0.000277 | 0.000647 | 5.6062 | 5.5294 | 5.6042 | 2.989 | 3.576 | 3.4967 |
| Epiafzelechin-(4b->8)-epicatechin 3,3'-digallate |  | 2.092522 | 2.09251 | 1.71084 | 8.98E-09 | 8.38E-07 | 6.0515 | 6.0067 | 6.0274 | 3.5067 | 3.5335 | 3.5318 |
| Epigallocatechin 3-O-(4-hydroxybenzoate) |  | 2.682571 | 2.682298 | 5.979084 | 6.62E-05 | 0.00022 | 5.0058 | 4.9541 | 4.963 | 1.3039 | 0.6666 | 0.5251 |
| 3,3'-Digalloylprocyanidin B2 |  | 1.874556 | 1.874545 | 1.501498 | 8.94E-09 | 8.38E-07 | 6.0334 | 6.0047 | 6.0084 | 3.9883 | 4.0151 | 4.0133 |
| Hydroxyrepaglinide | Furan, indole, pyridine and other organic heterocyclic compounds | 2.951289 | 2.951347 | 0.115662 | 1.00E-06 | 1.32E-05 | 0.5552 | 0.8537 | 0.5475 | 5.6294 | 5.6452 | 5.6398 |
| 3-keto-Digoxigenin |  | 2.224799 | 2.224697 | 0.484529 | 5.26E-08 | 2.29E-06 | 2.6939 | 2.662 | 2.6316 | 5.487 | 5.5325 | 5.4618 |
| 6-Hydroxyetodolac |  | 1.850725 | 1.850655 | 1.612163 | 0.000412 | 0.00094 | 5.6242 | 5.6575 | 5.5788 | 3.8772 | 3.2837 | 3.2971 |
| Adrenochrome |  | 1.889347 | 1.889166 | 1.732107 | 2.15E-06 | 2.38E-05 | 5.0739 | 5.2169 | 5.2474 | 2.9854 | 2.9849 | 2.9982 |
| L-Tryptophanamide |  | 2.234604 | 2.234478 | 2.687566 | 0.01478 | 0.01924 | 5.0628 | 5.0623 | 5.0485 | 2.7086 | 0.3362 | 2.601 |
| 5-(2'-Carboxyethyl)-4,6-Dihydroxypicolinate |  | 1.996545 | 1.996306 | 1.886108 | 1.60E-09 | 3.73E-07 | 5.2071 | 5.1822 | 5.2101 | 2.7533 | 2.7527 | 2.7661 |
| Tetrahydrobiopterin |  | 2.972282 | 2.972176 | 0.099644 | 7.70E-11 | 1.86E-07 | 0.5658 | 0.5539 | 0.5581 | 5.5961 | 5.6122 | 5.627 |
| GEMIFLOXACIN |  | 1.99896 | 1.998822 | 1.705792 | 1.19E-05 | 6.44E-05 | 5.5539 | 5.5135 | 5.5442 | 3.4163 | 3.1613 | 3.1595 |
| 1-Pyrimidinylpiperazine |  | 2.085728 | 2.085768 | 1.864155 | 3.58E-05 | 0.00014 | 5.3779 | 5.3943 | 5.4071 | 2.6905 | 3.1174 | 2.8702 |
| Methyl helianthenoate A glucoside | Lipids and lipid-like molecules | 2.567901 | 2.567603 | 0.243162 | 9.70E-10 | 2.95E-07 | 1.3029 | 1.299 | 1.2916 | 5.3567 | 5.3107 | 5.3474 |
| (2E,11Z)-5-[5-(Methylthio)-4-penten-2-ynyl]-2-furanacrolein |  | 1.675364 | 1.675377 | 1.571886 | 1.59E-06 | 1.69E-05 | 4.4279 | 4.3743 | 4.449 | 2.7695 | 2.866 | 2.7945 |
| (S)-Succinyldihydrolipoamide |  | 1.820693 | 1.820723 | 1.58295 | 1.22E-05 | 6.59E-05 | 5.1492 | 5.153 | 5.1851 | 3.1769 | 3.2037 | 3.4023 |
| Osmaronin |  | 2.058135 | 2.058084 | 1.753186 | 1.55E-09 | 3.71E-07 | 5.6368 | 5.6343 | 5.6486 | 3.2 | 3.2267 | 3.225 |
| 1,2-Di-O-palmitoyl-3-O-(6-sulfoquinovopyranosyl)glycerol |  | 2.131963 | 2.13188 | 1.630158 | 0.000299 | 0.000736 | 7.3032 | 7.3032 | 7.3165 | 4.9439 | 4.1363 | 4.3678 |
| Galactosylglycerol |  | 1.713978 | 1.713779 | 1.614676 | 5.93E-07 | 1.02E-05 | 4.7927 | 4.7127 | 4.6877 | 2.9258 | 2.9253 | 2.9387 |
| Neoacrimarine F |  | 2.070024 | 2.069972 | 0.495369 | 2.27E-07 | 5.17E-06 | 2.416 | 2.3997 | 2.4054 | 4.8728 | 4.909 | 4.7942 |
| PC(15:0/18:2(9Z,12Z)) |  | 2.03044 | 2.030242 | 1.673507 | 2.16E-08 | 1.47E-06 | 6.2815 | 6.3105 | 6.2445 | 3.7483 | 3.7478 | 3.7611 |
| PG(18:1(11Z)/18:3(9Z,12Z,15Z)) |  | 2.016725 | 2.016627 | 1.640398 | 8.02E-05 | 0.000269 | 6.4712 | 6.4039 | 6.4196 | 3.6279 | 4.0025 | 4.1329 |
| PG(20:4(5Z,8Z,11Z,14Z)/22:6(4Z,7Z,10Z,13Z,16Z,19Z)) |  | 2.07377 | 2.073644 | 1.775564 | 3.19E-05 | 0.000133 | 6.0668 | 6.0599 | 6.053 | 3.1705 | 3.4639 | 3.6033 |
| PC(16:0/18:3(9Z,12Z,15Z)) |  | 2.431283 | 2.431163 | 1.722175 | 4.69E-06 | 3.45E-05 | 8.0993 | 7.9947 | 8.1365 | 4.8732 | 4.6025 | 4.5953 |
| 1-Linoleoylglycerophosphocholine |  | 2.056882 | 2.05691 | 1.662835 | 1.70E-06 | 1.78E-05 | 6.0829 | 6.047 | 6.0984 | 3.587 | 3.6138 | 3.7603 |
| 19-Hydroxycinnzeylanol 19-glucoside |  | 2.294587 | 2.294314 | 2.056993 | 1.47E-09 | 3.58E-07 | 6.2992 | 6.2578 | 6.2815 | 3.0493 | 3.0488 | 3.0622 |
| Demethyloleuropein |  | 1.790224 | 1.790152 | 1.564447 | 4.62E-05 | 0.000176 | 5.3936 | 5.5244 | 5.5048 | 3.5865 | 3.3072 | 3.6044 |
| Ginsenoside F1 |  | 1.993285 | 1.993277 | 1.606514 | 1.64E-08 | 1.20E-06 | 6.0385 | 5.9916 | 6.0253 | 3.7285 | 3.7553 | 3.7536 |
| 24-Oxo-1alpha,25-dihydroxyvitamin D3 |  | 1.622596 | 1.622425 | 1.577762 | 3.96E-06 | 3.49E-05 | 4.448 | 4.3795 | 4.4101 | 2.7554 | 2.7548 | 2.8801 |
| PE(18:2/0:0) |  | 1.917641 | 1.917394 | 1.6613 | 1.21E-05 | 7.07E-05 | 5.6774 | 5.6796 | 5.6679 | 3.324 | 3.5858 | 3.3368 |
| Chorismic acid | Organic acids and derivatives | 1.985512 | 1.98529 | 1.727875 | 7.55E-07 | 1.21E-05 | 5.7264 | 5.7438 | 5.7487 | 3.4122 | 3.2709 | 3.2843 |
| 6-Hydroxynicotinic acid |  | 1.627077 | 1.626935 | 1.546263 | 0.000735 | 0.001497 | 4.6937 | 4.6734 | 4.7208 | 2.7623 | 3.3686 | 2.9809 |
| Hydroxyoctanoic acid |  | 2.378033 | 2.377742 | 2.192614 | 0.000495 | 0.00103 | 6.077 | 6.0477 | 6.0432 | 2.9973 | 3.1578 | 2.1322 |
| Actinidic acid |  | 2.287172 | 2.287245 | 1.963426 | 1.80E-05 | 8.67E-05 | 6.1928 | 6.0963 | 6.071 | 2.9855 | 3.3565 | 3.0105 |
| (Z)-3-(1-Formyl-1-propenyl)pentanedioic acid |  | 1.892788 | 1.892829 | 1.647096 | 4.83E-05 | 0.000177 | 5.2506 | 5.2453 | 5.2429 | 3.0293 | 3.3983 | 3.1286 |
| (S)-Spinacine |  | 1.717033 | 1.71703 | 1.69503 | 0.000693 | 0.001427 | 4.4738 | 4.5198 | 4.5117 | 3.0443 | 2.4545 | 2.4678 |
| 3-Furoic acid |  | 1.708047 | 1.707862 | 1.607956 | 8.50E-09 | 8.14E-07 | 4.7091 | 4.7359 | 4.7419 | 2.9372 | 2.9366 | 2.95 |
| Pantetheine |  | 1.780624 | 1.780403 | 1.70809 | 2.91E-08 | 1.75E-06 | 4.6785 | 4.6653 | 4.7173 | 2.74 | 2.7395 | 2.7528 |
| Neuromedin B (1-3) |  | 1.713517 | 1.713558 | 1.588112 | 1.84E-06 | 1.88E-05 | 4.5501 | 4.5285 | 4.5465 | 2.7895 | 2.8678 | 2.924 |
| Gamma-Glutamyl-S-methylcysteine sulfoxide |  | 1.58329 | 1.583372 | 1.517279 | 6.90E-06 | 4.51E-05 | 4.2563 | 4.2282 | 4.1602 | 2.7278 | 2.7546 | 2.8513 |
| Oxidized glutathione |  | 2.596395 | 2.596499 | 3.320505 | 7.67E-07 | 1.09E-05 | 5.6644 | 5.472 | 5.4284 | 1.646 | 1.6722 | 1.6705 |
| Gamma-Glutamyl-beta-aminopropiononitrile |  | 1.966055 | 1.966817 | 1.842913 | 0.00584 | 0.008336 | 5.1431 | 5.1747 | 5.169 | 1.9262 | 3.3216 | 3.1566 |
| 3-Methylindolepyruvate |  | 1.841597 | 1.841542 | 1.73447 | 1.30E-08 | 1.06E-06 | 4.5763 | 4.5981 | 4.5638 | 2.6223 | 2.6491 | 2.6473 |
| HAEMATOMMIC ACID |  | 1.704818 | 1.704467 | 1.560771 | 0.000254 | 0.000646 | 5.0283 | 4.9996 | 5.0352 | 3.237 | 3.4606 | 2.9535 |
| 3'-(2''-Galloylglucosyl)-phloroacetophenone |  | 1.656464 | 1.656319 | 1.520247 | 2.45E-06 | 2.59E-05 | 4.8337 | 4.96 | 4.9616 | 3.2309 | 3.2303 | 3.2437 |
| 4-Hydroxymethylsalicylate |  | 1.81658 | 1.816653 | 1.523388 | 2.95E-06 | 2.53E-05 | 5.5335 | 5.4571 | 5.52 | 3.5304 | 3.6815 | 3.6281 |
| PPOH |  | 2.125961 | 2.125644 | 1.78043 | 0.00023 | 0.000559 | 5.9688 | 5.9885 | 5.948 | 3.759 | 3.2205 | 3.0761 |
| 3,4,5-trihydroxy-6-[(2-oxo-2H-chromen-5-yl)oxy]oxane-2-carboxylic acid |  | 1.821665 | 1.8214 | 1.67351 | 5.72E-07 | 9.98E-06 | 5.107 | 4.9869 | 5.0694 | 3.016 | 3.0154 | 3.0288 |
| 3,4,5-trihydroxy-6-{3-[2-(3-hydroxyphenyl)ethyl]-5-methoxyphenoxy}oxane-2-carboxylic acid |  | 2.250922 | 2.251154 | 0.389628 | 0.000534 | 0.001159 | 2.2324 | 1.4065 | 2.4245 | 5.1907 | 5.1992 | 5.1703 |
| 6-{4-[3-(3,7-dimethylocta-2,6-dien-1-yl)-7-hydroxy-4-oxo-4H-chromen-2-yl]-3-hydroxyphenoxy}-3,4,5-trihydroxyoxane-2-carboxylic acid |  | 1.877507 | 1.877299 | 1.63306 | 2.91E-09 | 5.12E-07 | 5.5878 | 5.5712 | 5.5561 | 3.4077 | 3.4071 | 3.4205 |
| 3,4,5-trihydroxy-6-{[1-hydroxy-1-(1-oxo-1H-isochromen-3-yl)but-3-en-2-yl]oxy}oxane-2-carboxylic acid |  | 2.470025 | 2.469766 | 3.488682 | 1.78E-09 | 4.00E-07 | 5.2418 | 5.2654 | 5.2137 | 1.498 | 1.4974 | 1.5104 |
| 6-(4-ethenyl-5-hydroxy-2-methoxyphenoxy)-3,4,5-trihydroxyoxane-2-carboxylic acid |  | 2.127417 | 2.128173 | 1.881714 | 0.005452 | 0.007834 | 5.8919 | 5.8762 | 5.8884 | 2.162 | 3.8645 | 3.3565 |
| 6-{[3-(6,7-dimethoxy-2H-1,3-benzodioxol-5-yl)oxiran-2-yl]methoxy}-3,4,5-trihydroxyoxane-2-carboxylic acid |  | 1.87381 | 1.873739 | 1.552834 | 7.58E-09 | 8.17E-07 | 5.628 | 5.6566 | 5.643 | 3.6171 | 3.6439 | 3.6421 |
| 3,4,5-trihydroxy-6-(3-oxo-1,3-diphenylpropoxy)oxane-2-carboxylic acid |  | 2.203444 | 2.203445 | 2.362707 | 9.51E-05 | 0.000287 | 4.854 | 4.8663 | 4.8384 | 1.8639 | 2.4086 | 1.8886 |
| 3,4,5-trihydroxy-6-[3-(3-oxo-3-phenylpropyl)phenoxy]oxane-2-carboxylic acid |  | 1.782192 | 1.782035 | 1.532333 | 0.000331 | 0.000743 | 5.3027 | 5.3075 | 5.3139 | 3.6924 | 3.1538 | 3.5468 |
| 6-({8-[(3,3-dimethyloxiran-2-yl)methyl]-2-oxo-2H-chromen-7-yl}oxy)-3,4,5-trihydroxyoxane-2-carboxylic acid |  | 1.969045 | 1.969046 | 1.69226 | 1.90E-08 | 1.29E-06 | 5.4479 | 5.3992 | 5.4175 | 3.1865 | 3.2133 | 3.2115 |
| Tyrosyl-Hydroxyproline | Amino acids and their derivatives | 2.277726 | 2.277433 | 3.06697 | 1.00E-07 | 3.67E-06 | 4.7235 | 4.6594 | 4.7688 | 1.5206 | 1.5201 | 1.5724 |
| L-Cys-Gly |  | 2.708568 | 2.708155 | 0.027141 | 1.16E-06 | 1.54E-05 | 0.0011 | 0.3063 | 0.0691 | 4.6596 | 4.6166 | 4.5964 |
| Tryptophyl-Threonine |  | 1.73176 | 1.731674 | 1.532423 | 4.83E-08 | 2.23E-06 | 4.9157 | 4.9633 | 4.9372 | 3.2061 | 3.2328 | 3.2311 |
| Phenylalanyl-Asparagine |  | 2.33136 | 2.331422 | 2.221263 | 2.49E-06 | 2.29E-05 | 5.6851 | 5.6615 | 5.639 | 2.3936 | 2.6164 | 2.6371 |
| Asparaginyl-Lysine |  | 2.484945 | 2.485291 | 2.409945 | 0.000539 | 0.001102 | 6.1407 | 6.1542 | 6.1715 | 1.8763 | 3.0824 | 2.7047 |
| Tyrosyl-Isoleucine |  | 1.816842 | 1.817068 | 1.60315 | 0.00028 | 0.000652 | 5.0793 | 5.1199 | 5.0701 | 2.8695 | 3.2499 | 3.4062 |
| Histidinyl-Isoleucine |  | 1.821053 | 1.821033 | 1.637005 | 8.66E-09 | 8.38E-07 | 4.8835 | 4.8588 | 4.8812 | 2.9604 | 2.9872 | 2.9855 |
| Tyr Glu Arg Arg |  | 2.238807 | 2.238721 | 2.176426 | 8.35E-05 | 0.000277 | 5.7102 | 5.7457 | 5.7167 | 2.44 | 2.4394 | 3.0094 |
| Ile Ala Ala Phe |  | 2.085899 | 2.085875 | 1.867852 | 7.04E-08 | 2.76E-06 | 5.3672 | 5.3392 | 5.3631 | 2.8317 | 2.9141 | 2.8567 |
| Gly Leu Phe |  | 1.834521 | 1.835027 | 1.672414 | 0.003768 | 0.005638 | 5.0253 | 5.0424 | 5.0643 | 2.391 | 3.538 | 3.1187 |
| Ile Ile Leu |  | 2.370058 | 2.369997 | 2.756417 | 1.61E-06 | 1.70E-05 | 5.0266 | 5.0347 | 5.0784 | 1.7473 | 1.9729 | 1.7719 |
| Leu Ala Phe |  | 1.860465 | 1.860648 | 1.638407 | 2.29E-05 | 0.000103 | 5.1669 | 5.0745 | 5.0635 | 2.9506 | 3.2074 | 3.1833 |
| Leu Ala Ile |  | 1.740234 | 1.740167 | 1.572876 | 0.000214 | 0.000527 | 4.8216 | 4.7833 | 4.8342 | 3.2058 | 2.7883 | 3.1863 |
| Ile Leu Tyr |  | 1.883914 | 1.883621 | 1.677556 | 0.000701 | 0.00136 | 5.1607 | 5.1296 | 5.1151 | 3.4573 | 3.0254 | 2.7003 |
| His Ile |  | 2.479886 | 2.480217 | 4.851085 | 0.01421 | 0.01855 | 4.9506 | 4.8954 | 4.9102 | 0.0727 | 2.8937 | 0.0767 |
| Val His |  | 2.066884 | 2.067328 | 1.738095 | 0.001062 | 0.001912 | 5.9045 | 5.9194 | 5.9156 | 2.8097 | 3.6834 | 3.7125 |
| Gly-His-OH |  | 2.233997 | 2.233923 | 2.296549 | 1.18E-08 | 1.00E-06 | 5.0444 | 5.0838 | 5.0428 | 2.1781 | 2.2047 | 2.2247 |
| His Pro Gly |  | 1.911256 | 1.911204 | 1.631531 | 4.88E-07 | 8.08E-06 | 5.4139 | 5.3589 | 5.432 | 3.3619 | 3.2667 | 3.3052 |
| 2-Methoxy-1,4-benzoquinone | Carbohydrates and other organic oxygen compounds | 2.058498 | 2.058263 | 2.058272 | 6.15E-09 | 6.90E-07 | 5.0361 | 5.056 | 5.0601 | 2.4358 | 2.4785 | 2.4485 |
| Isobarbaloin |  | 2.399898 | 2.399796 | 0.404017 | 4.43E-09 | 6.92E-07 | 2.2419 | 2.2256 | 2.2314 | 5.4949 | 5.5497 | 5.5364 |
| Chlorogenoquinone |  | 2.680504 | 2.680197 | 4.303076 | 1.77E-11 | 1.08E-07 | 5.7338 | 5.7299 | 5.7433 | 1.3293 | 1.3288 | 1.3415 |
| Galactose-beta-1,4-xylose |  | 1.886068 | 1.886143 | 1.802435 | 2.52E-05 | 0.00011 | 4.5914 | 4.5791 | 4.5967 | 2.4403 | 2.467 | 2.7307 |
| B-D-Glucopyranosiduronic acid |  | 2.009088 | 2.009042 | 1.744358 | 1.37E-09 | 3.50E-07 | 5.4131 | 5.4134 | 5.4061 | 3.085 | 3.1118 | 3.11 |
| B-D-fructosyl-a-D-(6-O-(E))-feruloylglucoside |  | 1.666773 | 1.666508 | 1.517776 | 1.78E-06 | 2.14E-05 | 5.0123 | 4.9199 | 5.0537 | 3.2873 | 3.2867 | 3.3001 |
| 1-O-2'-Hydroxy-4'-methoxycinnamoyl-b-D-glucose |  | 1.922878 | 1.922796 | 1.612334 | 4.05E-08 | 1.95E-06 | 5.5539 | 5.6036 | 5.5507 | 3.4372 | 3.464 | 3.4622 |
| 2-Acetyl-3-ethylpyrazine |  | 1.584092 | 1.584051 | 1.613248 | 4.01E-08 | 1.94E-06 | 3.7748 | 3.7921 | 3.7585 | 2.3225 | 2.3492 | 2.3475 |
| (S)-Rutaretin | Coumarins and derivatives | 2.74015 | 2.741085 | 5.547101 | 0.01017 | 0.01415 | 6.149 | 6.0621 | 6.1599 | 3.2945 | 0.0091 | 0.0094 |
| 8-hydroxy-6-methyl-2H-chromen-2-one |  | 1.791182 | 1.790984 | 1.74611 | 4.28E-10 | 1.92E-07 | 4.6072 | 4.602 | 4.5944 | 2.6313 | 2.6307 | 2.6441 |
| Citalopram propionic acid |  | 1.758862 | 1.758707 | 1.54462 | 9.30E-07 | 1.37E-05 | 5.3087 | 5.4274 | 5.4115 | 3.4799 | 3.4794 | 3.497 |
| 3-(6,7-dimethoxy-2H-1,3-benzodioxol-5-yl)propanal | Others | 1.839269 | 1.839077 | 1.604179 | 2.88E-05 | 0.000124 | 5.5353 | 5.4684 | 5.5794 | 3.5393 | 3.2611 | 3.5369 |
| 3-(6-hydroxy-7-methoxy-2H-1,3-benzodioxol-5-yl)prop-2-enal |  | 1.936152 | 1.936102 | 1.826133 | 0.000821 | 0.001637 | 5.197 | 5.1324 | 5.2663 | 3.3597 | 2.5834 | 2.5968 |
| 1-(4-methoxy-1-benzofuran-5-yl)ethan-1-one |  | 1.50251 | 1.502493 | 1.502146 | 0.000327 | 0.000787 | 4.2026 | 4.2009 | 4.198 | 2.6743 | 2.6738 | 3.0406 |
| 9'-Carboxy-gamma-chromanol |  | 2.409635 | 2.40969 | 0.399533 | 0.000232 | 0.0006 | 2.1137 | 2.1095 | 2.9722 | 5.9993 | 6.0139 | 5.9916 |
| 3,5,6,7-tetrahydroxy-2-(3,4,5-trihydroxyphenyl)-3,4-dihydro-2H-1-benzopyran-4-one |  | 1.682375 | 1.682232 | 1.58124 | 3.74E-07 | 7.82E-06 | 4.6718 | 4.7655 | 4.7225 | 2.9806 | 2.9801 | 2.9934 |
| 2-(3,4-dihydroxyphenyl)-3,7-dihydroxy-3,4-dihydro-2H-1-benzopyran-5-yl 3,4,5-trihydroxybenzoate |  | 2.799991 | 2.799624 | 198.2348 | 2.82E-08 | 1.72E-06 | 4.8349 | 4.7579 | 4.8927 | 0.0165 | 0.0396 | 0.017 |
| 4-{hydroxy[(3,4,5,6-tetrahydroxyoxan-2-yl)methoxy]methylidene}cyclohexa-2,5-dien-1-one |  | 1.966631 | 1.96642 | 1.685014 | 1.44E-05 | 8.02E-05 | 5.8829 | 5.7768 | 5.8826 | 3.4045 | 3.3639 | 3.6425 |
| Tetrahydropentoxyline |  | 2.029316 | 2.02935 | 1.585693 | 1.38E-06 | 1.55E-05 | 6.3968 | 6.3863 | 6.3683 | 3.9246 | 4.0683 | 4.0854 |
| 5,7-dihydroxy-2-(3-hydroxyphenyl)-3,6-dimethoxy-3,4-dihydro-2H-1-benzopyran-4-one |  | 2.000338 | 2.003287 | 0.48851 | 0.04032 | 0.04867 | 0.7907 | 3.581 | 3.5366 | 5.3904 | 5.4141 | 5.3832 |
| 6-Hydroxynicotine (S) |  | 2.095846 | 2.095824 | 2.014534 | 5.21E-09 | 7.07E-07 | 5.0098 | 4.9852 | 4.974 | 2.46 | 2.4867 | 2.485 |
| 5-formyl-6-hydroxy-1,1,4a,6-tetramethyl-decahydronaphthalen-2-yl acetate |  | 1.794306 | 1.794136 | 1.550089 | 8.40E-06 | 5.11E-05 | 5.1897 | 5.2132 | 5.1947 | 3.4802 | 3.2915 | 3.2897 |
| 5,7-dihydroxy-6-(3-methylbut-2-en-1-yl)-2-(2,4,5-trihydroxyphenyl)-3,4-dihydro-2H-1-benzopyran-4-one |  | 1.938441 | 1.938236 | 1.608915 | 0.000175 | 0.000455 | 5.7558 | 5.7228 | 5.7375 | 3.7308 | 3.7241 | 3.2447 |
| N,N,O-Didesmethylvenlafaxine |  | 2.376144 | 2.375824 | 0.372233 | 2.97E-06 | 2.55E-05 | 2.0784 | 1.834 | 1.8397 | 5.0932 | 5.193 | 5.1646 |
| 1-(3,5-dihydroxyphenyl)-3-[4-hydroxy-2-methoxy-3-(3-methylbut-2-en-1-yl)phenyl]prop-2-en-1-one |  | 1.953837 | 1.953777 | 1.620455 | 8.98E-08 | 3.16E-06 | 5.6808 | 5.6923 | 5.7381 | 3.4918 | 3.5186 | 3.5495 |
| 4-Hydroxymethylphenylhydrazine |  | 1.599804 | 1.599932 | 1.507381 | 0.000137 | 0.000378 | 4.3711 | 4.4168 | 4.3844 | 2.7103 | 2.9902 | 3.0374 |

**Table S3**. List of Volatile Metabolites Determined by GC-IMS before and after Processing of Jujube Leaves

| No. | compound | organics | Formulas | RI | Rt [sec] | Dt [a.u.] | [+] D1 | [+] D1 | [+] D1 | [+] D2 | [+] D2 | [+] D2 |
| --- | --- | --- | --- | --- | --- | --- | --- | --- | --- | --- | --- | --- |
| 1 | Ethyl propanotate | esters | C5H10O2 | 934.8 | 236.003 | 1.45625 | 523.3359 | 478.3886 | 447.591 | 19.56467 | 22.82756 | 15.8352 |
| 2 | Ethyl acetate |  | C4H8O2 | 862.9 | 220.616 | 1.33663 | 4619.046 | 4743.966 | 4678.035 | 290.5468 | 233.8524 | 300.3831 |
| 3 | Isoamyl acetate |  | C7H14O2 | 1123.1 | 308.469 | 1.75283 | 173.5428 | 250.6747 | 222.0673 | 20.50736 | 19.7932 | 21.61509 |
| 4 | (E)-Ethyl-2-hexenoate |  | C8H14O2 | 1350.6 | 535.479 | 1.81695 | 1085.833 | 891.4335 | 963.6489 | 58.49722 | 61.54111 | 69.57139 |
| 5 | Ethyl isobutyrate |  | C6H12O2 | 953.8 | 240.237 | 1.56474 | 1667.761 | 1844.694 | 1666.923 | 54.95819 | 60.1128 | 50.68913 |
| 6 | Ethyl crotonate |  | C6H10O2 | 1163.4 | 337.877 | 1.56063 | 6742.657 | 7140.083 | 7090.844 | 154.7939 | 151.2104 | 164.3572 |
| 7 | Ethyl butanoate |  | C6H12O2 | 1028.2 | 262.285 | 1.56178 | 3879.816 | 3214.7 | 3644.45 | 38.76433 | 40.49417 | 28.91851 |
| 8 | Ethyl pentanoate |  | C7H14O2 | 1131.7 | 314.536 | 1.67773 | 171.5368 | 187.0546 | 147.5317 | 24.31301 | 23.21162 | 20.21852 |
| 9 | Ethyl 3-methylbutanoate |  | C7H14O2 | 1047.4 | 269.916 | 1.65894 | 7453.616 | 7723.09 | 7546.351 | 155.6255 | 142.4057 | 158.9074 |
| 10 | Ethyl hexanoate |  | C8H16O2 | 1232.3 | 396.228 | 1.80309 | 3331.295 | 3118.143 | 3168.68 | 118.5434 | 121.0382 | 111.4558 |
| 11 | Butyl propionate |  | C7H14O2 | 1135.3 | 317.079 | 1.72635 | 76.38284 | 85.41293 | 77.68736 | 16.98419 | 13.22932 | 14.70207 |
| 12 | Propyl butanoate |  | C7H14O2 | 1121.1 | 307.065 | 1.69317 | 96.35696 | 114.5568 | 104.9744 | 16.89532 | 23.45602 | 13.73082 |
| 13 | Hexyl acetate |  | C8H16O2 | 1273.9 | 436.952 | 1.8963 | 560.7862 | 460.0713 | 394.7182 | 35.74266 | 35.21895 | 30.22938 |
| 14 | Methyl acetate |  | C3H6O2 | 826.9 | 213.301 | 1.1956 | 290.4452 | 284.7256 | 258.0861 | 41.60508 | 44.18556 | 67.77171 |
| 15 | (Z)-3-Hexenyl butyrate(dipolymer) |  | C10H18O2 | 1451.3 | 707.574 | 2.00795 | 1644.892 | 1343.087 | 1486.334 | 59.82714 | 60.08741 | 58.87811 |
| 16 | (Z)-3-Hexenyl butyrate(monomer) |  | C10H18O2 | 1451.9 | 708.763 | 1.42074 | 603.7498 | 524.577 | 549.8549 | 46.3312 | 48.48319 | 50.54313 |
| 17 | (Z)-3-Hexenyl acetate |  | C8H14O2 | 1322.7 | 495.69 | 1.81961 | 4620.871 | 5099.266 | 4745.823 | 70.72356 | 93.72569 | 92.6497 |
| 18 | Hexyl 2-methylbutanoate(monomer) |  | C11H22O2 | 1429.9 | 666.843 | 1.51572 | 506.1613 | 406.7445 | 640.2986 | 124.647 | 138.9333 | 127.9099 |
| 19 | Hexyl 2-methylbutanoate(dipolymer) |  | C11H22O2 | 1429.9 | 666.843 | 2.15274 | 104.876 | 64.804 | 61.95056 | 51.45724 | 47.19136 | 45.73448 |
| 20 | Linalool | alcohols | C10H18O | 1513 | 839.143 | 1.22365 | 3449.533 | 3103.797 | 3574.091 | 186.277 | 151.9023 | 177.3167 |
| 21 | 3-Methyl-2-butanol |  | C5H12O | 1104 | 295.435 | 1.42756 | 1942.511 | 1665.095 | 1622.89 | 232.1257 | 230.5609 | 175.7869 |
| 22 | Ethanol |  | C2H6O | 908.7 | 230.302 | 1.14683 | 3673.787 | 3654.324 | 3728.381 | 1686.796 | 1586.84 | 1528.028 |
| 23 | 1-Hexanol( trimer) |  | C6H14O | 1357.2 | 545.285 | 1.70574 | 922.7675 | 836.3706 | 974.2438 | 68.13355 | 55.34859 | 40.52274 |
| 24 | 1-Hexanol(dipolymer) |  | C6H14O | 1358.5 | 547.266 | 1.64319 | 1273.697 | 1153.881 | 1417.071 | 325.6356 | 134.8896 | 84.18458 |
| 25 | 1-Hexanol(monomer) |  | C6H14O | 1357.4 | 545.681 | 1.32741 | 313.1839 | 296.3521 | 265.1863 | 857.6747 | 504.9044 | 366.9423 |
| 26 | (Z)-Hex-3-enol(trimer) |  | C6H12O | 1391.2 | 599.19 | 1.71414 | 652.6519 | 570.2575 | 650.6682 | 90.12318 | 97.32186 | 105.1776 |
| 27 | (Z)-Hex-3-enol(dipolymer) |  | C6H12O | 1391.6 | 599.763 | 1.50818 | 5608.198 | 5646.359 | 5991.233 | 558.6882 | 574.2123 | 564.3887 |
| 28 | (Z)-Hex-3-enol(monomer) |  | C6H12O | 1390.8 | 598.462 | 1.23309 | 293.8256 | 252.5284 | 258.4543 | 431.2448 | 441.3287 | 428.7977 |
| 29 | 3-Methyl-1-butanol(dipolymer) |  | C5H12O | 1199.9 | 367.262 | 1.50882 | 2062.473 | 2093.953 | 1953.214 | 903.298 | 455.7991 | 463.0771 |
| 30 | 3-Methyl-1-butanol(monomer) |  | C5H12O | 1200.9 | 368.122 | 1.25183 | 85.75572 | 84.62894 | 84.47342 | 516.1721 | 357.7757 | 294.6921 |
| 31 | 2-Methyl-1-propanol |  | C4H10O | 1082.7 | 284.603 | 1.17271 | 39.70384 | 45.61704 | 40.60209 | 200.0713 | 159.3073 | 136.2639 |
| 32 | 1-Penten-3-ol |  | C5H10O | 1157.9 | 333.739 | 0.94496 | 175.7266 | 152.5594 | 149.7757 | 1408.869 | 1386.546 | 1451.077 |
| 33 | (Z)-2-Pentenol |  | C5H10O | 1325.8 | 499.988 | 0.94115 | 143.3325 | 108.7198 | 126.0309 | 898.8957 | 970.0668 | 995.2748 |
| 34 | Butan-1-ol |  | C4H10O | 1136.6 | 318.008 | 1.17898 | 52.30788 | 35.79662 | 24.51297 | 150.1979 | 154.9684 | 132.7598 |
| 35 | 1-Pentanol(dipolymer) |  | C5H12O | 1246.8 | 409.958 | 1.51682 | 103.9968 | 74.33559 | 99.71825 | 992.3357 | 956.628 | 1215.479 |
| 36 | 1-Pentanol(monomer) |  | C5H12O | 1245.5 | 408.688 | 1.25512 | 551.1911 | 563.7603 | 595.0244 | 716.5354 | 763.9807 | 736.6016 |
| 37 | Pentanal | aldehydes | C5H10O | 978.8 | 245.932 | 1.42567 | 80.35037 | 67.61619 | 67.97485 | 820.6148 | 818.0946 | 811.6958 |
| 38 | Nonanal(dipolymer) |  | C9H18O | 1400.9 | 615.425 | 1.95236 | 144.8528 | 125.7611 | 111.7922 | 589.965 | 632.8809 | 665.5543 |
| 39 | Nonanal(monomer) |  | C9H18O | 1402.3 | 617.803 | 1.47633 | 320.3255 | 310.7716 | 332.1773 | 1696.616 | 1731.911 | 1776.608 |
| 40 | Furfural(monomer) |  | C5H4O2 | 1454.3 | 713.407 | 1.08586 | 61.02692 | 53.05695 | 53.06965 | 203.5564 | 256.0325 | 271.5281 |
| 41 | Furfural(dipolymer) |  | C5H4O2 | 1455.8 | 716.443 | 1.33815 | 33.99377 | 28.99786 | 37.22175 | 39.09443 | 34.90154 | 34.91424 |
| 42 | (E)-2-Pentenal(dipolymer) |  | C5H8O | 1137.4 | 318.577 | 1.36234 | 73.13581 | 55.739 | 61.84899 | 412.8736 | 321.3666 | 563.5063 |
| 43 | (E)-2-Pentenal(monomer) |  | C5H8O | 1137.9 | 318.972 | 1.10657 | 42.81438 | 41.10993 | 46.96917 | 167.8518 | 158.3012 | 187.4482 |
| 44 | Heptanal(dipolymer) |  | C7H14O | 1185.8 | 355.503 | 1.6939 | 146.0272 | 122.6093 | 132.9153 | 444.2202 | 445.1185 | 464.3277 |
| 45 | Heptanal(monomer) |  | C7H14O | 1187.6 | 356.937 | 1.33571 | 208.2857 | 192.3648 | 221.5213 | 670.3407 | 691.1718 | 696.6566 |
| 46 | Propionaldehyde |  | C3H6O | 789.2 | 205.892 | 1.13917 | 223.34 | 208.8157 | 229.8912 | 567.8706 | 549.4327 | 582.9504 |
| 47 | (E)-2-Heptenal |  | C7H12O | 1332.2 | 508.842 | 1.25313 | 72.61527 | 67.51462 | 51.46042 | 86.44131 | 114.1632 | 118.0482 |
| 48 | (E)-2-Hexenal(dipolymer) |  | C6H10O | 1224 | 388.624 | 1.51911 | 1550.738 | 1265.854 | 1492.616 | 4587.55 | 4343.662 | 5334.15 |
| 49 | (E)-2-Hexenal(monomer) |  | C6H10O | 1224 | 388.624 | 1.17937 | 232.4146 | 235.7346 | 228.3233 | 1868.658 | 1985.532 | 1926.879 |
| 50 | Benzaldehyde(monomer) |  | C7H6O | 1486 | 778.923 | 1.15023 | 412.7625 | 395.5688 | 416.3555 | 350.6786 | 359.696 | 437.8246 |
| 51 | Benzaldehyde(dipolymer) |  | C7H6O | 1486.5 | 779.913 | 1.47353 | 74.11341 | 72.27882 | 71.13935 | 42.07483 | 44.24269 | 51.0954 |
| 52 | Hexanal(dipolymer) |  | C6H12O | 1089.2 | 287.377 | 1.56476 | 63.45187 | 48.76567 | 58.256 | 366.4853 | 365.3109 | 431.3369 |
| 53 | Hexanal(monomer) |  | C6H12O | 1087.7 | 286.764 | 1.2622 | 375.5598 | 369.5196 | 386.8657 | 476.992 | 507.142 | 524.8309 |
| 54 | Butane-2,3-dione | ketones | C4H6O2 | 961.1 | 241.88 | 1.17639 | 43.92846 | 45.94079 | 53.48227 | 266.1576 | 299.8562 | 348.9075 |
| 55 | Butan-2-one |  | C4H8O | 885.9 | 225.429 | 1.24678 | 83.23238 | 75.47824 | 71.87573 | 533.369 | 513.8107 | 557.9581 |
| 56 | 1-Penten-3-one(dipolymer) |  | C5H8O | 1486.5 | 779.913 | 1.47353 | 74.11341 | 72.27882 | 71.13935 | 42.07483 | 44.24269 | 51.0954 |
| 57 | 1-Penten-3-one(monomer) |  | C5H8O | 1486 | 778.923 | 1.15023 | 412.7625 | 395.5688 | 416.3555 | 350.6786 | 359.696 | 437.8246 |
| 58 | 1-Hydroxy-2-propanone(dipolymer) |  | C3H6O2 | 1309.7 | 478.11 | 1.23156 | 107.2026 | 81.18195 | 89.91052 | 172.3367 | 305.3028 | 347.0698 |
| 59 | 1-Hydroxy-2-propanone(monomer) |  | C3H6O2 | 1312 | 481.218 | 1.06253 | 135.6355 | 131.2617 | 133.6104 | 667.7095 | 867.1491 | 891.0971 |
| 60 | 3-Hydroxy-2-butanone(dipolymer) |  | C4H8O2 | 1296.1 | 460.476 | 1.06475 | 423.6081 | 575.7327 | 296.7234 | 2011.689 | 1618.561 | 1358.193 |
| 61 | 3-Hydroxy-2-butanone(monomer) |  | C4H8O2 | 1296.1 | 460.476 | 1.06475 | 213.4752 | 222.4894 | 157.0823 | 1190.499 | 1163.387 | 1103.639 |
| 62 | 4-methyl-3-penten-2-one(dipolymer) |  | C6H10O | 1138.3 | 319.28 | 1.45229 | 44.50613 | 35.92358 | 38.00256 | 223.5273 | 262.2155 | 189.4828 |
| 63 | 4-methyl-3-penten-2-one(monomer) |  | C6H10O | 1140.3 | 320.677 | 1.11813 | 313.4569 | 351.3738 | 326.0831 | 310.4479 | 346.3461 | 291.9751 |
| 64 | 6-Methylhept-5-en-2-one |  | C8H14O | 1342.7 | 523.891 | 1.17698 | 506.393 | 405.4369 | 381.8666 | 1196.594 | 1204.878 | 1385.321 |
| 65 | 2-Pentanone |  | C5H10O | 974.4 | 244.91 | 1.37339 | 322.6457 | 211.6724 | 294.7809 | 557.3201 | 499.2831 | 474.4591 |
| 66 | Acetone |  | C3H6O | 828.2 | 213.563 | 1.12009 | 2208.304 | 2166.435 | 2339.181 | 4534.779 | 4521.794 | 4353.444 |
| 67 | 2-Methyl-dihydro-(2 H)-furan-3-one |  | C5H8O2 | 1271.2 | 434.163 | 1.07132 | 46.05188 | 38.41836 | 35.30465 | 64.83257 | 83.91161 | 58.99237 |
| 68 | beta-Pinene | terpenes | C10H16 | 1124.9 | 309.742 | 1.21992 | 238.9118 | 217.0714 | 245.1361 | 28.4678 | 32.39724 | 27.78856 |
| 69 | beta-Ocimene(polymer) |  | C10H16 | 1250.4 | 413.505 | 1.69371 | 2426.394 | 2465.971 | 2364.58 | 230.088 | 206.54 | 236.1948 |
| 70 | beta-Ocimene(monomer) |  | C10H16 | 1254.3 | 417.216 | 1.21283 | 1039.343 | 1019.296 | 1030.148 | 103.8953 | 87.63157 | 95.1667 |
| 71 | Acetic acid(monomer) | acids | C2H4O2 | 1469.3 | 743.602 | 1.05895 | 1487.413 | 1642.061 | 1502.138 | 3479.925 | 3511.493 | 3549.934 |
| 72 | Acetic acid(dipolymer) |  | C2H4O2 | 1468.1 | 741.137 | 1.16135 | 459.4365 | 741.5753 | 481.1373 | 1167.85 | 1159.635 | 1213.362 |
| 73 | Dimethyl sulfide(monomer) | sulfoethers | C2H6S | 777 | 203.548 | 0.96399 | 309.0989 | 308.753 | 312.2031 | 525.3546 | 584.877 | 479.4043 |
| 74 | Dimethyl sulfide(dipolymer) |  | C2H6S | 776.5 | 203.45 | 1.0831 | 1474.549 | 1444.075 | 1410.726 | 1009.45 | 1010.558 | 916.6798 |
| 75 | 1 | other classes | unidentified | 1659.2 | 1257.532 | 1.49356 | 2619.46 | 2480.701 | 2081.508 | 192.6092 | 219.947 | 226.1046 |
| 76 | 2 |  | unidentified | 1581.2 | 1013.553 | 1.27331 | 239.5307 | 253.6043 | 227.6091 | 116.585 | 105.5521 | 115.5439 |
| 77 | 3 |  | unidentified | 1544.4 | 915.37 | 1.36802 | 1228.699 | 1254.126 | 1630.152 | 131.2236 | 164.1446 | 148.2617 |
| 78 | 4 |  | unidentified | 1458.5 | 721.842 | 1.45027 | 1307.507 | 1410.884 | 1275.576 | 160.4563 | 160.7864 | 188.3242 |
| 79 | 5 |  | unidentified | 1458.8 | 722.437 | 2.08961 | 2747.233 | 2970.729 | 2979.039 | 163.6716 | 160.1738 | 195.415 |
| 80 | 6 |  | unidentified | 1427.2 | 661.797 | 1.45375 | 117.1405 | 90.09144 | 101.7528 | 970.2096 | 928.6934 | 841.5919 |
| 81 | 7 |  | unidentified | 1369.4 | 563.989 | 1.32053 | 443.0808 | 438.5102 | 464.0103 | 274.299 | 193.9867 | 123.6885 |
| 82 | 8 |  | unidentified | 1331.1 | 507.308 | 1.75138 | 106.9233 | 118.9401 | 100.3467 | 26.03333 | 28.48367 | 28.53763 |
| 83 | 9 |  | unidentified | 1306.2 | 473.578 | 1.74841 | 353.8781 | 345.6573 | 339.5854 | 45.3663 | 23.66233 | 25.06208 |
| 84 | 10 |  | unidentified | 1286.5 | 450.011 | 1.09264 | 26.7824 | 28.14723 | 26.81731 | 141.7328 | 172.2954 | 182.8681 |
| 85 | 11 |  | unidentified | 1262.2 | 425.12 | 1.68358 | 269.5252 | 284.3416 | 237.0423 | 34.05408 | 31.78466 | 39.78001 |
| 86 | 12 |  | unidentified | 1223.2 | 387.911 | 1.60336 | 107.4311 | 115.8835 | 94.33511 | 26.7824 | 22.69108 | 17.48569 |
| 87 | 13 |  | unidentified | 1237.8 | 401.39 | 1.72586 | 1293.103 | 1377.897 | 1257.859 | 45.00446 | 41.91613 | 43.5698 |
| 88 | 14 |  | unidentified | 1205.5 | 372.137 | 1.18125 | 63.86449 | 66.35928 | 60.50955 | 274.3212 | 297.682 | 421.0848 |
| 89 | 15 |  | unidentified | 1208.2 | 374.431 | 1.66594 | 390.6618 | 368.5642 | 468.5047 | 28.64237 | 27.01093 | 35.55539 |
| 90 | 16 |  | unidentified | 1153.6 | 330.468 | 1.33874 | 91.96729 | 80.53763 | 92.35769 | 236.0679 | 213.3704 | 223.6575 |
| 91 | 17 |  | unidentified | 1132.2 | 314.89 | 1.07743 | 35.92993 | 39.72923 | 42.63346 | 142.6564 | 143.5927 | 105.5077 |
| 92 | 18 |  | unidentified | 1127.8 | 311.774 | 1.11682 | 117.8546 | 142.1168 | 113.3602 | 184.6011 | 242.4953 | 326.2799 |
| 93 | 19 |  | unidentified | 1114.1 | 302.227 | 1.41638 | 646.3292 | 566.6423 | 574.6218 | 25.33822 | 24.05909 | 22.31972 |
| 94 | 20 |  | unidentified | 1107.9 | 298.005 | 1.09558 | 25.76988 | 30.62614 | 32.14332 | 105.3363 | 107.8311 | 105.1871 |
| 95 | 21 |  | unidentified | 1099.1 | 292.13 | 1.10229 | 44.96637 | 41.43686 | 34.24452 | 109.5545 | 122.9775 | 104.7586 |
| 96 | 22 |  | unidentified | 1101 | 293.415 | 1.53598 | 543.656 | 565.8519 | 574.8471 | 51.74291 | 52.79351 | 39.17061 |
| 97 | 23 |  | unidentified | 1107.6 | 297.821 | 1.5997 | 140.1521 | 134.7118 | 138.4699 | 12.56913 | 10.16004 | 11.94384 |
| 98 | 24 |  | unidentified | 1101.3 | 293.599 | 1.76177 | 136.3877 | 181.4239 | 268.7444 | 13.64512 | 11.70897 | 18.05701 |
| 99 | 25 |  | unidentified | 1093 | 289.009 | 1.70141 | 100.0515 | 95.04291 | 89.29158 | 16.46365 | 17.68248 | 17.94592 |
| 100 | 26 |  | unidentified | 1009.4 | 254.984 | 1.11103 | 255.3278 | 286.7538 | 270.0426 | 567.0707 | 593.6818 | 618.0201 |
| 101 | 27 |  | unidentified | 1012 | 256.006 | 1.33493 | 389.827 | 396.8321 | 410.0804 | 29.75328 | 26.70939 | 30.48966 |
| 102 | 28 |  | unidentified | 975 | 245.056 | 1.35761 | 1154.681 | 755.4299 | 877.5313 | 496.1758 | 454.9706 | 437.085 |
| 103 | 29 |  | unidentified | 1070.4 | 279.398 | 1.12809 | 168.7532 | 203.2771 | 169.1151 | 218.6806 | 217.3189 | 219.0615 |
| 104 | 30 |  | unidentified | 1394.8 | 605.174 | 1.99749 | 192.241 | 175.7805 | 144.1196 | 28.55033 | 26.0365 | 30.14686 |
| 105 | 31 |  | unidentified | 1414.9 | 639.785 | 1.09707 | 73.15485 | 75.11958 | 74.66569 | 135.7974 | 167.49 | 171.1909 |
| 106 | 32 |  | unidentified | 1382.6 | 584.997 | 1.0902 | 223.0956 | 246.9421 | 266.6972 | 112.2112 | 119.2861 | 126.0849 |
| 107 | 33 |  | unidentified | 1351.2 | 536.395 | 1.10309 | 129.2589 | 122.0761 | 155.5334 | 115.6709 | 171.7305 | 176.5931 |
| 108 | 34 |  | unidentified | 1330.8 | 506.959 | 1.42282 | 55.5676 | 54.39321 | 40.78936 | 172.6478 | 173.9745 | 203.404 |
| 109 | 35 |  | unidentified | 1310.4 | 479.146 | 1.26406 | 1746.375 | 1693.693 | 1762.03 | 678.7709 | 539.0346 | 473.9481 |
| 110 | 36 |  | unidentified | 1310.2 | 478.801 | 1.31499 | 677.3521 | 676.9363 | 633.0904 | 383.6948 | 284.859 | 237.4549 |
| 111 | 37 |  | unidentified | 1310.7 | 479.436 | 1.79092 | 361.6544 | 322.4425 | 333.2755 | 39.38326 | 26.36025 | 26.73479 |
| 112 | 38 |  | unidentified | 1310.4 | 479.131 | 1.83465 | 793.7118 | 727.6445 | 676.3682 | 82.15003 | 65.56259 | 56.75786 |

**Table S4.** List of Volatile Metabolites Determined by GC-MS before and after Processing of Jujube Leaves

| RT (min) | Compounds | D1（mg/L） | D2（mg/L) | odor threshold（µg/L) | OAV(D1) | OAV(D2) |
| --- | --- | --- | --- | --- | --- | --- |
| 8.493 | Allyl ethyl ether | —— | 0.1832551 | —— | —— | —— |
| 8.946 | 2-ethyl-Furan | 6.512972771 | —— | 2.3 | 2831.727 | —— |
| 11.728 | Hexanal | 5.035562854 | 7.4222 | 4500 | 1.119014 | 1.649378 |
| 13.24 | 4-methyl-3-Penten-2-one | 0.94120334 | 0.9582967 | —— | —— | —— |
| 13.817 | 2-Ethylthiophene | 0.362618915 | —— | —— | —— | —— |
| 13.981 | 1-Penten-3-ol | 2.267313089 | 4.9823882 | 400 | 5.668283 | 12.45597 |
| 14.552 | D-Limonene | —— | 0.2573489 | 34 |  | 7.569084 |
| 15.552 | 2-pentyl-Furan | 1.853057496 | 1.0420425 | 6 | 308.8429 | 173.6737 |
| 15.84 | (E)-2-Hexenal | 4.586262855 | 7.3357213 | 17 | 269.7802 | 431.513 |
| 16.552 | 1-Pentanol | 0.456477327 | 0.3829232 | 1.93 | 236.5167 | 198.4058 |
| 16.81 | 2-ethenyl-1,1-dimethyl-3-methylene-Cyclohexane | 0.965113635 | —— | —— | —— | —— |
| 17.022 | Benzene, 4-ethyl-1,2-dimethyl- | —— | 0.147779 | —— | —— | —— |
| 17.14 | trans-2-(2-Pentenyl)furan | 0.970602633 | —— | —— | —— | —— |
| 18.128 | (Z)-3-Hexenyl acetate | 6.340371895 | 0.3458301 | —— | —— | —— |
| 19.516 | 6-Methyl-5-hepten-2-one | 0.517336217 | 3.723085 | 50 | 10.34672 | 74.4617 |
| 21.234 | (Z)-3-Hexen-1-ol | 1.351795103 | 0.6865142 | 70 | 19.31136 | 9.807345 |
| 23.845 | Butanoic acid, (E)-3-hexenyl ester | 0.836827559 | 0.0454635 | —— | —— | —— |
| 24.51 | Acetic acid | 0.29634812 | 4.236291 | 22000 | 0.01347 | 0.192559 |
| 25.151 | (Z)-Hex-3-enyl 2-methylbutyrate | —— | 0.2875094 | —— | —— | —— |
| 28.739 | 3,5-Octadien-2-one | —— | 0.9019824 | —— | —— | —— |
| 38.103 | Oxime-, methoxy-phenyl-_ | 2.216780764 | —— | —— | —— | —— |
| 41.356 | Hexanoic acid | —— | 1.1290898 | 3000 | —— | 0.376363 |
| 42.697 | 1,3-cis,5-cis-Octatriene | —— | 0.5350521 | —— | —— | —— |
| 51.108 | Eugenol | 4.533423532 | —— | 0.71 | 6385.104 | —— |

Note: —— is not found.
